# Supplementary material for: Rapid realist review of the role of community pharmacy in the public health response to COVID-19
Source: BMJ Open. 2021 Jun 16;11(6):e050043. doi: 10.1136/bmjopen-2021-050043 (PMC8210681; doi:10.1136/bmjopen-2021-050043)
Supplement: Supplementary data [file bmjopen-2021-050043supp001.pdf]

Appendix 1 - OVID MEDLINE – Search Strategy

1. exp Coronavirus/ or exp Coronavirus Infections/ or (coronavirus* or "2019-nCoV" or "2019 ncov" or nCov or "Covid 19" or Covid19 or "SARS CoV 2" or novel coronavirus or novel corona virus or covid* or "coronavirus 2" or coronavirus infection* or coronavirus disease or corona virus disease or new coronavirus or new corona virus or new coronaviruses or novel coronaviruses or wuhan).mp. [mp=title, abstract, original title, name of substance word, subject heading word, floating sub-heading word, keyword heading word, organism supplementary concept word, protocol supplementary concept word, rare disease supplementary concept word, unique identifier, synonyms]

2. (Community pharmacy or community pharmacies or community pharmacist or community pharmacists or retail pharmacy or retail pharmacist or retail pharmacists or dispensing chemist or dispensing chemists or (((Pharmacist or pharmacy) and (community or primary care)) or dispensary or apothecary or druggist)).mp. [mp=title, abstract, original title, name of substance word, subject heading word, floating sub-heading word, keyword heading word, organism supplementary concept word, protocol supplementary concept word, rare disease supplementary concept word, unique identifier, synonyms]

3. 1 and 2

NB. No Date or Language Limits were applied.
