# Supplementary material for: Rapid realist review of the role of community pharmacy in the public health response to COVID-19
Source: BMJ Open. 2021 Jun 16;11(6):e050043. doi: 10.1136/bmjopen-2021-050043 (PMC8210681; doi:10.1136/bmjopen-2021-050043)
Supplement: Supplementary data [file bmjopen-2021-050043supp002.pdf]

Appendix 2 - Literature inclusion criteria

Published after January 2003 AND

COVID-19 or other pandemic or other infectious diseases

OR vaccination programmes

OR expanded/extended roles

AND Community Pharmacy

AND High- or middle-income country
