# Supplementary material for: Rapid realist review of the role of community pharmacy in the public health response to COVID-19
Source: BMJ Open. 2021 Jun 16;11(6):e050043. doi: 10.1136/bmjopen-2021-050043 (PMC8210681; doi:10.1136/bmjopen-2021-050043)
Supplement: Supplementary data [file bmjopen-2021-050043supp003.pdf]

PRISMA 2009 Flow Diagram

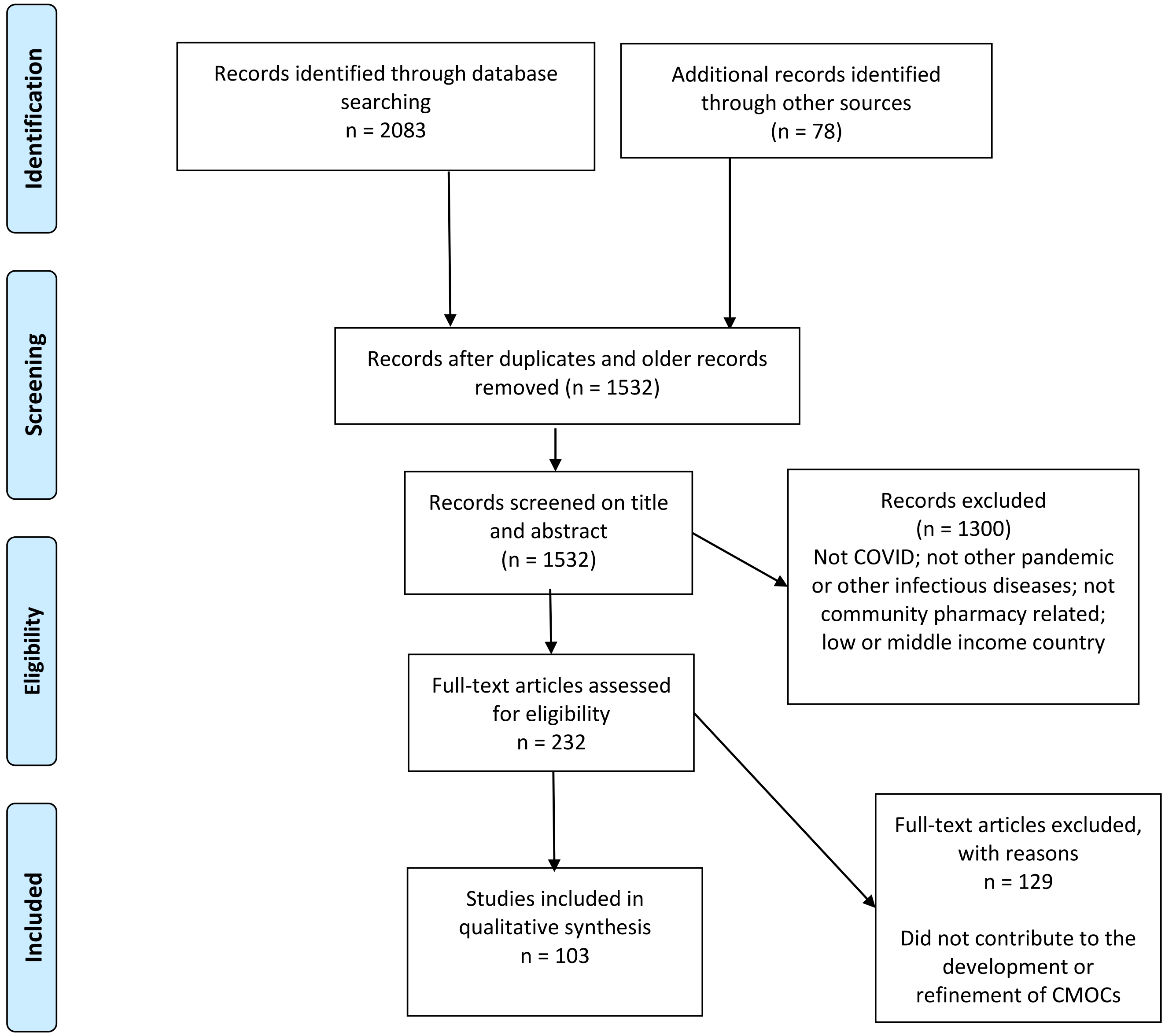

From: Moher D, Liberati A, Tetzlaff J, Altman DG, The PRISMA Group (2009). *P*referred *R*eporting *I*tems for *S*ystematic Reviews and *M*eta-*A*nalyses: The PRISMA Statement. PLoS Med 6(7): e1000097. doi:10.1371/journal.pmed1000097

For more information, visit www.prisma-statement.org.
