# Supplementary material for: Rapid realist review of the role of community pharmacy in the public health response to COVID-19
Source: BMJ Open. 2021 Jun 16;11(6):e050043. doi: 10.1136/bmjopen-2021-050043 (PMC8210681; doi:10.1136/bmjopen-2021-050043)

Appendix 4 - A simplified diagram of the programme theory of a COVID-19 vaccination programme provided by community pharmacies

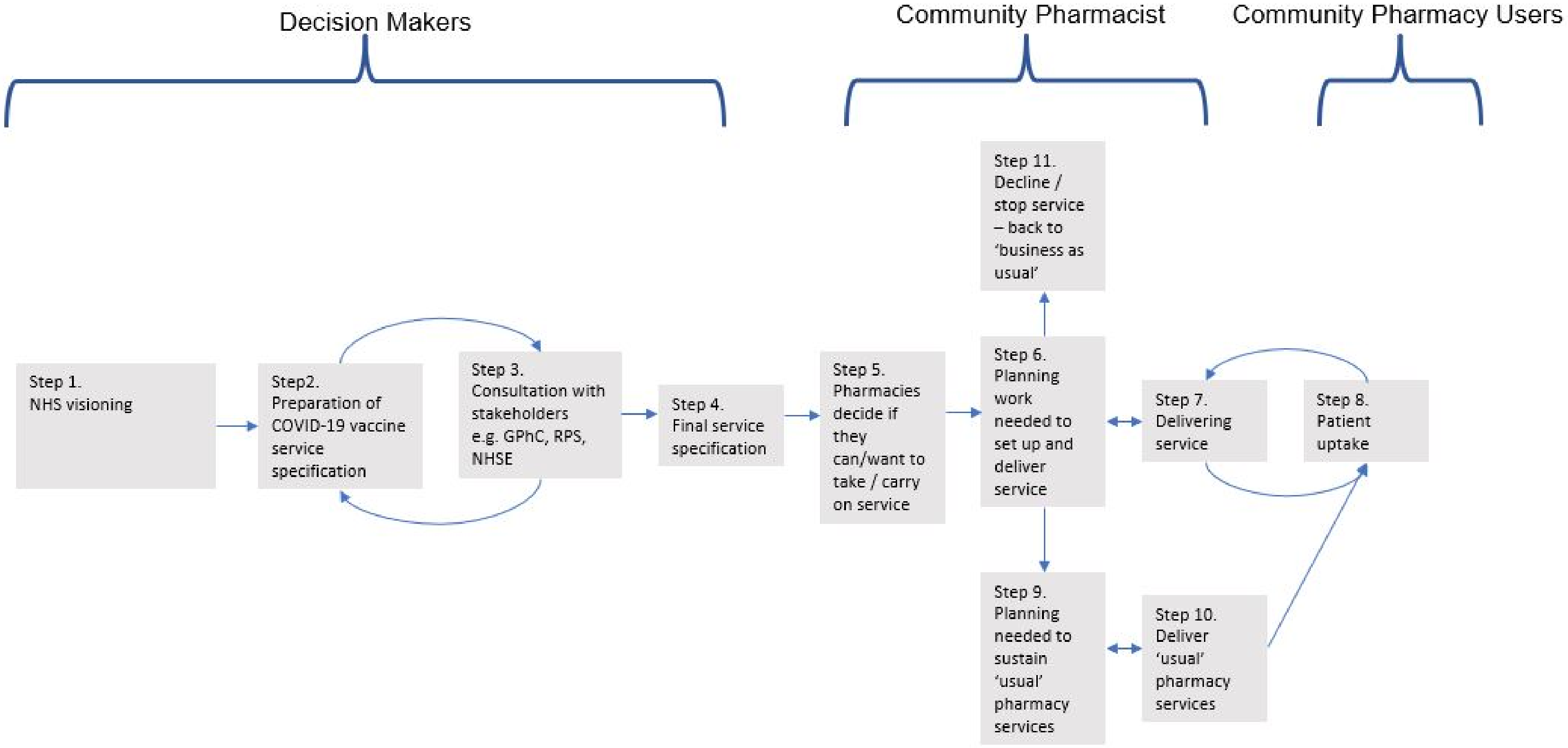

Supplement: Supplementary data [file bmjopen-2021-050043supp004.pdf]
