# Supplementary material for: Rapid realist review of the role of community pharmacy in the public health response to COVID-19
Source: BMJ Open. 2021 Jun 16;11(6):e050043. doi: 10.1136/bmjopen-2021-050043 (PMC8210681; doi:10.1136/bmjopen-2021-050043)

BMJ Open

BMJ Open is committed to open peer review. As part of this commitment we make the peer review history of every article we publish publicly available.

When an article is published we post the peer reviewers' comments and the authors' responses online. We also post the versions of the paper that were used during peer review. These are the versions that the peer review comments apply to.

The versions of the paper that follow are the versions that were submitted during the peer review process. They are not the versions of record or the final published versions. They should not be cited or distributed as the published version of this manuscript.

BMJ Open is an open access journal and the full, final, typeset and author-corrected version of record of the manuscript is available on our site with no access controls, subscription charges or pay-per-view fees (http://bmjopen.bmj.com).

If you have any questions on BMJ Open's open peer review process please email info.bmjopen@bmj.com

BMJ Open

A Rapid Realist Review of the Role of Community Pharmacy in the Public Health Response to COVID-19

Journal:	*BMJ Open*
Manuscript ID	bmjopen-2021-050043
Article Type:	Original research
Date Submitted by the Author:	09-Feb-2021

Complete List of Authors:	Maidment, Ian; Aston University<br>Young, Emma; The University of Sheffield<br>MacPhee, Maura; The University of British Columbia<br>Booth, Andrew; The University of Sheffield, School of Health & Related Research (ScHARR)<br>Zaman, Hadar; University of Bradford<br>Breen, Juanita; University of Tasmania<br>Hilton, Andrea; University of Hull, FHSC<br>Kelly, Tony; Aston University, PPI Lead on Project<br>Wong, Geoffrey; University of Oxford, Primary Care Health Sciences

Keywords:	COVID-19, QUALITATIVE RESEARCH, Organisation of health services < HEALTH SERVICES ADMINISTRATION & MANAGEMENT

SCHOLARONE™
Manuscripts

I, the Submitting Author has the right to grant and does grant on behalf of all authors of the Work (as defined in the below author licence), an exclusive licence and/or a non-exclusive licence for contributions from authors who are: i) UK Crown employees; ii) where BMJ has agreed a CC-BY licence shall apply, and/or iii) in accordance with the terms applicable for US Federal Government officers or employees acting as part of their official duties; on a worldwide, perpetual, irrevocable, royalty-free basis to BMJ Publishing Group Ltd ("BMJ") its licensees and where the relevant Journal is co-owned by BMJ to the co-owners of the Journal, to publish the Work in this journal and any other BMJ products and to exploit all rights, as set out in our licence.

The Submitting Author accepts and understands that any supply made under these terms is made by BMJ to the Submitting Author unless you are acting as an employee on behalf of your employer or a postgraduate student of an affiliated institution which is paying any applicable article publishing charge ("APC") for Open Access articles. Where the Submitting Author wishes to make the Work available on an Open Access basis (and intends to pay the relevant APC), the terms of reuse of such Open Access shall be governed by a Creative Commons licence – details of these licences and which Creative Commons licence will apply to this Work are set out in our licence referred to above.

Other than as permitted in any relevant BMJ Author's Self Archiving Policies, I confirm this Work has not been accepted for publication elsewhere, is not being considered for publication elsewhere and does not duplicate material already published. I confirm all authors consent to publication of this Work and authorise the granting of this licence.

For peer review only

A Rapid Realist Review of the Role of Community Pharmacy in the Public Health Response to COVID-19

Ian Maidment (corresponding author), Aston University, Birmingham, B4 7ET, UK, i.maidment@aston.ac.uk, Phone/fax: 0121-204-3000; Emma Young, University of Sheffield, Sheffield, UK; Maura MacPhee, University of British Columbia, Vancouver, Canada; Andrew Booth, University of Sheffield, Sheffield, UK; Hadar Zaman, University of Bradford, Bradford, UK; Juanita Breen, University of Tasmania, Hobart; Andrea Hilton, University of Hull, Hull, UK; Tony Kelly, PPI Lead (Aston University), Birmingham, UK; Geoff Wong, University of Oxford, Oxford, UK.

Keywords: COVID-19, SARS-CoV-2, Community Pharmacy Services, Vaccination

Word count: 3,521

For peer review only

Introduction

Community pharmacists and their teams have remained accessible to the public providing essential services despite immense pressures during the COVID-19 pandemic. They have successfully expanded the influenza vaccination programme and are now supporting the delivery of the COVID-19 vaccination roll-out.

Aim

This rapid realist review aims to understand how community pharmacy can most effectively deliver essential and advanced services, with a focus on vaccination, during the pandemic and in the future.

Method

An embryonic programme theory was generated using four diverse and complementary documents along with the expertise of the project team. Academic databases, preprint services and grey literature were searched and screened for documents meeting our inclusion criteria. The data was extracted from 103 documents to develop and refine a programme theory using a realist logic of analysis. Our analysis generated 13 context-mechanism-outcome configurations explaining when, why and how community pharmacy can support public health vaccination campaigns, maintain essential services during pandemics, and capitalise on opportunities for expanded, sustainable public health service roles. The views of stakeholders including pharmacy users, pharmacists, pharmacy teams and other healthcare professionals were sought throughout to refine the 13 explanatory configurations.

Results

The 13 context-mechanism-outcome configurations are organised according to decision makers, community pharmacy teams and community pharmacy users as key actors. Review findings include: supporting a clear role for community pharmacies in public health; clarifying pharmacists' legal and professional liabilities; involving pharmacy teams in service specification design; providing suitable

For peer review only - http://bmjopen.bmj.com/site/about/guidelines.xhtml

guidance, adequate compensation and resources; and leveraging accessible, convenient locations of community pharmacy.

Discussion

Community pharmacy has been able to offer key services during the pandemic. Decision makers must endorse, articulate and support a clear public health role for community pharmacy. We provide key recommendations for decision makers to optimise such a role during these unprecedented times and in the future.

Strengths and limitations of this study

A diverse group of professional and public stakeholders validated our findings from the literature.

By using a realist approach, we were to use broad range of data, including grey literature.

To enable us to develop recommendations in a timely manner the focus of the review was deliberately narrow.

The topic could have been informed by other sources of evidence in particular empirical interviews with key stakeholder.

COVID-19 vaccination is a rapidly evolving area and this research was based on the best available evidence at the time.

For peer review only - http://bmjopen.bmj.com/site/about/guidelines.xhtml

For peer review only

For peer review only - http://bmjopen.bmj.com/site/about/guidelines.xhtml

Introduction

Community pharmacy teams have continued to provide essential services during the coronavirus disease 2019 (COVID-19) pandemic. They offer accessibility and medicines expertise to the public, even in challenging times.[1 2 3] However, COVID-19 creates extra workload demands, such as medication dispensing with increases of up to 33% in prescription numbers.[4] To cope with this demand community pharmacies have increased their opening hours and hired additional staff.[4] Alongside this additional workload, they have managed widening coverage of the influenza vaccines programme.[5]

Evidence suggests that community pharmacy can successfully provide diverse vaccination services including seasonal and pandemic influenza, travel vaccinations and hepatitis B for at-risk groups, within the provisions of the UK National Health Service (NHS) or privately.[6] They have successfully provided influenza vaccines as an NHS commissioned advanced service since 2015.[5] One service evaluation found that of 485 patients asked, 99% expressed confidence in their pharmacist to provide additional vaccinations.[7] Community pharmacy can also support influenza and other vaccinations to combat the significantly higher COVID-19 related mortality in the non-white community.[8 9]

The COVID-19 pandemic has stretched NHS capacity to safely and efficiently meet public health demands. A role for community pharmacy in the national vaccination service requires an understanding of what pharmacy teams require to successfully deliver essential and advanced services during the pandemic. Such knowledge is timely, given the roll-out of COVID-19 vaccines across the community.[10]

Delivering a vaccine is a complex process and successful delivery is context-dependent. A realist review helps make sense of complex situations[11 12], such as how community pharmacy can most

For peer review only - http://bmjopen.bmj.com/site/about/guidelines.xhtml

effectively address the challenges presented by COVID-19. A rapid review can generate guidance for decision makers to assist with roll-out of COVID-19 vaccinations to community pharmacy.

Methods

A rapid realist review of academic and other literature, supplemented by input from key actors, was undertaken to understand how and when community pharmacy can effectively support the public health agenda during pandemics such as COVID-19. Rapid reviews aim to ensure findings are generated and disseminated in response to the urgent nature of the situation. To produce this knowledge at pace, we truncated the following review processes:

- Programme theory development was undertaken within 1 month with input from the project team.
- Searching was expedited using broad search terms and using a limited number of key data sources.
- Data analysis and context-mechanism-outcome configuration (CMOC) development focussed on where the programme theory was considered most important during COVID-19.

This realist review was undertaken within six-months (August 2020 - January 2021), the protocol was published on PROSPERO[13] and, where relevant, follows the RAMESES quality and publication standards.[14]

Stage 1: Programme Theory Development

The project team met virtually to develop an embryonic programme theory using four diverse documents from an initial search representing a professional journal[15], a research journal[16], a policy document (Royal Pharmaceutical Society)[17] and a practical influenza briefing.[18] The team identified the need for (i) enabling guidance for community pharmacy (to achieve legitimisation)[16]; (ii) practical direction for community pharmacy practices (to ensure feasibility)[15 16]; and (iii) user assurance of appropriate, safe, feasible and timely intervention (relative advantage).[15] The resultant embryonic

For peer review only - http://bmjopen.bmj.com/site/about/guidelines.xhtml

114 theory, patterned on a COM-B behavioural model of capability, opportunity, and motivation leading to behaviour[19], was used to inform searching and initial analyses.

Stage 2: Literature Searching

Searches were conducted (July-August 2020) using MEDLINE, EMBASE, CINAHL, Web of Science, and Scopus for search concepts relating to Pharmacy and COVID by AB (see Appendix 1 for search strategy). Reference checking and citation searching of all included references on Google Scholar (using the Publish or Perish tool) were also carried out. Given the novelty of the virus, we searched the contents of preprint services and the World Health Organisation (WHO) COVID Register. Grey literature searches included social media (e.g. blogs, facilitated Twitter® discussion [#Cpharmchat]), community pharmacy websites, and emails from relevant regulators and professional organisations (e.g., Royal Pharmaceutical Society [RPS], Pharmaceutical Services Negotiation Committee [PSNC], General Pharmaceutical Council [GPhC]).

Key inclusion criteria were high or middle-income countries, community pharmacy and infectious disease management (see Appendix 2). The search covered January 2003-July 2020 to include SARS, a comparable condition first identified in 2003. There were no restrictions on study designs eligible for inclusion.

Stage 3: Data Selection and Extraction (selection and coding)

Selection and appraisal of documents followed a two-step procedure:

1). EY screened the title, abstract and keywords of potentially relevant documents against inclusion criteria. A 10% random sample was checked by two research team members (AB and MM) for consistency.

2). EY obtained and screened full texts of all documents meeting the eligibility criteria.

For peer review only

Relevant data from the included full text documents was coded into NVivo by EY, MM and JB. Some codes came from the data (i.e. inductive coding); others were derived from the programme theory (i.e. deductive coding) and some were derived using retroduction (i.e. by interpretation of what might be functioning as mechanisms).[20] No assessment was made of the rigour of the data within included documents, however global judgements were made of the quality of the explanations provided by the CMOCs and programme theory using the criteria of consilience, simplicity and analogy.[21]

Stage 4: Data Synthesis

The data analysis/synthesis was conducted by EY, MM and JB with input from the rest of the project team to develop and refine the programme theory using a realist logic of analysis. Our analysis generated 13 realist CMOCs, explaining when, why and how community pharmacy can support public health vaccination campaigns, maintain essential services during pandemics, and capitalise on opportunities for expanded, sustainable public health service roles. Actor conversations generated further CMOCs related to care for diverse and vulnerable populations, including the Black and Minority Ethnic (BAME) community.

Our realist logic of analysis centred on the following questions:

- Interpretation of meaning: Do the contents coded by the team provide data that may be interpreted as CMOCs?
- Interpretations and judgements about CMOCs: How do the CMOCs relate to the programme theory?
- Interpretations and judgements about programme theory: How do the programme theory and its CMOCs correspond with key actor perspectives of reality?

For peer review only - http://bmjopen.bmj.com/site/about/guidelines.xhtml

Data to answer our questions was iteratively sought across documents. Interpretive cross-case comparison was used to identify and to explain the “success” of pandemic community pharmacy interventions delivered in different settings or to different population groups.

Key Stakeholders

Key stakeholders, including community pharmacists and support staff (including representatives from large and smaller chains, sole independent pharmacies and primary care), other healthcare professionals and members of the public were consulted on four occasions. The meetings took place over Microsoft Teams and each lasted about one hour.

Patient and Public Involvement

Members of the public were drawn from The University of Sheffield’s Patient and Public Involvement database and contacts of the core project team. Groups numbered between 11 and 13 members, with ages ranging from 22 to 74 years, from diverse locations and ethnicities including Black African, Black Caribbean, British Asian, British Chinese, White Irish and White British. Collectively, stakeholders provided feedback and advice on their real-world experience of working in or using community pharmacy.

Results

103 documents were included in this rapid review and were coded to develop and refine our CMOCs and programme theory (PRISMA diagram in Appendix 3). The final programme theory is summarised in Appendix 4—from abstract visioning to actual patient uptake of the COVID-19 vaccine. Although the programme theory is outlined in a linear fashion, steps within it are not necessarily linear and may occur simultaneously. The CMOCs are organized according to key actors, or individuals and groups with a vested interest in community pharmacy delivery of a COVID-19 vaccination

For peer review only - http://bmjopen.bmj.com/site/about/guidelines.xhtml

182 programme. “Actor” derives from sociology and is synonymous with “stakeholder”; we privileged
183 this term to differentiate programme theory/CMOC organization from project stakeholder
184 participants.[22] Table 1 briefly describes the three actor groups with their respective CMOCs and
185 corresponding steps in Appendix 4. Tables 2-4 show the final 13 CMOCs.

186

187 Table 1: Programme Theory Actors with corresponding CMOCs and steps

Programme Theory Actors	Description	CMOCs*	Steps in Programme Theory**
Decision Makers	The UK government, regulatory and professional bodies, the public.	CMOC 1 - 4	Steps 1 - 4
Community Pharmacists and their Teams	Community pharmacists are health care professionals registered by the General Pharmaceutical Council and supported by teams made up of counter assistants, dispensers and registered technicians. They work in high street locations, in local communities and in supermarkets. Employers range from large chains to small individually owned community pharmacies.	CMOC 5 – 9	Steps 5 – 7 and 9 – 11
Pharmacy Users	Members of the public who use any community pharmacy services including prescription dispensing, minor ailment advice/treatment or vaccination services.	CMOC 10 - 13	Step 8

188

189 *see Table 2-4

190 **See Appendix 4

For peer review only - http://bmjopen.bmj.com/site/about/guidelines.xhtml

The following sections summarise the CMOCs related to each of the three actor groups. Illustrative examples of the supporting evidence from review documents are presented (Tables 2-4).

Decision makers

Decision maker and public endorsement (CMOC 1) are essential first steps in enlisting community pharmacy for COVID-19 vaccination programmes. Regulators must ensure pharmacists have the legal scope to do so (CMOC 2), with community pharmacy input during the development of policies and protocols (CMOC 3), so that final service specifications are flexible and do-able within local settings (CMOC 4).

Table 2 - Decision Makers (CMOCs 1-4)

**CMOC 1 – Support a public health role**[23 24 25 26-32]	**When the government, pharmacy regulators, professional bodies and the public endorse and support a clear role for community pharmacy in public health services (C), community pharmacists will be more likely to adopt vaccination services (O) because they see it as their professional role and duty (M).**
	"Distribution and administration of the COVID-19 vaccination programme will require concerted action across the NHS. With unique insight and expertise in medicines and the delivery of vaccination programmes, pharmacists have a clear role in contributing to the success of this programme." (Great Britain)[24]<br><br>*"55% of the public have visited a pharmacy during the COVID19 crisis…89% of people believe pharmacies are playing an essential role in the COVID19 crisis." (UK National Pharmacy Association)*[23]

For peer review only - http://bmjopen.bmj.com/site/about/guidelines.xhtml

	"Pharmacists…have been called on to coordinate the administration of COVID-19 tests...providing ongoing COVID-19 surveillance to communities by allowing walk-in testing at community pharmacies…[This] might be more sustainable and convenient than the large-scale public screening being done as of the summer of 2020." (USA)[25]<br><br>*"Given the past success of community pharmacists with increasing annual seasonal influenza uptake and their accessibility, pharmacists will need to be central in administering COVID-19 vaccines in order to achieve rapid population-wide coverage." (Canada)*[26]
**CMOC 2 –Clarify legal and professional liabilities**[24 31 33-40]	**When pharmacy regulators and the NHS clarify community pharmacists' legal and professional liabilities arising from the administration of a novel and potentially unlicensed COVID-19 vaccine (C), community pharmacists are more willing to give the vaccination (O) because they feel reassured regarding liability (M).**
	"The role of pharmacists in the COVID-19 vaccination programme must be made clear to the pharmacy profession itself. Professional and representative pharmacy bodies have an important role to play in providing the right level of information to the profession to support their roles in the vaccination programme." (Great Britain)[24]<br><br>*"Indemnity insurance for individual healthcare professionals needs to be amended to cover this activity and be state-funded. There also needs to be clear communication to healthcare professionals, so they clearly understand*

For peer review only - http://bmjopen.bmj.com/site/about/guidelines.xhtml

	that they are covered and under which circumstances this applies." (Great Britain)[41]
**CMOC 3 – Co-develop feasible service specifications**[1 38 42-48]	**When COVID-19 vaccination policy and service specifications have been developed with input from diverse community pharmacists and staff tasked with administering and supporting the administration of the vaccine (C), community pharmacies are more likely to deliver the service (O) because they believe the service specification is feasible (M).**
	"Pharmacists ideally want input into future policy changes before they are finalized, so that these can reflect capacity and preparedness on the ground and be publicized accurately." (Great Britain)[1]
**CMOC 4 – Issue clear, relevant and timely guidance**[25 27 31 34 48-62]	**When government, pharmacy regulators and professional bodies provide consistent, clear, relevant, and timely guidance for the delivery of the COVID-19 vaccines (C), community pharmacies are more likely to deliver the service (O), because the guidance is helpful and simplifies implementation (M).**
	"… it is good to see that NHSE/I have provided the information we have been waiting for to review the resources we have nationwide and decide how we can bring them to bear to help the NHS defeat this virus." (Great Britain)[49]

202

203 In England community pharmacies have government contracts and partnerships to deliver
204 vaccinations and other essential services during emergencies, including the COVID-19 pandemic.[25 31
205 32] Pharmacies in the USA and Canada have also been identified as having a substantive role in
206 vaccine administration (CMOC1).[25,26] The idea of harnessing UK community pharmacy capacity
207 enjoys widespread public support (CMOC2).[23] However, appropriate service delivery is hampered by
208 unfeasible operational conditions (CMOC3)[1]; for example, medication deliveries are funded by the
209 UK government for "vulnerable people", but this category of service users is defined narrowly by the

For peer review only - http://bmjopen.bmj.com/site/about/guidelines.xhtml

210 government and misunderstood by the public, creating unrealistic expectations of community
211 pharmacy and generating additional work.[1]

212 In contrast, when clear options for community pharmacy involvement in COVID-19 vaccination
213 programmes were issued through the NHS (CMOC4), the chief executive of the Association of
214 Independent Multiple Pharmacies commented positively on members' engagement in delivering
215 vaccines.[49]

216 *Community Pharmacists and Team*

217 Community pharmacies have had to manage ongoing essential services, in addition to supporting
218 COVID vaccination delivery during the pandemic (CMOC6), including delivery of necessary
219 medications (CMOC7). Given fears and anxieties related to COVID-19 and changes to service
220 delivery, community pharmacies have had to deal with inappropriate behaviours from the public,
221 including emotional abuse and threats of physical abuse (CMOC8). As an integral public health
222 service, community pharmacy capacity to meet NHS needs will be enhanced through use of IT and
223 collaboration with other service providers (CMOC9).

224 Table 3 - Community Pharmacists and Team (CMOCs 5-9)

**CMOC 5 – Receive adequate compensation and resources**[1 63-77]	**When community pharmacies receive adequate compensation and resource support for COVID-19 vaccines (C), they are more likely to deliver the service (O), because they feel recognised for their service contributions (M).**
	"Pharmacists…have been incredible in supporting patients throughout COVID-19 and rightly deserve recognition for the work they do. We know that teams were already under pressure and that colleagues in community pharmacy are feeling added financial strain." (Great Britain).[63]

For peer review only - http://bmjopen.bmj.com/site/about/guidelines.xhtml

	"In the community, mitigating the impact of COVID-19 has…focused largely on general practitioners (GPs). This is unsurprising to those in the pharmacy profession who have long considered policy makers to overlook them." (Great Britain)[1]
**CMOC 6 – Sustain capacity and facility to adapt essential services**[16 17 30 34 68 70 71 78-91 92]	**When a community pharmacy has the capacity and permission to manage and adapt existing essential services during COVID-19 (C), they are more likely to effectively deliver and sustain these services (O) because it's feasible for them to do so (M).**
	"Virtual and telephone consultations have become commonplace, particularly to vulnerable patients. Pharmacists have implemented systems to dispense medications in advance of need to minimise wait times and duplicate visits. In case-by-case examples…there has been anticipatory management of medication-related needs." (Ireland)[92]<br><br>*"Pharmacists who had comfort and confidence in managing electronic communication reported feeling greater control over workflow and the ability to triage and queue patients more effectively based on priority and need." (Canada)*[60]
**CMOC 7 – Inform users of essential service availability/continuity**	**When community pharmacies have processes in place to inform pharmacy users about the availability of essential services (C), pharmacy users are less likely to become anxious (O), because they feel reassured about access to what they need (M).**

For peer review only - http://bmjopen.bmj.com/site/about/guidelines.xhtml

92 16 28 31 32 51 60 65 71 80 81 85 93-101	*"Access to medications is a main concern expressed by our patients… The CDC and SAMHSA offer guidance on ways to reduce stress and anxiety in this time of uncertainty."*[95]<br><br>*"As a result of the power dynamics at play, it is ultimately up to pharmacists to be able to reassure patients and provide care, all while taking into account their mental health. Currently, guidelines regarding patient interaction during a pandemic are needed".*[51]<br><br>*"In addition, there is ongoing work in liaison with pharmacists, general practitioners, and state and territory authorities to enable therapeutic substitution by pharmacists in the event of a shortage. This will allow community pharmacists to substitute dose strength or form without prior approval from the prescriber, if a prescribed medicine is not available at the time of dispensing. These measures highlight the important role pharmacists can play in enabling and maintaining access to medicines for people in need throughout the COVID-19 outbreak." (Australia)*[85]<br><br>*"Access to medications is a main concern expressed by our patients… The CDC and SAMHSA offer guidance on ways to reduce stress and anxiety in this time of uncertainty." (USA)*[95]
**CMOC 8 – Protect the health and safety of staff** [24 31 51 71 77 81 102-108]	**When community pharmacies have the means to protect the health of pharmacists and staff (C), they are more likely to deliver essential services and COVID-19 vaccination services (O), because they feel safe to do so (M).**

For peer review only - http://bmjopen.bmj.com/site/about/guidelines.xhtml

	"…Given high levels of public anxiety and uncertainty regarding the integrity of the drug supply chain in Canada, many participants reported difficult, sometimes frightening, interactions with members of the public and their desire for dedicated security to provide support and conflict management" (Canada).[31]<br><br>*"An additional burden Asian pharmacists face, on top of pharmacist harassment, is the rise of anti-Asian racism that has come about due to COVID-19. Verbal and even physical abuse has been reported to happen in various countries, such as the UK, France, and the USA, to those of Chinese descent…" (UK, France, USA)*[51]<br><br>*"The survey found that although more than a third (37%) of pharmacists said they felt unsafe at some point working during the pandemic, concerns over PPE have eased. This is due, in part, to the supply problems being eased and community pharmacists being finally allowed to order from the national online PPE portal in an emergency, after calls for access from the RPS." (Great Britain)*[77]
**CMOC 9 – Enhance collaboration across services, including IT**[18 26 47 56 57 69 78 80 85 109-114]	**When systems needed for COVID-19 vaccination, including IT and remuneration, support collaboration by various service providers (e.g. general practitioner surgeries and community pharmacies: (C), a coordinated response is more likely (O), because reduced effort is needed from everyone (M).**

For peer review only - http://bmjopen.bmj.com/site/about/guidelines.xhtml

	"Planning and delivery should be undertaken across a consistent, pre-agreed footprint. It may be more efficient and cost effective to provide immunisation across a number of providers, pooling resources and sites to deliver the best service possible, and working in coordination with other local stakeholders such as directors of public health and local government." (Great Britain)[47]<br><br>*"For many community pharmacists, a lack of connected IT is a huge problem. Kieran Eason, who runs an independent pharmacy in Tamworth, Staffordshire, says lack of intra-operability makes it more difficult to do relatively simple things, like sending prescription requests to GPs. "Pharmacy IT is just a complete disaster," he says, suggesting the COVID-19 crisis has highlighted flaws, such as the number of different systems pharmacists use." (Great Britain)*[69]

Although attention is focused on a COVID-19 vaccine, community pharmacies offer diverse essential services. In the UK and other high-income countries, community pharmacy services also provide advanced services such as vaccinations.[115] Pharmacists consider it a professional responsibility to provide essential services during the pandemic, despite clear financial risks to themselves.[63]

Despite pharmacists' professional and moral obligations to provide essential services, ongoing persistent 'under-recognition' can jeopardize their ability to contribute to COVID-19 vaccinations (CMOC5) as well as maintain a usual service. Under-recognition has been an issue in previous UK vaccination campaigns; respondents to a survey of Welsh community pharmacists after the 2016 influenza season described providing a "mop up" service for general practitioners (GPs).[59 68]

For peer review only

For peer review only - http://bmjopen.bmj.com/site/about/guidelines.xhtml

Multiple required community pharmacy service adaptations have been reported by the UK Pharmaceutical Journal, including changeover in retail space to medication preparation and dispensing, and call-in shopping services for other retail items (CMOC6). Service adaptations have been affected by hours of operation, available staff, and cancellations of contracted services, such as blood pressure testing and smoking cessation support—all with financial implications for pharmacists.[80]

Before COVID-19, community pharmacies globally were offering "valued-added services" (VAS) such as drive-thru services, online ordering and communications services (e.g., prescription reminders) to stay competitive. Many VAS services have helped pharmacies adapt more quickly to pandemic restrictions.[81]

During the pandemic, ensuring access to needed medications has been a critical pharmacy service, to allay public concerns (CMOC7).[16] Potential and actual disruptions in expected services and needed supplies (e.g., medications) have resulted in tensions, threats and verbal/physical abuse by the public to community pharmacists (CMOC 8).[51 80 116] Safety policies, protocols and safety-related supplies (e.g., PPEs) must in place to ensure community pharmacy teams' safety.[80]

The UK COVID-19 vaccination campaign borrows heavily from previous, successful collaborative influenza vaccination programmes using community pharmacy and GPs (CMOC9).[48] Pooling resources improves service delivery.[117] For example, the UK Pharmaceutical Journal reported how a greater collaborative approach has resulted in successful influenza vaccination of home care staff and domiciliary workers during the pandemic.[117]

The pandemic has clearly demonstrated the importance of interoperable, connected IT systems across services.[118] Getting access to reliable information is important for tracking supplies and deliveries related to the COVID-19 vaccination programme.[69] A pandemic silver lining is raised awareness of IT functions for enhanced delivery of essential services (e.g., medication planning,

For peer review only - http://bmjopen.bmj.com/site/about/guidelines.xhtml

prescribing and dispensing between pharmacies and GPs) and advanced services, including vaccinations.[69]

Pharmacy Users

Pharmacy users trust community pharmacies as a reliable source of information (CMOC10) about vaccines, and pharmacies' local accessibility and convenience increases the likelihood of users obtaining COVID-19 vaccines through them (CMOC11). Community pharmacy relationships with vulnerable populations in their local settings may enhance uptake of the vaccine by these groups (CMOC12). Provision for privacy is an important user consideration. Pharmacy users expect private consultations to preserve their confidentiality (CMOC13).

For peer review only

For peer review only - http://bmjopen.bmj.com/site/about/guidelines.xhtml

Table 4 - Pharmacy Users (CMOCs 10-13)

**COMC 10 – Trust the pharmacist as reliable information source**[32 53 119-122]	**When pharmacy users receive information about the COVID-19 vaccine from their community pharmacy (C), they are more likely to get the vaccine (O), because they trust community pharmacy as a source of reliable, accurate information (M).**
	"When patients were educated about influenza, herpes zoster, and pneumococcal vaccines as a result of a pharmacist-driven intervention in community pharmacies, they were influenced to receive the vaccination." (USA)[119]<br><br>*"Being able to address the public enquiries with accurate up-to-date information about the local situation and the overall infection progress is the key to build trustful relationship with them at troubled times." (Macau)*[32]
**CMOC 11 – Trust the pharmacist to deliver responsive services**[7 18 23 26 34 37 43 51 54 59 63 70 84 85 123-135]	**When community pharmacies are trusted to make necessary service adaptations to ensure services are flexible, convenient and accessible (C), COVID-19 vaccine uptake is likely to be higher among pharmacy users (O) because the service is responsive to local needs (M).**
	"Pharmacists have always been the most accessible health care provider; this is especially true in the era of COVID-19….While other professionals have closed their doors to patients, community pharmacies remained open to the public despite stricter lockdown restrictions. As highly trusted healthcare clinicians, community pharmacists play a vital role in closing the gaps that are exacerbated by the additional strain on the system and reduced access to healthcare providers." (Canada)[51]

For peer review only - http://bmjopen.bmj.com/site/about/guidelines.xhtml

	"Pharmacy flu vaccination services complement those provided by general practitioners to help improve overall coverage and vaccination rates for patients in at-risk groups. These services are highly accessed by patients from all socio demographic areas, and seem to be particularly attractive to carers, frontline healthcare workers, and those of working age." (Great Britain)[135]
**CMOC 12 – Access culturally-sensitive services** *[27 78 80 92 136]*	**When community pharmacies leverage their community location and community-staff relationships (C), vulnerable populations, such as Black, Asian and Minority Ethnic groups are more likely to use their services, including COVID-19 vaccination services (O), because they trust their local community pharmacies to provide culturally sensitive service. (M)**
	"Research continues to highlight that patients who are medically under-served have poorer inequitable access to health care due to them experiencing greater physical barriers to accessibility, encountering poorer patient-professional communication and are significantly disadvantaged where a service is not tailored to their unique needs or preferences." (Great Britain) [137]<br><br>*"I think over the years what's happened is nationally it's almost like everything has to be the same, which then doesn't work because it doesn't accommodate all the little variations…So we need to go back and in each individual pharmacy, gear it towards the population that it is meant to be meeting the needs of" (Pharmacist caring for BAME community-Great Britain)*[137]

For peer review only

For peer review only - http://bmjopen.bmj.com/site/about/guidelines.xhtml

CMOC 13 – Receive private and confidential services[7 50 65 129 138-140]	**When community pharmacies make provisions for privacy (C), pharmacy users are more likely to use their services (O), because they are reassured about confidentiality (M).**
	"If there was something not right…the first thing I would do is make an appointment with a doctor. I wouldn't do and talk to somebody over a pharmacy counter." (Great Britain)[65]<br><br>*"Privacy, confidentiality and dignity are all vital elements of a trusting relationship between healthcare professionals and their patients…. In terms of privacy, the quality was perceived by the participants to include a confidential room that enabled private consultations." (Great Britain)*[7]

Public trust in community pharmacists is high—similar to doctors and nurses.[38] Trust between pharmacists and users can be leveraged to overcome scepticism about the COVID-19 vaccine. Providing reliable information about the disease and the vaccine, as pharmacies have done with other infections, can enhance public uptake of the COVID-19 vaccine.[113] In the USA, 90% of the US population lives within 5 miles of a community pharmacy. Given their convenience and accessibility, consumers have visited their community pharmacists 12 times more frequently than their GPs.[21] A UK study found that consumers who were eligible for a free influenza vaccine through their GPs were willing to pay for pharmacy service because of convenience and ease of access.[127] Established, trusting relationships are especially important for providing culturally sensitive services to marginalized and vulnerable communities.[129] Community pharmacists, often members of local communities are specially positioned to understand the culturally contextual factors that impact their pharmacy users.[89] Trusting relationships are founded on privacy, confidentiality and dignity,

For peer review only - http://bmjopen.bmj.com/site/about/guidelines.xhtml

and COVID-19, public health protocols and limited space must be considered—in order to maintain vital trust among pharmacy users.[65]

Discussion

This realist review sought to understand how community pharmacy can contribute to the public health agenda during the COVID-19 pandemic, particularly continuation of essential services and engagement in vaccination services.[115] As the COVID-19 vaccination service continues to evolve, our recommendations for decision makers highlight opportunities for community pharmacy to promote safe, efficient and effective service delivery.

Summary of key Findings

To optimise community pharmacy service during the pandemic, decision makers must endorse and articulate a clear role for these healthcare professionals. The public already endorses advanced roles for community pharmacy (e.g., vaccinations, minor ailment scheme), but public awareness depends on what decision makers do and say. Practical decision maker measures include adequate reimbursement to help cover the cost for time, staff and PPE (particularly for a sustainable long-term service); legal (including indemnity), regulatory coverage for advanced roles; and clear and consistent guidance for vaccination preparation and for adaptation of essential services. When given the opportunity, the permissions and resources to do so, community pharmacies have been able to adapt quickly to continue essential services and whenever possible, to offer critical advanced services. Historically, community pharmacies have significantly increased vaccination uptake (e.g., influenza vaccinations) given their accessibility and convenience and capacity to adapt to local needs for the general population and marginalized groups (e.g., BAME).

For peer review only - http://bmjopen.bmj.com/site/about/guidelines.xhtml

Compare to other similar or related studies discussing important differences in results

At the time of this realist review, there were no similar reviews on community pharmacy roles with respect to COVID-19 vaccinations. COVID-19 represents an unprecedented situation with limited direct evidence to guide decision-making. However, realist approaches engage with a wider evidence base, including research on past pandemics (e.g., SARS)[60 141 142], mass vaccination campaigns (e.g., influenza)[143], and community pharmacies' capacity pre-COVID-19 to deliver essential and advanced services.[19 144 145] Key factors previously include a lack of leadership, a lack of guidance and an increasing reliance on professional judgement and experience.[60] Research from the UK and other economically developed countries supports the 13 CMOCs and decision maker recommendations in this review (see tables 2-4 and table 5). However, limitations continue to surround the direct policy relevance of much of the community pharmacy evidence base.[146]

Study strengths and limitations

The realist approach uses diverse data, including grey literature. This feature is especially important given a novel and rapidly evolving topic area, such as COVID-19. Multiple researchers with subject matter expertise participated in screening the literature and extracting and coding data, which maximised opportunities to discuss and debate the plausibility of the inferences made. The CMOCs were developed and refined through regular discussions within a team with varied academic and clinical backgrounds. Professional and public stakeholder consultation further refined the CMOCs.

All rapid reviews operationalise coverage versus expediency. Other sources of evidence could have informed the review; however, potential gaps were mitigated by stakeholder engagement and expertise within the team. The evidence supporting the CMOCs was based on available time for document review, and during the review period, research, directives and policy related to COVID-19

For peer review only - http://bmjopen.bmj.com/site/about/guidelines.xhtml

331 vaccination rapidly evolved. The programme theory and its CMOCs, however, are expressed in such
332 a way that they can be further confirmed, refuted or refined in the future using additional data.

333 Meaning of the study: possible explanations and implications for decision makers

334 Although there are multiple actors involved in pandemic response, for brevity, recommendations
335 (Table 5) are directed towards decision makers who possess the formal authority to implement the
336 recommendations.

337

338 Table 5 - Recommendations for Decision Makers to Increase Community Pharmacy Engagement in
339 Pandemic Response (for further details check: https://www2.aston.ac.uk/lhs/research/periscope).

**Recommendations**	**Derived from CMOC**
Articulate a clear public health agenda role for community pharmacy (e.g., COVID-19 testing and vaccination)	1
Ensure pharmacy regulations for advanced roles, such as novel vaccine administration, are in place to legally protect community pharmacists and their teams	2
Involve local community pharmacies in policy and service specification development	3
Provide timely guidance with sufficient details for community pharmacies to quickly adapt to local needs	4
Provide adequate funding and reimbursement for community pharmacy services to deliver COVID-19 vaccines	5
Equip community pharmacies with the necessary permissions to manage and adapt essential services	6

For peer review only - http://bmjopen.bmj.com/site/about/guidelines.xhtml

Ensure community pharmacies have the means to adequately protect the health of themselves, their staff and pharmacy users	8
Facilitate collaboration and coordination of COVID-19 vaccination services across providers (e.g., GPs, community pharmacies) and systems (e.g., IT)	9

As the CMOCs were refined, 'tensions' were uncovered with implications for decision makers, particularly: community pharmacists as healthcare professionals versus retailers[43 106 145 147]; community pharmacies' capacity to remain financially viable while managing essential and advanced services.[19 148 149] and; pharmacists' capacity to provide off-site services while maintaining physical premises.[150] These tensions stem from lack of awareness of community pharmacists as healthcare professionals.

Along the continuum of healthcare from community to hospital, community pharmacy is often the first point of contact for the public. Community pharmacies contribute to primary care services through essential services (e.g., medication dispensing) and expanded roles (e.g., vaccinations),[115 147] often decreasing workload pressures on other providers, such as GPs.[151] Public surveys demonstrate high levels of satisfaction with community pharmacy services, and vaccination uptake is increased when pharmacies deliver these services.[149] Nevertheless, lack of public and decision maker awareness of community pharmacy primary care roles has slowed uptake and integration of these services.[121] A recent UK public survey found that community pharmacies are seen as "a medicine supply shop by 48.3% of people, as a place to purchase medicines by 22%, and a place to purchase non-medicinal products by 17.7%." [28]

In Ireland and Canada community pharmacies are an integral part of national vaccination campaigns.[149] In both instances, community pharmacists participate in vaccination planning, pharmacy regulators provide clear guidance on vaccination management, and vaccinations are

For peer review only - http://bmjopen.bmj.com/site/about/guidelines.xhtml

equitably refunded through public health systems. The removal of barriers, such as economic pressures on pharmacies has resulted in impressive national vaccine uptake, even in large countries, such as Canada.[149] Globally, there is an emerging trend for governments, health insurance companies and consumers to remunerate community pharmacies for services that contribute to improved health outcomes.[150]

Community pharmacies tend to be defined as premises where medications are dispensed, which compounds the confusion (by the public and decision makers) of professional primary care services versus retailers.[150] Instead, pharmacies should be defined with respect to actions or services that require specialised health knowledge to optimise health outcomes. For example, a retail approach to over-the-counter (OTC) medications is to permit consumers to make their own choices, similar to supermarket choices. Pharmacy input for into OTC purchases could potential decrease adverse medication interactions or unnecessary allergicreactions.[147] The 'value-add' of community pharmacies, evidence-informed engagement with consumers, can decrease morbidity and mortality outcomes and increase medication regimen adherence.[150 152]

Although community pharmacists can provide professional services off premises (e.g., immunizations in community and religious centres), they need to maintain their physical premises and staff for financial reasons. In addition, pharmacy users seek out community pharmacy services due to their accessibility and convenience, and without a physical space to engage regularly with pharmacy users, trust-building between pharmacists and users is compromised.[153]

As evidenced by public media, many countries, including the UK are declaring service specifications for COVID-19 vaccinations. Community pharmacists can potentially provide vaccination services in two locations.[154] They can collaborate with GPs in Primary Care Networks (PCN) to support PCN vaccination sites. Alternatively, they can provide a COVID-19 vaccination service from their premises

For peer review only

For peer review only - http://bmjopen.bmj.com/site/about/guidelines.xhtml

if they meet service specifications. The described tensions create difficult choices for pharmacists that can be ameliorated through the decision maker recommendations in Table 5.

Unanswered questions and future research

A future realist evaluation involving primary data collection from key actors will inform refinement of the programme theory, CMOCs and decision maker recommendations. This empirical research will address the tensions identified above. Future research will also address issues identified through the stakeholder groups, such as vaccination hesitancy, outside the scope of this review. While this work touched upon unique issues for marginalised populations including BAME groups, those of lower socioeconomic status and those with disabilities, further exploration is needed.

Conclusion

The COVID-19 pandemic is a worldwide health emergency. Vaccination is key to combatting the pandemic. The role of community pharmacy may be both short and long term; with a potential need for regular annual vaccines. This rapid realist review offers recommendations for decision makers to enable community pharmacy to play a key role, both during these unprecedented times and into the future.

For peer review only

407 ## Acknowledgement:

408 We acknowledge the contribution from professional and member of the public key stakeholder.

409 ## Funders

410 Jointly funded by UKRI and NIHR COV0176. The views expressed are those of the author(s) and not
411 necessarily those of the NIHR, UKRI or the Department of Health and Social Care.

412 ## Contributorship statement

413 IM conceived and led the project and had overall responsibility for PERISCOPE. All team members
414 developed develop the embryonic programme theory. AB conducted the searches. AB and GW
415 advised on realist methods. GW also advised on primary care implications and provided
416 methodological oversight. EY screened documents at title, abstract and full-text with support from
417 AB and MM. EY, MM and JB coded relevant data into NVivo. EY, MM and JB conducted the data
418 analysis/synthesis. HZ and AH advised on pharmacy aspects and policy implications. TK led PPI. TK
419 and HZ advised on implications in BAME communities. All team members contributed drafting the
420 final report for publication and approved the final draft for submission.

421 ## Data Sharing Statement

422 No additional data available.

423 ## Competing Interests

424 Geoff Wong is Deputy Chair of the NIHR HTA Prioritisation Committee: Integrated Community Health
425 and Social Care (A) and Member of the NIHR HTA Prioritisation Committee: Integrated Community
426 Health and Social Care (A) Methods Group.
427 Andrew Booth is a member of the National Institute for Health Research Health Services and
428 Delivery Research Funding Board, the National Institute for Health Research Evidence Synthesis

For peer review only

Programme Advisory Group and the National Institute for Health Research School for Social Care Research Commissioning Panel.

For peer review only

For peer review only - http://bmjopen.bmj.com/site/about/guidelines.xhtml

References

1. Parkhurst C, Singh Purewal G, Donyai P. Community Pharmacy and COVID-19—The Unsung Heroes on Our High Streets. *Journal of Patient Experience* 2020;7(3):282-84. doi: 10.1177/2374373520927638
2. Maidment I, Lawson S, Wong G, et al. Towards an understanding of the burdens of medication management affecting older people: the MEMORABLE realist synthesis. *BMC geriatrics* 2020;20(1):183. doi: 10.1186/s12877-020-01568-x [published Online First: 2020/06/06]
3. Barry HE, Hughes CM. Managing medicines in the time of COVID-19: implications for community-dwelling people with dementia. *International Journal of Clinical Pharmacy* 2020 doi: 10.1007/s11096-020-01116-y
4. Wickware C. Pharmacies' dispensing increases by up to a third as a result of COVID-19, survey finds. . *Pharm J* 2020:PJ April 2020 online. doi: 10.1211/pj.2020.20207917
5. Pharmaceutical Services Negotiating Committee. Flu Vaccination Service 2020 [Available from: https://psnc.org.uk/services-commissioning/advanced-services/flu-vaccination-service/ accessed 12/11/20.
6. Pharmaceutical Services Negotiating Committee. Vaccination Services 2020 [Available from: https://psnc.org.uk/services-commissioning/locally-commissioned-services/vaccination-services/08/12/2020.
7. Rendell T. Community pharmacist-led influenza vaccination: a service evaluation. *The Pharmaceutical Journal* 2020 doi: 10.1211/pj.2020.20208383
8. Public Health England. Beyond the data: Understanding the impact of COVID-19 on BAME groups. London: Public Health England, 2020.
9. Patel M. We must tear down the barriers to equality for BAME communities highlighted by COVID-19. *Pharmaceutical Journal* 2020

For peer review only

For peer review only - http://bmjopen.bmj.com/site/about/guidelines.xhtml

10. Robinson J. Ten things pharmacists should know about COVID-19 vaccines. *The Pharmaceutical Journal* 2020 doi: 10.1211/pj.2020.20208429

11. Wong G, Brennan N, Mattick K, et al. Interventions to improve antimicrobial prescribing of doctors in training: the IMPACT (IMProving Antimicrobial presCribing of doctors in Training) realist review. *BMJ Open* 2015;5(10):e009059. doi: 10.1136/bmjopen-2015-009059

12. Maidment ID, Lawson S, Wong G, et al. Health Services and Delivery Research. Medication management in older people: the MEMORABLE realist synthesis. Southampton (UK): NIHR Journals Library 2020.

13. Maidment I, Young E, Wong G, et al. A rapid realist review of community pharmacy support for the public health agenda during the COVID-19 pandemic and future health emergencies. PROSPERO 2020 CRD42020194078, 2020.

14. Wong G, Greenhalgh T, Westhorp G, et al. Development of methodological guidance, publication standards and training materials for realist and meta-narrative reviews: the RAMESES (Realist And Meta-narrative Evidence Syntheses – Evolving Standards) project. *Health Services and Delivery Research* 2014;2(30):1-252. doi: 10.3310/hsdr02300

15. Pharmacies across the UK close 'intermittently' to cope with increasing pressures from COVID-19. *The Pharmaceutical Journal* 2020 doi: 10.1211/pj.2020.20207844

16. Cadogan CA, Hughes CM. On the frontline against COVID-19: Community pharmacists' contribution during a public health crisis. *Research in Social and Administrative Pharmacy* 2021;17(1):2032-35. doi: 10.1016/j.sapharm.2020.03.015

17. Royal Pharmaceutical Society. RPS guidance on ethical, professional decision making in the COVID-19 Pandemic. London: Royal Pharmaceutical Society, 2020.

18. Pharmaceutical Services Negotiating Committee. PSNC Briefing 023/20: Early guidance on the 2020/21 Seasonal Influenza Vaccination Advanced Service 2020 [Available from: https://psnc.org.uk/services-commissioning/psnc-briefings-services-and-

For peer review only - http://bmjopen.bmj.com/site/about/guidelines.xhtml

commissioning/psnc-briefing-023-20-early-guidance-on-the-2020-21-seasonal-influenza-vaccination-advanced-service/.

19. Hattingh L, Sim TF, Sunderland B, et al. Successful implementation and provision of enhanced and extended pharmacy services. *Research in Social and Administrative Pharmacy* 2020;16(4):464-74. doi: 10.1016/j.sapharm.2019.06.015 [published Online First: 2019/07/06]

20. Greenhalgh T, Wong G, Westhorp G, et al. Retroduction in realist evaluation. [Internet]. Available from: http://ramesesproject.org/. The RAMESES II Project 2017.

21. Wong G. Data Gathering in Realist Reviews: Looking for needles in haystacks. Doing Realist Research: SAGE Publications Ltd, 2018:131-46.

22. Long N. Development sociology: actor perspectives. London: Routledge 2003.

23. National Pharmacy Association. Public see pharmacies as key to the future of healthcare in wake of pandemic 2020 [Available from: https://www.npa.co.uk/news-and-events/news-item/public-see-pharmacies-as-key-to-the-future-of-healthcare-in-wake-of-pandemic/ accessed 14 Jan 2021.

24. Royal Pharmaceutical Society. RPS Statement on the role of pharmacists in the COVID-19 Vaccination Programme London: Royal Pharmaceutical Society; 2020 [Available from: https://www.rpharms.com/Portals/0/FINAL_Statement%20on%20COVID-19%20Vaccination_Dec%202020_.pdf?ver=2020-12-02-143304-017 accessed 14/01/2021.

25. Strand MA, Bratberg J, Eukel H, et al. Community Pharmacists' Contributions to Disease Management During the COVID-19 Pandemic. *Preventing Chronic Disease* 2020;17 doi: 10.5888/pcd17.200317

26. Elbeddini A, Prabaharan T, Almasalkhi S, et al. Pharmacists and COVID-19. *Journal of Pharmaceutical Policy and Practice* 2020;13(1) doi: 10.1186/s40545-020-00241-3

For peer review only - http://bmjopen.bmj.com/site/about/guidelines.xhtml

27. Kirkdale CL, Nebout G, Taitel M, et al. Implementation of flu vaccination in community pharmacies: Understanding the barriers and enablers. *Annales Pharmaceutiques Françaises* 2017;75(1):9-16. doi: 10.1016/j.pharma.2016.08.006

28. Hindi AMK, Jacobs S, Schafheutle EI. Solidarity or dissonance? A systematic review of pharmacist and GP views on community pharmacy services in the UK. *Health & Social Care in the Community* 2018;27(3):565-98. doi: 10.1111/hsc.12618

29. Deslandes R, Evans A, Baker S, et al. Community pharmacists at the heart of public health: A longitudinal evaluation of the community pharmacy influenza vaccination service. *Research in Social and Administrative Pharmacy* 2020;16(4):497-502. doi: 10.1016/j.sapharm.2019.06.016

30. Sousa Pinto G, Hung M, Okoya F, et al. FIP's response to the COVID-19 pandemic: Global pharmacy rises to the challenge. *Research in Social and Administrative Pharmacy* 2021;17(1):1929-33. doi: 10.1016/j.sapharm.2020.07.004

31. Gregory PAM, Austin Z. COVID-19: How did community pharmacies get through the first wave? *Canadian Pharmacists Journal / Revue des Pharmaciens du Canada* 2020;153(5):243-51. doi: 10.1177/1715163520945741

32. Ung COL. Community pharmacist in public health emergencies: Quick to action against the coronavirus 2019-nCoV outbreak. *Research in Social and Administrative Pharmacy* 2020;16(4):583-86. doi: 10.1016/j.sapharm.2020.02.003

33. Burson RC, Buttenheim AM, Armstrong A, et al. Community pharmacies as sites of adult vaccination: A systematic review. *Human vaccines & immunotherapeutics* 2016;12(12):3146-59. doi: 10.1080/21645515.2016.1215393 [published Online First: 08/15]

34. Nadeem MF, Samanta S, Mustafa F. Is the paradigm of community pharmacy practice expected to shift due to COVID-19? *Research in Social and Administrative Pharmacy* 2021;17(1):2046-48. doi: 10.1016/j.sapharm.2020.05.021

For peer review only - http://bmjopen.bmj.com/site/about/guidelines.xhtml

35. Francis M, Hinchliffe A, Wales N. Vaccination services through community pharmacy. *Public Health Wales NHS Trust, Wales: NHS Wales* 2010

36. Clews G. Pharmacists able to administer unlicensed COVID-19 vaccines following legislation changes. *The Pharmaceutical Journal* 2020;305 doi: 10.1211/PJ.2020.20208453

37. Welsh Government, Department of Health N. I., Scottish Government, et al. Supporting pharmacists, pharmacy technicians and pharmacy teams throughout the second COVID-19 wave, 2020.

38. Wickware C. NHS England to choose 'selected' number of pharmacies to deliver at least 1,000 COVID-19 vaccines per week. *The Pharmaceutical Journal* 2020 doi: 10.1211/PJ.2020.20208608

39. Dyer C. Covid-19: Government poised to amend regulations to allow use of unlicensed vaccine. *The BMJ* 2020 doi: https://doi.org/10.1136/bmj.m3757

40. Cooper RJ. Pestle and mortal: the demise of community pharmacy in the UK. *International Journal of Pharmacy Practice* 2020;28(3):205-06. doi: 10.1111/ijpp.12635

41. Robinson J. Health professionals delivering unlicensed vaccines need government protection, say health bodies. *The Pharmaceutical Journal* 2020 doi: 10.1211/PJ.2020.20208381

42. Warner JG, Portlock J, Smith J, et al. Increasing seasonal influenza vaccination uptake using community pharmacies: experience from the Isle of Wight, England. *International Journal of Pharmacy Practice* 2013;21(6):362-67. doi: 10.1111/ijpp.12037

43. Hindi AMK, Schafheutle EI, Jacobs S. Community pharmacy integration within the primary care pathway for people with long-term conditions: a focus group study of patients', pharmacists' and GPs' experiences and expectations. *BMC Fam Pract* 2019;20(1):26-26. doi: 10.1186/s12875-019-0912-0

44. Pharmacy Voice, The Pharmaceutical Services Negotiating Committee, The Royal Pharmaceutical Society, et al. Consultation response: UK Influenza Pandemic Preparedness Strategy 2011. 2011

For peer review only - http://bmjopen.bmj.com/site/about/guidelines.xhtml

45. Bauman JL. Hero clinical pharmacists and the COVID-19 pandemic: Overworked and overlooked. *JACCP JAm Coll Clin Pharm* 2020

46. Stone JK, Pate AN. The Impact of COVID-19 Through the Eyes of a Fourth-Year Pharmacy Student. *American Journal of Pharmaceutical Education* 2020;84(6):ajpe8146. doi: 10.5688/ajpe8146

47. Royal College of General Practitioners. Delivering Mass Vaccinations During COVID-19 A Logistical Guide for General Practice. London: Royal College of General Practitioners, 2020.

48. NHS. Urgent preparing for General Practice to contribute to a potential COVID-19 Vaccination Programme. In: Service NH, ed. London, 2020.

49. NHS England to choose 'selected' number of pharmacies to deliver at least 1,000 COVID-19 vaccines per week. *The Pharmaceutical Journal* 2020 doi: 10.1211/pj.2020.20208608

50. Koster ES, Philbert D, Bouvy ML. Impact of the COVID-19 epidemic on the provision of pharmaceutical care in community pharmacies. *Research in Social and Administrative Pharmacy* 2020 doi: 10.1016/j.sapharm.2020.07.001

51. Elbeddini A, Wen CX, Tayefehchamani Y, et al. Mental health issues impacting pharmacists during COVID-19. *Journal of Pharmaceutical Policy and Practice* 2020;13(1) doi: 10.1186/s40545-020-00252-0

52. Bukhari N, Rasheed H, Nayyer B, et al. Pharmacists at the frontline beating the COVID-19 pandemic. *Journal of Pharmaceutical Policy and Practice* 2020;13 (1) (no pagination)(02000210) doi: http://dx.doi.org/10.1186/s40545-020-00210-w

53. Anon. Pharmacists on the frontline. *Canadian Pharmacists Journal* 2020;153(4):186-90. doi: 10.1177/1715163520930191

54. Farrell B, Tsuyuki RT. Pharmacists step up to the challenge of COVID-19. *Canadian Pharmacists Journal* 2020;153(4):185. doi: http://dx.doi.org/10.1177/1715163520930705

55. Bragazzi NL, Mansour M, Bonsignore A, et al. The Role of Hospital and Community Pharmacists in the Management of COVID-19: Towards an Expanded Definition of the Roles, Responsibilities, and Duties of the Pharmacist. *Pharmacy (Basel, Switzerland)* 2020

For peer review only

56. Chan AHY, Rutter V, Ashiru-Oredope D, et al. Together we unite: The role of the Commonwealth in achieving universal health coverage through pharmaceutical care amidst the COVID-19 pandemic. *Journal of Pharmaceutical Policy and Practice* 2020;13(1) doi: 10.1186/s40545-020-00214-6

57. Moore A. Pharmacists in one county say working with GP practices during the COVID-19 pandemic has been 'difficult'. *The Pharmaceutical Journal* 2020 doi: 10.1211/PJ.2020.20208556

58. Hoti K, Jakupi A, Hetemi D, et al. Provision of community pharmacy services during COVID-19 pandemic: a cross sectional study of community pharmacists' experiences with preventative …: Springer 2020.

59. NHS. Urgent preparing for Community Pharmacy to contribute to a potential COVID-19 Vaccination Programme. . In: Service NH, ed. London, 2020.

60. Austin Z, Martin JC, Gregory PAM. Pharmacy practice in times of civil crisis: The experience of SARS and "the blackout" in Ontario, Canada. *Research in Social and Administrative Pharmacy* 2007;3(3):320-35. doi: 10.1016/j.sapharm.2006.09.001

61. Chahine EB. The Role of Pharmacists in the Fight Against COVID-19. *Sr Care Pharm* 2020;35(7):286-91.

62. Jubraj B. Remote consultations: how pharmacy teams can practise them successfully: pharmaceutical-journal.com 2020.

63. Royal Pharmaceutical Society. All-Party Pharmacy Group warns of pressures on pharmacies London: Royal Pharmaceutical Society 2020 [Available from: https://www.rpharms.com/about-us/news/details/All-Party-Pharmacy-Group-warns-of-pressures-on-pharmacies#:~:text=The%20All%2DParty%20Pharmacy%20Group,continued%20pressures%20facing%20community%20pharmacies. accessed 14/01/2021.

For peer review only - http://bmjopen.bmj.com/site/about/guidelines.xhtml

64. Barnes E, Bullock A, Allan M, et al. Community pharmacists' opinions on skill-mix and delegation in England. *International Journal of Pharmacy Practice* 2017;26(5):398-406. doi: 10.1111/ijpp.12419

65. Saramunee K, Krska J, Mackridge A, et al. How to enhance public health service utilization in community pharmacy?: General public and health providers' perspectives. *Research in Social and Administrative Pharmacy* 2014;10(2):272-84. doi: 10.1016/j.sapharm.2012.05.006

66. Houle SKD, Grindrod KA, Chatterley T, et al. Paying pharmacists for patient care: A systematic review of remunerated pharmacy clinical care services. *Can Pharm J (Ott)* 2014;147(4):209-32. doi: 10.1177/1715163514536678

67. Burns C. Pharmacy staff in Scotland to receive £500 bonus in recognition of COVID-19 service. *the Pharmaceutical Journal* 2020 doi: 10.1211/PJ.2020.20208614 [published Online First: 12/2020]

68. Evans AM, Wood FC, Carter B. National community pharmacy NHS influenza vaccination service in Wales: a primary care mixed methods study. *British Journal of General Practice* 2016;66(645):e248-e57. doi: 10.3399/bjgp16x684349

69. Moore A. No going back: how the pandemic is changing community pharmacy. *The Pharmaceutical Journal* 2020;305 doi: 10.1211/PJ.2020.20208309

70. Merks P, Jakubowska M, Drelich E, et al. The legal extension of the role of pharmacists in light of the COVID-19 global pandemic. *Research in Social and Administrative Pharmacy* 2020 doi: 10.1016/j.sapharm.2020.05.033

71. Zaidi STR, Hasan SS. Personal protective practices and pharmacy services delivery by community pharmacists during COVID-19 pandemic: Results from a national survey. *Research in Social and Administrative Pharmacy* 2020

72. Anon. Pharmacies must be at the forefront of the COVID-19 vaccination campaign. *The Pharmaceutical Journal* 2020;305 doi: 10.1211/PJ.2020.20208546

For peer review only - http://bmjopen.bmj.com/site/about/guidelines.xhtml

73. Robinson J. England has lowest number of community pharmacies in five years, figures show. *The Pharmaceutical Journal* 2020 doi: 10.1211/PJ.2020.20208569

74. Wickware C. Pharmacies to be paid 50p more per flu vaccination in 2020/2021. *The Pharmaceutical Journal* 2020 doi: 10.1211/PJ.2020.20208362

75. Doyle-Price J. Pharmacies have an important role in this pandemic – but they need help to continue saving lives. *The Telegraph* 2020.

76. Robinson J. Up to 85% of community pharmacies could be in financial deficit by 2024, economic analysis concludes. *The Pharmaceutical Journal* 2020 doi: 10.1211/PJ.2020.20208332

77. Wilkinson E, Connelly D. Is pharmacy prepared for a resurgence of COVID-19? *The Pharmaceutical Journal* 2020;305 doi: 10.1211/PJ.2020.20208340

78. Hayden JC, Parkin R. The challenges of COVID-19 for community pharmacists and opportunities for the future. *Irish Journal of Psychological Medicine* 2020;37(3):198-203. doi: 10.1017/ipm.2020.52

79. Mercadante AR, Yokota M, Hwang A, et al. Choosing Evolution over Extinction: Integrating Direct Patient Care Services and Value-Based Payment Models into the Community-Based Pharmacy Setting. *Pharmacy* 2020;8(3):128. doi: 10.3390/pharmacy8030128

80. Stewart K, Barai R, Priarities N. How to keep your community pharmacy running during the COVID-19 pandemic2020.

81. Hussain R, Dawoud DM, Babar Z-U-D. Drive-thru pharmacy services: A way forward to combat COVID-19 pandemic. *Research in Social and Administrative Pharmacy* 2021;17(1):1920-24. doi: 10.1016/j.sapharm.2020.07.015

82. Broughel J, Yatsyshina Y. Relax Pharmacy Regulations to Help with COVID-19 Testing and Treatment. *SSRN Electronic Journal* 2020 doi: 10.2139/ssrn.3564463

83. Pritchard RI, Huff J, Scheinberg N. Impact of Regulatory Changes on Pharmacist Delivered Telehealth During the COVID-19 Pandemic. *Journal of the American Pharmacists Association* 2020;60(6):e76-e79. doi: 10.1016/j.japh.2020.06.004.

For peer review only - http://bmjopen.bmj.com/site/about/guidelines.xhtml

84. Lynch M, Leary A. COVID-19 related regulatory change for pharmacists – The case for its retention post the pandemic. *Research in Social and Administrative Pharmacy* 2020

85. Erku DA, Belachew SA, Abrha S, et al. When fear and misinformation go viral: Pharmacists' role in deterring medication misinformation during the 'infodemic' surrounding COVID-19. *Research in Social and Administrative Pharmacy* 2020 doi: 10.1016/j.sapharm.2020.04.032

86. Andrawis M, Carmichael J, Collins CD, et al. Improving patient care and demonstrating value during a global pandemic: Recommendations from leaders of the Pharmacy Accountability Measures Work Group. *American journal of health-system pharmacy : AJHP : official journal of the American Society of Health-System Pharmacists* 2020

87. Kawaguchi-Suzuki M, Nagai N, Akonoghrere RO. COVID-19 Pandemic Challenges and Lessons Learned by Pharmacy Educators Around the Globe. *American Journal of Pharmaceutical Education* 2020;84(8) [published Online First: 2020 Aug 1]

88. General Pharmaceutical Council. Rising to the challenge [blog] 2020 [Available from: https://www.pharmacyregulation.org/regulate/blog/rising-challenge accessed 14/01/2020 2020.

89. Robinson J. Government consults on allowing pharmacy technicians to administer COVID-19 and flu vaccines. *The Pharmaceutical Journal* 2020 doi: 10.1211/PJ.2020.20208311

90. Putt W. Pharmacy Technicians Rise to Meet Patient Needs During Coronavirus Pandemic. *Pharmacy Times* 2020

91. Green TC, Bratberg J, Finnell DS. Opioid use disorder and the COVID 19 pandemic: A call to sustain regulatory easements and further expand access to treatment. *Subst Abus* 2020;41(2):147-49.

92. Carico R. Pharmacists continue to protect communities during COVID-19. *Journal of the American Pharmacists Association* 2020;60(5):660-61. doi: 10.1016/j.japh.2020.08.009

93. Bond C, Blenkinsopp A, Inch J, et al. The effect of the new community pharmacy contract on the community pharmacy workforce. *London: Pharmacy Practice Research Trust* 2008;8:9.

For peer review only - http://bmjopen.bmj.com/site/about/guidelines.xhtml

94. Melton BL, Lai Z. Review of community pharmacy services: what is being performed, and where are the opportunities for improvement? *Integr Pharm Res Pract* 2017;6:79-89. doi: 10.2147/IPRP.S107612

95. Wilson CG, Ramage M, Fagan EB. A Primary Care Response to COVID-19 for Patients with an Opioid Use Disorder. *J Rural Health* 2020 doi: 10.1111/jrh.12438

96. Kostev K, Lauterbach S. Panic buying or good adherence? Increased pharmacy purchases of drugs from wholesalers in the last week prior to Covid-19 lockdown. *J Psychiatr Res* 2020;130:19-21.

97. Bell JS, Reynolds L, Freeman C, et al. Strategies to promote access to medications during the COVID-19 pandemic. *Aust J Gen Pract* 2020;49(8):530-32.

98. Romano S, Galante H, Figueira D, et al. Time-trend analysis of medicine sales and shortages during COVID-19 outbreak: Data from community pharmacies. *Research in Social and Administrative Pharmacy* 2020 doi: 10.1016/j.sapharm.2020.05.024

99. Gross AE, MacDougall C. Roles of the clinical pharmacist during the COVID-19 pandemic. *JACCP JAm Coll Clin Pharm* 2020;3(3):564-66.

100. Lapeyre-Mestre M, Boucher A, Daveluy A, et al. Addictovigilance contribution during COVID-19 epidemic and lockdown in France. *Therapies* 2020 doi: http://dx.doi.org/10.1016/j.therap.2020.06.006

101. Alderman C. Pharmacy Services and the Novel Coronavirus. *The Senior Care Pharmacist* 2020;35 (4):146-47 doi: 10.4140/TCP.n.2020.146

102. Hasan SS, Kow CS, Zaidi STR. Social distancing and the use of PPE by community pharmacy personnel: Does evidence support these measures? *Research in Social and Administrative Pharmacy* 2020;17(2):456-9.

103. Thomas CEL, Phipps DL, Ashcroft DM. When procedures meet practice in community pharmacies: qualitative insights from pharmacists and pharmacy support staff. *BMJ open* 2016;6(6):e010851-e51. doi: 10.1136/bmjopen-2015-010851

For peer review only

For peer review only - http://bmjopen.bmj.com/site/about/guidelines.xhtml

104. NHS. Standard operating procedure: COVID-19 local vaccination services deployment in community settings. London: National Health Service 2020.

105. Cabas P, Di Bella S, Giuffrè M, et al. Community pharmacists' exposure to COVID-19. *Research in Social and Administrative Pharmacy* 2021;17(1):1882-87. doi: 10.1016/j.sapharm.2020.05.020

106. Sum ZZ, Ow CJW. Community pharmacy response to infection control during COVID-19. A cross-sectional survey. *Res Soc Adm Pharm* 2020;17(1):1845-52.

107. Al-Quteimat OM, Amer AM. SARS-CoV-2 outbreak: How can pharmacists help? *Research in Social and Administrative Pharmacy* 2021;17(2):480-82. doi: 10.1016/j.sapharm.2020.03.018 [published Online First: 2020 Mar 26]

108. Dawoud D. Emerging from the other end: Key measures for a successful COVID-19 lockdown exit strategy and the potential contribution of pharmacists. *Research in Social and Administrative Pharmacy* 2020 doi: 10.1016/j.sapharm.2020.05.011

109. Weir NM, Newham R, Bennie M. A literature review of human factors and ergonomics within the pharmacy dispensing process. *Research in Social and Administrative Pharmacy* 2020;16(5):637-45. doi: 10.1016/j.sapharm.2019.08.029

110. Maine LL. An Unparalleled Spirit of Collaboration Amid the COVID-19 Pandemic. *Am J Pharm Educ* 2020;84(6):ajpe8155-ajpe55.

111. Dowden A. Will Serious Shortage Protocols help ease drug shortages? *Prescriber* 2020;31(5):29-31. doi: https://doi.org/10.1002/psb.1844

112. NHS England, NHS Improvement. Novel coronavirus (COVID-19) standard operating procedure-Community Pharmacy. Available on: https://www. england. nhs. uk/wp-content/uploads/2020/02/20200305-COVID-19-PRIMARY-CARE-SOP-COMM-PHARM-PUBLICATION-V1. 2020 Apr;1: skills4pharmacy.org.uk 2020.

113. Butt M. Covid-19 vaccination: Logistical challenges for general practice. *Practice Management* 2021;31(1):20-21. doi: 10.12968/prma.2021.31.1.20

For peer review only - http://bmjopen.bmj.com/site/about/guidelines.xhtml

114. National Pharmacy Association (NPA). Pharmacy on the NHS frontline 2020 [Available from: https://nhsfrontline.com/coronavirus-stories-from-the-health-service-frontline/ accessed 17/08/2020 2020.

115. Pharmaceutical Services Negotiating Committee. Community Pharmacy Contractual Framework: Pharmaceutical Services Negotiating Committee; 2021 [Available from: https://psnc.org.uk/contract-it/the-pharmacy-contract/.

116. Quinn B. UK pharmacists facing abuse and violence during lockdown. *The Guardian* 2020 Apr 2nd 2020.

117. Wickware C. Health board pilots delivery of flu vaccines by pharmacy technicians to manage increased demand. *The Pharmaceutical Journal* 2020 doi: 10.1211/PJ.2020.20208565

118. Royal Pharmaceutical Society. The future of pharmacy in a sustainable NHS: Key principles for transformation and growth. London: Royal Pharmaceutical Society, 2020.

119. Sparkman A, Brookhart AL, Goode J-VKR. The impact of an immunization check-up at a pharmacist-provided employee health screening. *Journal of the American Pharmacists Association* 2017;57(3):S274-S78. doi: 10.1016/j.japh.2017.02.010

120. Rodgers RM, Gammie SM, Loo RL, et al. Comparison of pharmacist and public views and experiences of community pharmacy medicines-related services in England. *Patient Prefer Adherence* 2016;10:1749-58. doi: 10.2147/PPA.S112931

121. Hindi AMK, Schafheutle EI, Jacobs S. Patient and public perspectives of community pharmacies in the United Kingdom: A systematic review. *Health Expectations* 2017;21(2):409-28. doi: 10.1111/hex.12639

122. Letley L, Ramsay M. Discussing the MMR vaccine with patients. *The Pharmaceutical Journal* 2019 doi: 10.1211/pj.2019.20205819

123. Kember J, Hodson K, James DH. The public's perception of the role of community pharmacists in Wales. *International Journal of Pharmacy Practice* 2017;26(2):120-28. doi: 10.1111/ijpp.12375

For peer review only

For peer review only - http://bmjopen.bmj.com/site/about/guidelines.xhtml

124. Gidman W, Ward P, McGregor L. Understanding public trust in services provided by community pharmacists relative to those provided by general practitioners: a qualitative study. *BMJ open* 2012;2(3):e000939. doi: 10.1136/bmjopen-2012-000939

125. McConeghy KW, Wing C. A national examination of pharmacy-based immunization statutes and their association with influenza vaccinations and preventive health. *Vaccine* 2016

126. Cheong MWL, Brock T, Karwa R, et al. COVID-19 and Clinical Pharmacy Worldwide - A Wake Up Call and a Call to Action. *JACCP JAm Coll Clin Pharm* 2020;3(5):860-63.

127. Adunlin G, Murphy PZ, Manis M. COVID-19: How Can Rural Community Pharmacies Respond to the Outbreak? *J Rural Health* 2020 doi: 10.1111/jrh.12439

128. Aruru M, Truong HA, Clark S. Pharmacy Emergency Preparedness and Response (PEPR) framework for expanding pharmacy professionals' roles and contributions to emergency preparedness …. *Research in Social and Administrative Pharmacy* 2020;17(1):1967-77.

129. Fiore V. GP referrals under CPCS to go live November 1, NHSE confirms. *Chemist and Druggist* 2020

130. Robinson J. Public Health England to review guidance against the use of rapid COVID-19 tests in community pharmacies. *the Pharmaceutical Journal* 2020;305 doi: 10.1211/PJ.2020.20208550

131. Buchan SA, Rosella LC, Finkelstein M, et al. Impact of pharmacist administration of influenza vaccines on uptake in Canada. *Canadian Medical Association Journal* 2016;189(4):E146-E52. doi: 10.1503/cmaj.151027

132. Calo WA, Shah PD, Gilkey MB, et al. Implementing pharmacy-located HPV vaccination: findings from pilot projects in five U.S. states. *Human Vaccines & Immunotherapeutics* 2019;15(7-8):1831-38. doi: 10.1080/21645515.2019.1602433

133. Anderson C, Thornley T. "It's easier in pharmacy": why some patients prefer to pay for flu jabs rather than use the National Health Service. *BMC Health Services Research* 2014;14(1) doi: 10.1186/1472-6963-14-35

For peer review only

134. Baroy J, Chung D, Frisch R, et al. The impact of pharmacist immunization programs on adult immunization rates: a systematic review and meta-analysis. *Journal of the American Pharmacists Association* 2016;56(4):418-26.

135. Anderson C, Thornley T. Who uses pharmacy for flu vaccinations? Population profiling through a UK pharmacy chain. *International Journal of Clinical Pharmacy* 2016;38(2):218-22. doi: 10.1007/s11096-016-0255-z

136. Pharmacy Guild of Australia. 93 per cent of Aussies recognise benefits of flu shot in pharmacy– Pharmacy Guild of Australia [blog]: Pharmacy Guild of Australia 2017.

137. Latif A, Tariq S, Abbasi N, et al. Giving Voice to the Medically Under-Served: A Qualitative Co-Production Approach to Explore Patient Medicine Experiences and Improve Services to Marginalized Communities. *Pharmacy (Basel, Switzerland)* 2018;6(1):13. doi: 10.3390/pharmacy6010013

138. Anderson C, Blenkinsopp A, Armstrong M. Feedback from community pharmacy users on the contribution of community pharmacy to improving the public's health: a systematic review of the peer reviewed and non-peer reviewed literature 1990-2002. *Health Expect* 2004;7(3):191-202. doi: 10.1111/j.1369-7625.2004.00274.x

139. Eades CE, Ferguson JS, O'Carroll RE. Public health in community pharmacy: a systematic review of pharmacist and consumer views. *BMC public health* 2011;11:582-82. doi: 10.1186/1471-2458-11-582

140. Hassell K, Rogers A, Noyce P. Community pharmacy as a primary health and self-care resource: a framework for understanding pharmacy utilization. *Health and Social Care in the Community* 2000;8(1):40-49. doi: 10.1046/j.1365-2524.2000.00222.x

141. Alkhalili M, Ma J, Grenier S. Defining Roles for Pharmacy Personnel in Disaster Response and Emergency Preparedness. *Disaster Medicine and Public Health Preparedness* 2017;11(4):496-504. doi: 10.1017/dmp.2016.172

For peer review only - http://bmjopen.bmj.com/site/about/guidelines.xhtml

813 142. Ford H, Dallas CE, Harris C. Examining Roles Pharmacists Assume in Disasters: A Content Analytic Approach. *Disaster Medicine and Public Health Preparedness* 2013;7(6):563-72. doi: 10.1017/dmp.2013.99

816 143. Schwerzmann J, Graitcer SB, Jester B, et al. Evaluating the Impact of Pharmacies on Pandemic Influenza Vaccine Administration. *Disaster Medicine and Public Health Preparedness* 2017;11(5):587-93. doi: 10.1017/dmp.2017.1

819 144. Donald M, King-Shier K, Tsuyuki RT, et al. Patient, family physician and community pharmacist perspectives on expanded pharmacy scope of practice: a qualitative study. *CMAJ Open* 2017;5(1):E205-E12. doi: 10.9778/cmajo.20160135

822 145. Mossialos E, Courtin E, Naci H, et al. From “retailers” to health care providers: Transforming the role of community pharmacists in chronic disease management. *Health Policy* 2015;119(5):628-39. doi: 10.1016/j.healthpol.2015.02.007

825 146. Mossialos E, Naci H, Courtin E. Expanding the role of community pharmacists: Policymaking in the absence of policy-relevant evidence? *Health Policy* 2013;111(2):135-48. doi: 10.1016/j.healthpol.2013.04.003

828 147. Ilardo ML, Speciale A. The Community Pharmacist: Perceived Barriers and Patient-Centered Care Communication. *International Journal of Environmental Research and Public Health* 2020;17(2):536. doi: 10.3390/ijerph17020536

831 148. Daly CJ, Quinn B, Mak A, et al. Community Pharmacists’ Perceptions of Patient Care Services within an Enhanced Service Network. *Pharmacy* 2020;8(3):172.

833 149. Ecarnot F, Crepaldi G, Juvin P, et al. Pharmacy-based interventions to increase vaccine uptake: report of a multidisciplinary stakeholders meeting. *BMC Public Health* 2019;19(1) doi: 10.1186/s12889-019-8044-y

836 150. Moullin JC, Sabater-Hernández D, Fernandez-Llimos F, et al. Defining professional pharmacy services in community pharmacy. *Research in Social and Administrative Pharmacy* 2013;9(6):989-95. doi: 10.1016/j.sapharm.2013.02.005

For peer review only - http://bmjopen.bmj.com/site/about/guidelines.xhtml

For peer review only

151. NHS England. General practice forward view: NHS England, 2016.

152. Milosavljevic A, Aspden T, Harrison J. Community pharmacist-led interventions and their impact on patients' medication adherence and other health outcomes: a systematic review. *The International journal of pharmacy practice* 2018;26(5):387-97. doi: 10.1111/ijpp.12462 [published Online First: 2018/06/22]

153. Justwan F, Baumgaertner B, Carlisle JE, et al. The effect of trust and proximity on vaccine propensity. *PLOS One* 2019;14(8):e0220658. doi: 10.1371/journal.pone.0220658

154. Pharmaceutical Services Negotiating Committee. Preparations for a potential COVID-19 vaccination programme.: Pharmaceutical Services Negotiating Committee; 2020 [Available from: https://psnc.org.uk/our-news/preparations-for-a-potential-covid-19-vaccination-programme/ accessed 08/11/2020.

For peer review only

For peer review only - http://bmjopen.bmj.com/site/about/guidelines.xhtml

Appendix 1 – Search terms

Search log – 28th August 2020

Source	Date searched	Search strategy	Hits (or records obtained from searches)
**Publish or Perish (Google Scholar)**	31/07/2020	Pharmacy[titl] AND Covid*	**715**
**Publish or Perish (Google Scholar)**	31/07/2020	Pharmacist[titl] AND Covid*	**225**
**Ovid MEDLINE**	31/07/2020	**exp Coronavirus/ OR exp Coronavirus Infections/ OR (coronavirus* OR 2019-nCoV OR 2019 ncov OR nCov OR Covid-19 OR Covid19 OR SARS-CoV-2 OR novel coronavirus OR novel corona virus OR covid* or coronavirus 2 OR coronavirus infection* OR coronavirus disease OR corona virus disease OR new coronavirus OR new corona virus OR new coronaviruses OR novel coronaviruses OR wuhan) AND (Community pharmacy OR community pharmacies OR community pharmacist OR community pharmacists OR retail pharmacy OR retail pharmacist OR retail pharmacists OR dispensing chemist OR dispensing chemists OR [(Pharmacist OR pharmacy) AND (community OR “primary care”)] OR dispensary OR apothecary OR druggist)**	**61**
**28th August 2020**	EMBASE	**1. exp Coronavirus/ or exp Coronavirus Infections/ or (coronavirus* or "2019-nCoV" or " 2019 ncov" or nCov or "Covid-19" or Covid19 or "SARS-CoV-2" or "novel coronavirus" or "novel corona virus" or covid* or "coronavirus 2" or "coronavirus infection*" or "coronavirus disease" or "corona virus disease" or "new coronavirus" or "new corona virus" or "new coronaviruses" or "novel coronaviruses" or wuhan).mp. [mp=title, abstract, heading word, drug trade name, original title, device manufacturer, drug manufacturer, device trade name, keyword, floating subheading word, candidate term word] 78202**<br>**2. ("Community pharmacy" or "community pharmacies" or "community pharmacist" or "community pharmacists" or "retail pharmacy" or "retail pharmacist" or "retail pharmacists" or "dispensing chemist" or "dispensing chemists" or dispensary or apothecary or druggist).mp. [mp=title, abstract, heading word, drug trade name, original title, device manufacturer, drug**	**52 hits**

For peer review only - http://bmjopen.bmj.com/site/about/guidelines.xhtml

		manufacturer, device trade name, keyword, floating subheading word, candidate term word] 15896<br>3. ((Pharmacist or pharmacy or pharmacies) and (community or "primary care")).mp. [mp=title, abstract, heading word, drug trade name, original title, device manufacturer, drug manufacturer, device trade name, keyword, floating subheading word, candidate term word] 26928<br>4. 2 or 3 29215<br>5. 1 and 4 89<br>6. limit 5 to medline 37<br>7. 5 not 6	
**28th August 2020**	CINAHL	**((((("Community pharmacy" or "community pharmacies" or "community pharmacist" or "community pharmacists" or "retail pharmacy" or "retail pharmacist" or "retail pharmacists" or "dispensing chemist" or "dispensing chemists" or dispensary or apothecary or druggist)) OR (((Pharmacist or pharmacy or pharmacies) and (community or "primary care")))) AND exp Coronavirus/ or exp Coronavirus Infections/ or (coronavirus* or "2019-nCoV" or " 2019 ncov" or nCov or "Covid-19" or Covid19 or "SARS-CoV-2" or "novel coronavirus" or "novel corona virus" or covid* or "coronavirus 2" or "coronavirus infection*" or "coronavirus disease" or "corona virus disease" or "new coronavirus" or "new corona virus" or "new coronaviruses" or "novel coronaviruses" or wuhan)**	**24 hits**
**28th August 2020**	Web of Science	**# 4 14 #1 AND #2**<br>**Refined by: [excluding] Databases: (MEDLINE)**<br>**Databases= WOS, BCI, BIOSIS, CCC, DRCI, DIIDW, KJD, MEDLINE, RSCI, SCIELO, ZOOREC Timespan=All years**<br>**# 3 102 #1 AND #2**<br>**Databases= WOS, BCI, BIOSIS, CCC, DRCI, DIIDW, KJD, MEDLINE, RSCI, SCIELO, ZOOREC Timespan=All years**<br>**# 2 32,714 TS=((("Community pharmacy" or "community pharmacies" or "community pharmacist" or "community pharmacists" or "retail pharmacy" or "retail pharmacist" or "retail pharmacists" or "dispensing chemist" or "dispensing chemists" or dispensary or apothecary or druggist)) OR (((Pharmacist or pharmacy or pharmacies) and (community or "primary care"))))**<br>**Databases= WOS, BCI, BIOSIS, CCC, DRCI, DIIDW, KJD, MEDLINE, RSCI, SCIELO, ZOOREC Timespan=All years**	**14 hits**

For peer review only - http://bmjopen.bmj.com/site/about/guidelines.xhtml

		# 1 95,896 TS=(coronavirus* or "2019-nCoV" or " 2019 ncov" or nCov or "Covid-19" or Covid19 or "SARS-CoV-2" or "novel coronavirus" or "novel corona virus" or covid* or "coronavirus 2" or "coronavirus infection*" or "coronavirus disease" or "corona virus disease" or "new coronavirus" or "new corona virus" or "new coronaviruses" or "novel coronaviruses" or wuhan)<br><br>Databases= WOS, BCI, BIOSIS, CCC, DRCI, DIIDW, KJD, MEDLINE, RSCI, SCIELO, ZOOREC Timespan=All years	
28th August 2020	Scopus	(TITLE-ABS-KEY (coronavirus* OR "2019-nCoV" OR " 2019 ncov" OR ncov OR "Covid-19" OR covid19 OR "SARS-CoV-2" OR "novel coronavirus" OR "novel corona virus" OR covid* OR "coronavirus 2" OR "coronavirus infection*" OR "coronavirus disease")) AND (((("Community pharmacy" OR "community pharmacies" OR "community pharmacist" OR "community pharmacists" OR "retail pharmacy" OR "retail pharmacist" OR "retail pharmacists" OR "dispensing chemist" OR "dispensing chemists" OR dispensary OR apothecary OR druggist)) OR (((pharmacist OR pharmacy OR pharmacies) AND (community OR "primary care")))))	591 hits
28th August 2020		Search of the contents of preprint services and the WHO Covid register: pharmacy OR pharmacist OR pharmacists or pharmacies	(402 hits)
28th August 2020		e.g. the curated BioRxiv/MedRxiv dataset (connect.medrxiv.org/relate/content/181).	10 hits and 19 hits – none relevant
28th August 2020		Social media (e.g. blogs) including facilitated twitter® discussion (#Cpharmchat) on the Impact of COVID-19 on community pharmacy.	Twitter 8 hits
28th August 2020		Web-sites/emails from relevant regulators and professional organisations including RPS (https://www.rpharms.com/), PDA (https://www.the-pda.org/), PSNC (http://psnc.org.uk/), and GPhC (https://www.pharmacyregulation.org/).	Completed
28th August 2020		Pharmaceutical Journal web-site https://www.pharmaceutical-journal.com/. No language restrictions at title and abstract stage selection stage. Separated high and low/middle income countries at sift stage.	Completed
28th August 2020		Local Pharmaceutical Committees (LPC)	Completed

For peer review only

Appendix 2 - Literature inclusion criteria

Published after January 2003 AND

COVID-19 or other pandemic or other infectious diseases

OR vaccination programmes

OR expanded/extended roles

AND Community Pharmacy

AND High- or middle-income country

For peer review only

PRISMA 2009 Flow Diagram

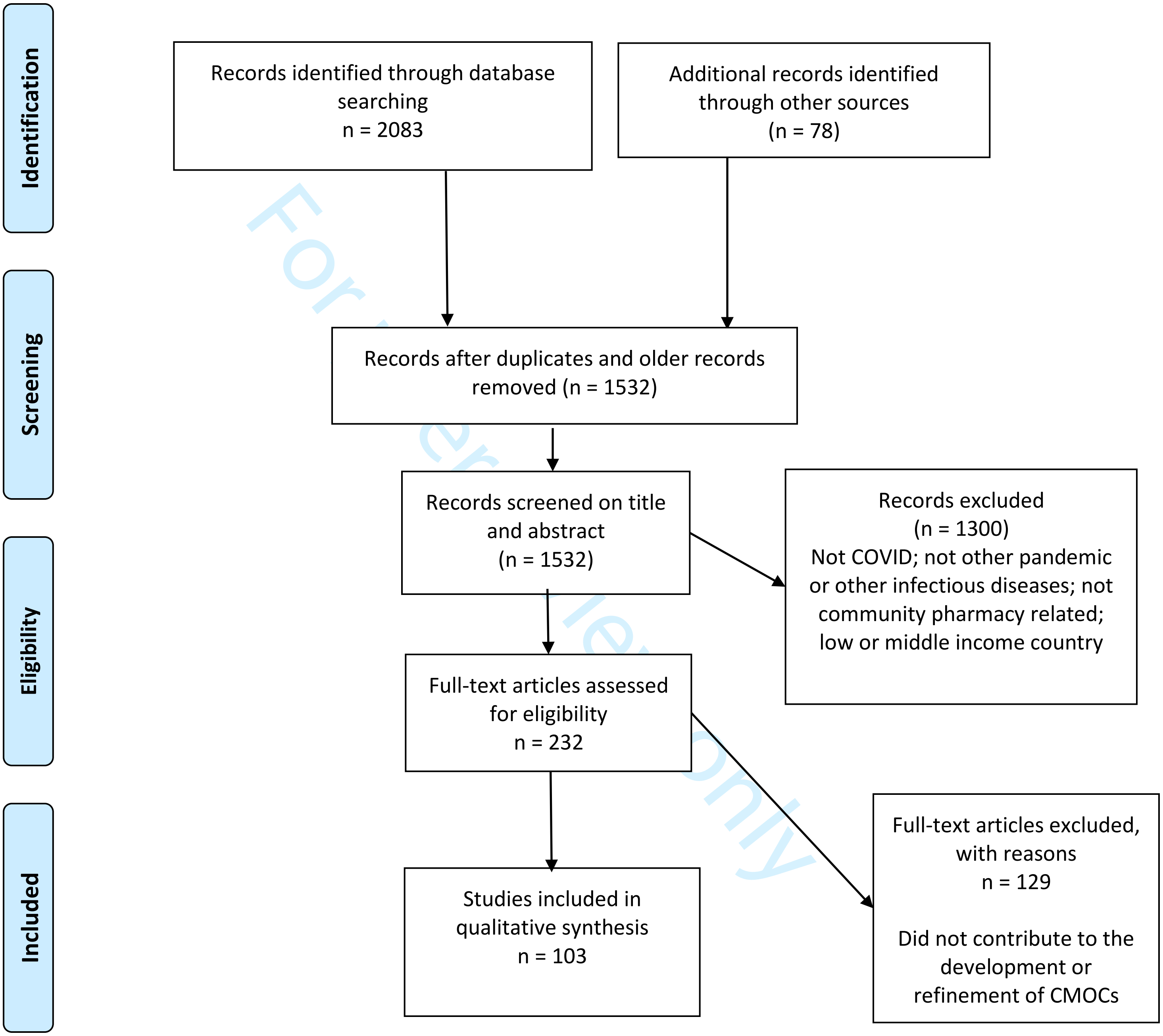

From: Moher D, Liberati A, Tetzlaff J, Altman DG, The PRISMA Group (2009). *P*referred *R*eporting *I*tems for *S*ystematic Reviews and *M*eta-*A*nalyses: The PRISMA Statement. PLoS Med 6(7): e1000097. doi:10.1371/journal.pmed1000097

For more information, visit www.prisma-statement.org.

For peer review only - http://bmjopen.bmj.com/site/about/guidelines.xhtml

Appendix 4 - A simplified diagram of the programme theory of a COVID-19 vaccination programme provided by community pharmacies

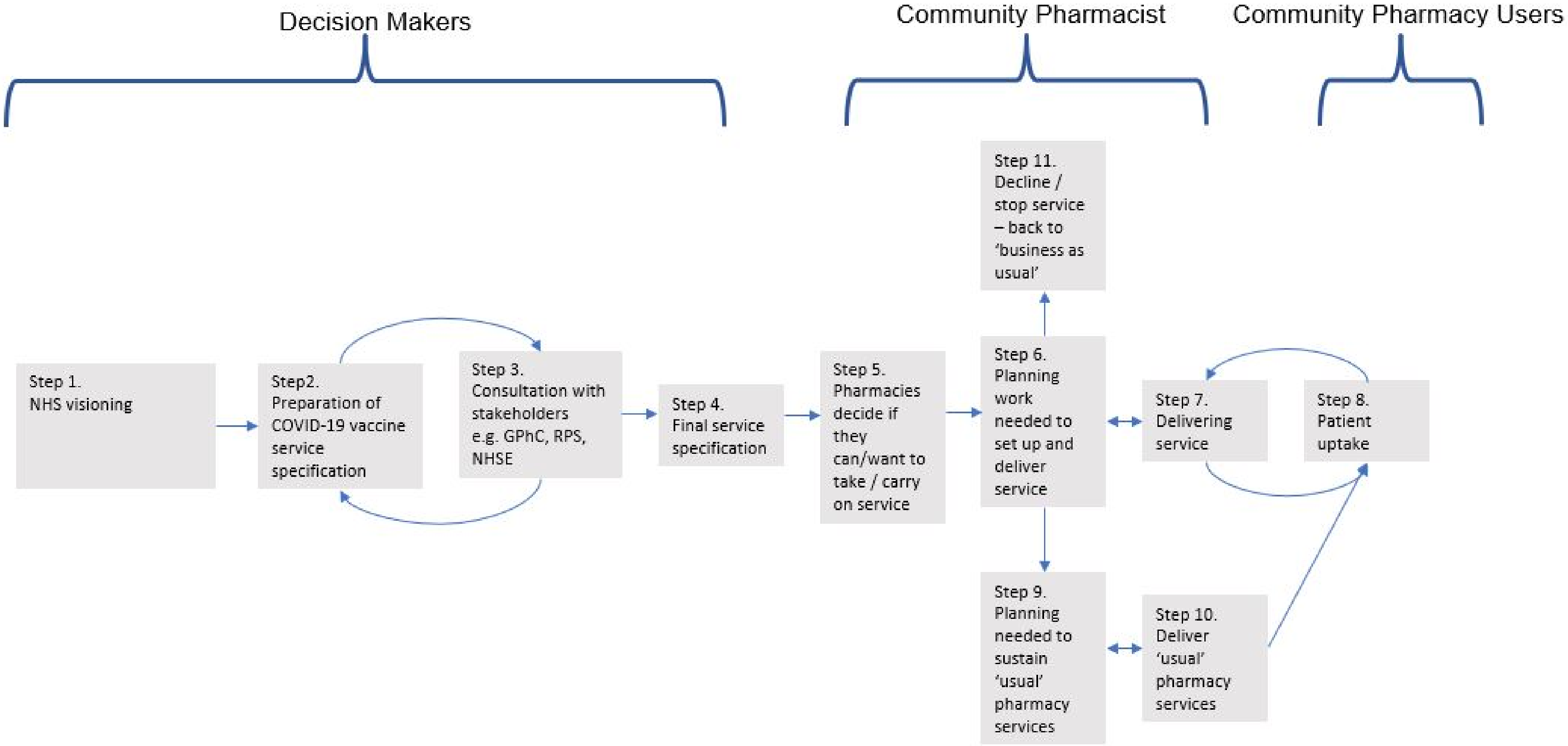

BMJ Open

A Rapid Realist Review of the Role of Community Pharmacy in the Public Health Response to COVID-19

Journal:	*BMJ Open*
Manuscript ID	bmjopen-2021-050043.R1
Article Type:	Original research
Date Submitted by the Author:	24-Mar-2021

Complete List of Authors:	Maidment, Ian; Aston University<br>Young, Emma; The University of Sheffield<br>MacPhee, Maura; The University of British Columbia<br>Booth, Andrew; The University of Sheffield, School of Health & Related Research (ScHARR)<br>Zaman, Hadar; University of Bradford<br>Breen, Juanita; University of Tasmania<br>Hilton, Andrea; University of Hull, FHSC<br>Kelly, Tony; Aston University, PPI Lead on Project<br>Wong, Geoff; University of Oxford, Primary Care Health Sciences

**Primary Subject Heading**:	Health services research
Secondary Subject Heading:	General practice / Family practice, Infectious diseases
Keywords:	COVID-19, QUALITATIVE RESEARCH, Organisation of health services < HEALTH SERVICES ADMINISTRATION & MANAGEMENT

SCHOLARONE™ Manuscripts

I, the Submitting Author has the right to grant and does grant on behalf of all authors of the Work (as defined in the below author licence), an exclusive licence and/or a non-exclusive licence for contributions from authors who are: i) UK Crown employees; ii) where BMJ has agreed a CC-BY licence shall apply, and/or iii) in accordance with the terms applicable for US Federal Government officers or employees acting as part of their official duties; on a worldwide, perpetual, irrevocable, royalty-free basis to BMJ Publishing Group Ltd ("BMJ") its licensees and where the relevant Journal is co-owned by BMJ to the co-owners of the Journal, to publish the Work in this journal and any other BMJ products and to exploit all rights, as set out in our licence.

The Submitting Author accepts and understands that any supply made under these terms is made by BMJ to the Submitting Author unless you are acting as an employee on behalf of your employer or a postgraduate student of an affiliated institution which is paying any applicable article publishing charge ("APC") for Open Access articles. Where the Submitting Author wishes to make the Work available on an Open Access basis (and intends to pay the relevant APC), the terms of reuse of such Open Access shall be governed by a Creative Commons licence – details of these licences and which Creative Commons licence will apply to this Work are set out in our licence referred to above.

Other than as permitted in any relevant BMJ Author's Self Archiving Policies, I confirm this Work has not been accepted for publication elsewhere, is not being considered for publication elsewhere and does not duplicate material already published. I confirm all authors consent to publication of this Work and authorise the granting of this licence.

For peer review only

A Rapid Realist Review of the Role of Community Pharmacy in the Public Health Response to COVID-19

Ian Maidment (corresponding author), Aston University, Birmingham, B4 7ET, UK, i.maidment@aston.ac.uk, Phone/fax: 0121-204-3000; Emma Young, University of Sheffield, Sheffield, UK; Maura MacPhee, University of British Columbia, Vancouver, Canada; Andrew Booth, University of Sheffield, Sheffield, UK; Hadar Zaman, University of Bradford, Bradford, UK; Juanita Breen, University of Tasmania, Hobart; Andrea Hilton, University of Hull, Hull, UK; Tony Kelly, PPI Lead (Aston University), Birmingham, UK; Geoffrey Wong, University of Oxford, Oxford, UK.

Keywords: COVID-19, SARS-CoV-2, Community Pharmacy Services, Vaccination

Word count: 3,744

For peer review only

Abstract

Introduction

Community pharmacists and their teams have remained accessible to the public providing essential services despite immense pressures during the COVID-19 pandemic. They have successfully expanded the influenza vaccination programme and are now supporting the delivery of the COVID-19 vaccination roll-out.

Aim

This rapid realist review aims to understand how community pharmacy can most effectively deliver essential and advanced services, with a focus on vaccination, during the pandemic and in the future.

Method

An embryonic programme theory was generated using four diverse and complementary documents along with the expertise of the project team. Academic databases, preprint services and grey literature were searched and screened for documents meeting our inclusion criteria. The data was extracted from 103 documents to develop and refine a programme theory using a realist logic of analysis. Our analysis generated 13 context-mechanism-outcome configurations explaining when, why and how community pharmacy can support public health vaccination campaigns, maintain essential services during pandemics, and capitalise on opportunities for expanded, sustainable public health service roles. The views of stakeholders including pharmacy users, pharmacists, pharmacy teams and other healthcare professionals were sought throughout to refine the 13 explanatory configurations.

Results

The 13 context-mechanism-outcome configurations are organised according to decision makers, community pharmacy teams and community pharmacy users as key actors. Review findings include:

For peer review only

supporting a clear role for community pharmacies in public health; clarifying pharmacists' legal and professional liabilities; involving pharmacy teams in service specification design; providing suitable guidance, adequate compensation and resources; and leveraging accessible, convenient locations of community pharmacy.

Discussion

Community pharmacy has been able to offer key services during the pandemic. Decision makers must endorse, articulate and support a clear public health role for community pharmacy. We provide key recommendations for decision makers to optimise such a role during these unprecedented times and in the future.

Strengths and limitations of this study

A diverse group of professional and public stakeholders validated our findings from the literature.

By using a realist approach, we were to use broad range of data, including grey literature.

To enable us to develop recommendations in a timely manner the focus of the review was deliberately narrow.

The topic could have been informed by other sources of evidence in particular empirical interviews with key stakeholder.

COVID-19 vaccination is a rapidly evolving area and this research was based on the best available evidence at the time.

For peer review only - http://bmjopen.bmj.com/site/about/guidelines.xhtml

For peer review only

For peer review only - http://bmjopen.bmj.com/site/about/guidelines.xhtml

Introduction

Community pharmacy teams have continued to provide essential services during the coronavirus disease 2019 (COVID-19) pandemic. They offer accessibility and medicines expertise to the public, even in challenging times.[1 2 3] However, COVID-19 creates extra workload demands, such as medication dispensing with increases of up to 33% in prescription numbers.[4] To cope with this demand community pharmacies have increased their opening hours and hired additional staff.[4] Alongside this additional workload, they have managed widening coverage of the influenza vaccines programme.[5]

Evidence suggests that community pharmacy can successfully provide diverse vaccination services including seasonal and pandemic influenza, travel vaccinations and hepatitis B for at-risk groups, within the provisions of the UK National Health Service (NHS) or privately.[6] They have successfully provided influenza vaccines as an NHS commissioned advanced service since 2015.[5] One service evaluation found that of 485 patients asked, 99% expressed confidence in their pharmacist to provide additional vaccinations.[7] Community pharmacy can also support influenza and other vaccinations to combat the significantly higher COVID-19 related mortality in the non-white community.[8 9]

The COVID-19 pandemic has stretched NHS capacity to safely and efficiently meet public health demands. A role for community pharmacy in the national vaccination service requires an understanding of what pharmacy teams require to successfully deliver essential and advanced services during the pandemic. Such knowledge is timely, given the roll-out of COVID-19 vaccines across the community.[10]

Delivering a vaccine is a complex process and successful delivery is context-dependent. A realist review helps make sense of complex situations[11 12], such as how community pharmacy can most

For peer review only - http://bmjopen.bmj.com/site/about/guidelines.xhtml

effectively address the challenges presented by COVID-19. A rapid review can generate guidance for decision makers to assist with roll-out of COVID-19 vaccinations to community pharmacy. This rapid realist review aimed to understand how community pharmacy can most effectively deliver essential and advanced services, with a focus on vaccination, during the pandemic and in the future.

Methods

A rapid realist review of academic and other literature, supplemented by input from key actors, was undertaken to understand how and when community pharmacy can effectively support the public health agenda during pandemics such as COVID-19. Rapid reviews aim to ensure findings are generated and disseminated in response to the urgent nature of the situation. To produce this knowledge at pace, we truncated the following review processes:

- Programme theory development was undertaken within 1 month with input from the project team.
- Searching was expedited using broad search terms and using a limited number of key data sources.
- Data analysis and context-mechanism-outcome configuration (CMOC) development focussed on where the programme theory was considered most important during COVID-19.

This realist review was undertaken within six-months (August 2020 - January 2021), the protocol was published on PROSPERO[13] and, where relevant, follows the RAMESES quality and publication standards.[14]

Stage 1: Programme Theory Development

The project team met virtually to develop an embryonic programme theory using four diverse documents from an initial search representing a professional journal[15], a research journal[16], a policy document (Royal Pharmaceutical Society)[17] and a practical influenza briefing.[18] The team identified the need for (i) enabling guidance for community pharmacy (to achieve legitimisation)[16]; (ii) practical

direction for community pharmacy practices (to ensure feasibility)[15 16]; and (iii) user assurance of appropriate, safe, feasible and timely intervention (relative advantage).[15] The resultant embryonic theory, patterned on a COM-B behavioural model of capability, opportunity, and motivation leading to behaviour[19], was used to inform searching and initial analyses.

Stage 2: Literature Searching

Searches were conducted (July-August 2020) using MEDLINE, EMBASE, CINAHL, Web of Science, and Scopus for search concepts relating to Pharmacy and COVID by AB (see Appendix 1 for search strategy). Reference checking and citation searching of all included references on Google Scholar (using the Publish or Perish tool) were also carried out. Given the novelty of the virus, we searched the contents of preprint services and the World Health Organisation (WHO) COVID Register. Grey literature searches included social media (e.g. blogs, facilitated Twitter® discussion [#Cpharmchat]), community pharmacy websites, and emails from relevant regulators and professional organisations (e.g., Royal Pharmaceutical Society [RPS], Pharmaceutical Services Negotiation Committee [PSNC], General Pharmaceutical Council [GPhC]).

Key inclusion criteria were high or middle-income countries, community pharmacy and infectious disease management (see Appendix 2). The search covered January 2003-July 2020 to include SARS, a comparable condition first identified in 2003. There were no restrictions on study designs eligible for inclusion.

Stage 3: Data Selection and Extraction (selection and coding)

Selection and appraisal of documents followed a two-step procedure:

1). EY screened the title, abstract and keywords of potentially relevant documents against inclusion criteria. A 10% random sample was checked by two research team members (AB and MM) for consistency.

For peer review only

2). EY obtained and screened full texts of all documents meeting the eligibility criteria.

Relevant data from the included full text documents was coded into NVivo by EY, MM and JB. Some codes came from the data (i.e. inductive coding); others were derived from the programme theory (i.e. deductive coding) and some were derived using retroduction (i.e. by interpretation of what might be functioning as mechanisms).[20] No assessment was made of the rigour of the data within included documents, however global judgements were made of the quality of the explanations provided by the CMOCs and programme theory using the criteria of consilience, simplicity and analogy.[21]

Stage 4: Data Synthesis

The data analysis/synthesis was conducted by EY, MM and JB with input from the rest of the project team to develop and refine the programme theory using a realist logic of analysis. Our analysis generated 13 realist CMOCs, explaining when, why and how community pharmacy can support public health vaccination campaigns, maintain essential services during pandemics, and capitalise on opportunities for expanded, sustainable public health service roles. Actor conversations generated further CMOCs related to care for diverse and vulnerable populations, including the Black and Minority Ethnic (BAME) community.

Our realist logic of analysis centred on the following questions:

- Interpretation of meaning: Do the contents coded by the team provide data that may be interpreted as CMOCs?
- Interpretations and judgements about CMOCs: How do the CMOCs relate to the programme theory?
- Interpretations and judgements about programme theory: How do the programme theory and its CMOCs correspond with key actor perspectives of reality?

For peer review only - http://bmjopen.bmj.com/site/about/guidelines.xhtml

160 Data to answer our questions was iteratively sought across documents. Interpretive cross-case comparison was used to identify and to explain the “success” of pandemic community pharmacy interventions delivered in different settings or to different population groups.

Key Stakeholders

Key stakeholders, including community pharmacists and support staff (including representatives from large and smaller chains, sole independent pharmacies and primary care), other healthcare professionals and members of the public were consulted on four occasions. The meetings took place over Microsoft Teams and each lasted about one hour.

Patient and Public Involvement

Members of the public were drawn from The University of Sheffield’s Patient and Public Involvement database and contacts of the core project team. Groups numbered between 11 and 13 members, with ages ranging from 22 to 74 years, from diverse locations and ethnicities including Black African, Black Caribbean, British Asian, British Chinese, White Irish and White British. Collectively, stakeholders provided feedback and advice on their real-world experience of working in or using community pharmacy.

Results

103 documents were included in this rapid review and were coded to develop and refine our CMOCs and programme theory (PRISMA diagram in Appendix 3). The final programme theory is summarised in Appendix 4—from abstract visioning to actual patient uptake of the COVID-19 vaccine. Although the programme theory is outlined in a linear fashion, steps within it are not necessarily linear and may occur simultaneously. The CMOCs are organized according to key actors, or individuals and groups with a vested interest in community pharmacy delivery of a COVID-19 vaccination

For peer review only - http://bmjopen.bmj.com/site/about/guidelines.xhtml

programme. "Actor" derives from sociology and is synonymous with "stakeholder"; we privileged this term to differentiate programme theory/CMOC organization from project stakeholder participants.[22] Table 1 briefly describes the three actor groups with their respective CMOCs and corresponding steps in Appendix 4. Tables 2-4 show the final 13 CMOCs.

Table 1: Programme Theory Actors with corresponding CMOCs and steps

Programme Theory Actors	Description	CMOCs*	Steps in Programme Theory**
Decision Makers	The UK government, regulatory and professional bodies, the public.	CMOC 1 - 4	Steps 1 - 4
Community Pharmacists and their Teams	Community pharmacists are health care professionals registered by the General Pharmaceutical Council and supported by teams made up of counter assistants, dispensers and registered technicians. They work in high street locations, in local communities and in supermarkets. Employers range from large chains to small individually owned community pharmacies.	CMOC 5 – 9	Steps 5 – 7 and 9 – 11
Pharmacy Users	Members of the public who use any community pharmacy services including prescription dispensing, minor ailment advice/treatment or vaccination services.	CMOC 10 - 13	Step 8

*see Table 2-4

**See Appendix 4

For peer review only - http://bmjopen.bmj.com/site/about/guidelines.xhtml

The following sections summarise the CMOCs related to each of the three actor groups. Illustrative examples of the supporting evidence from review documents are presented (Tables 2-4).

Decision makers

Decision maker and public endorsement (CMOC 1) are essential first steps in enlisting community pharmacy for COVID-19 vaccination programmes. Regulators must ensure pharmacists have the legal scope to do so (CMOC 2), with community pharmacy input during the development of policies and protocols (CMOC 3), so that final service specifications are flexible and do-able within local settings (CMOC 4).

Table 2 - Decision Makers (CMOCs 1-4)

**CMOC 1 – Support a public health role**[23 24 25 26-32]	**When the government, pharmacy regulators, professional bodies and the public endorse and support a clear role for community pharmacy in public health services (C), community pharmacists will be more likely to adopt vaccination services (O) because they see it as their professional role and duty (M).**
	"Distribution and administration of the COVID-19 vaccination programme will require concerted action across the NHS. With unique insight and expertise in medicines and the delivery of vaccination programmes, pharmacists have a clear role in contributing to the success of this programme." (Great Britain)[24]<br><br>*"55% of the public have visited a pharmacy during the COVID19 crisis…89% of people believe pharmacies are playing an essential role in the COVID19 crisis." (UK National Pharmacy Association)*[23]

For peer review only - http://bmjopen.bmj.com/site/about/guidelines.xhtml

	"Pharmacists…have been called on to coordinate the administration of COVID-19 tests...providing ongoing COVID-19 surveillance to communities by allowing walk-in testing at community pharmacies…[This] might be more sustainable and convenient than the large-scale public screening being done as of the summer of 2020." (USA)[25]<br><br>*"Given the past success of community pharmacists with increasing annual seasonal influenza uptake and their accessibility, pharmacists will need to be central in administering COVID-19 vaccines in order to achieve rapid population-wide coverage." (Canada)*[26]
**CMOC 2 –Clarify legal and professional liabilities**[24 31 33-40]	**When pharmacy regulators and the NHS clarify community pharmacists' legal and professional liabilities arising from the administration of a novel and potentially unlicensed COVID-19 vaccine (C), community pharmacists are more willing to give the vaccination (O) because they feel reassured regarding liability (M).**
	"The role of pharmacists in the COVID-19 vaccination programme must be made clear to the pharmacy profession itself. Professional and representative pharmacy bodies have an important role to play in providing the right level of information to the profession to support their roles in the vaccination programme." (Great Britain)[24]<br><br>*"Indemnity insurance for individual healthcare professionals needs to be amended to cover this activity and be state-funded. There also needs to be clear communication to healthcare professionals, so they clearly understand*

For peer review only

	that they are covered and under which circumstances this applies." (Great Britain)[41]
**CMOC 3 – Co-develop feasible service specifications**[1 38 42-48]	**When COVID-19 vaccination policy and service specifications have been developed with input from diverse community pharmacists and staff tasked with administering and supporting the administration of the vaccine (C), community pharmacies are more likely to deliver the service (O) because they believe the service specification is feasible (M).**
	"Pharmacists ideally want input into future policy changes before they are finalized, so that these can reflect capacity and preparedness on the ground and be publicized accurately." (Great Britain)[1]
**CMOC 4 – Issue clear, relevant and timely guidance**[25 27 31 34 48-62]	**When government, pharmacy regulators and professional bodies provide consistent, clear, relevant, and timely guidance for the delivery of the COVID-19 vaccines (C), community pharmacies are more likely to deliver the service (O), because the guidance is helpful and simplifies implementation (M).**
	"… it is good to see that NHSE/I have provided the information we have been waiting for to review the resources we have nationwide and decide how we can bring them to bear to help the NHS defeat this virus." (Great Britain)[49]

In England community pharmacies have government contracts and partnerships to deliver vaccinations and other essential services during emergencies, including the COVID-19 pandemic.[25 31 32] Pharmacies in the USA and Canada have also been identified as having a substantive role in vaccine administration (CMOC1).[25,26] The idea of harnessing UK community pharmacy capacity enjoys widespread public support (CMOC2).[23] However, appropriate service delivery is hampered by unfeasible operational conditions (CMOC3)[1]; for example, medication deliveries are funded by the UK government for "vulnerable people", but this category of service users is defined narrowly by the

For peer review only - http://bmjopen.bmj.com/site/about/guidelines.xhtml

government and misunderstood by the public, creating unrealistic expectations of community pharmacy and generating additional work.[1]

In contrast, when clear options for community pharmacy involvement in COVID-19 vaccination programmes were issued through the NHS (CMOC4), the chief executive of the Association of Independent Multiple Pharmacies commented positively on members' engagement in delivering vaccines.[49]

Community Pharmacists and Team

Community pharmacies have had to manage ongoing essential services, in addition to supporting COVID vaccination delivery during the pandemic (CMOC6), including delivery of necessary medications (CMOC7). Given fears and anxieties related to COVID-19 and changes to service delivery, community pharmacies have had to deal with inappropriate behaviours from the public, including emotional abuse and threats of physical abuse (CMOC8). As an integral public health service, community pharmacy capacity to meet NHS needs will be enhanced through use of IT and collaboration with other service providers (CMOC9).

Table 3 - Community Pharmacists and Team (CMOCs 5-9)

**CMOC 5 – Receive adequate compensation and resources**[1 63-77]	**When community pharmacies receive adequate compensation and resource support for COVID-19 vaccines (C), they are more likely to deliver the service (O), because they feel recognised for their service contributions (M).**
	"Pharmacists…have been incredible in supporting patients throughout COVID-19 and rightly deserve recognition for the work they do. We know that teams were already under pressure and that colleagues in community pharmacy are feeling added financial strain." (Great Britain).[63]

	"In the community, mitigating the impact of COVID-19 has…focused largely on general practitioners (GPs). This is unsurprising to those in the pharmacy profession who have long considered policy makers to overlook them." (Great Britain)[1]
**CMOC 6 – Sustain capacity and facility to adapt essential services**[16 17 30 34 68 70 71 78-91 92]	**When a community pharmacy has the capacity and permission to manage and adapt existing essential services during COVID-19 (C), they are more likely to effectively deliver and sustain these services (O) because it's feasible for them to do so (M).**
	"Virtual and telephone consultations have become commonplace, particularly to vulnerable patients. Pharmacists have implemented systems to dispense medications in advance of need to minimise wait times and duplicate visits. In case-by-case examples…there has been anticipatory management of medication-related needs." (Ireland)[92]<br><br>*"Pharmacists who had comfort and confidence in managing electronic communication reported feeling greater control over workflow and the ability to triage and queue patients more effectively based on priority and need." (Canada)*[60]
**CMOC 7 – Inform users of essential service availability/continuity**	**When community pharmacies have processes in place to inform pharmacy users about the availability of essential services (C), pharmacy users are less likely to become anxious (O), because they feel reassured about access to what they need (M).**

For peer review only - http://bmjopen.bmj.com/site/about/guidelines.xhtml

92 16 28 31 32 51 60 65 71 80 81 85 93-101	*"Access to medications is a main concern expressed by our patients… The CDC and SAMHSA offer guidance on ways to reduce stress and anxiety in this time of uncertainty."*[95]<br><br>*"As a result of the power dynamics at play, it is ultimately up to pharmacists to be able to reassure patients and provide care, all while taking into account their mental health. Currently, guidelines regarding patient interaction during a pandemic are needed".*[51]<br><br>*"In addition, there is ongoing work in liaison with pharmacists, general practitioners, and state and territory authorities to enable therapeutic substitution by pharmacists in the event of a shortage. This will allow community pharmacists to substitute dose strength or form without prior approval from the prescriber, if a prescribed medicine is not available at the time of dispensing. These measures highlight the important role pharmacists can play in enabling and maintaining access to medicines for people in need throughout the COVID-19 outbreak." (Australia)*[85]<br><br>*"Access to medications is a main concern expressed by our patients… The CDC and SAMHSA offer guidance on ways to reduce stress and anxiety in this time of uncertainty." (USA)*[95]
**CMOC 8 – Protect the health and safety of staff** [24 31 51 71 77 81 102-108]	**When community pharmacies have the means to protect the health of pharmacists and staff (C), they are more likely to deliver essential services and COVID-19 vaccination services (O), because they feel safe to do so (M).**

For peer review only - http://bmjopen.bmj.com/site/about/guidelines.xhtml

	"…Given high levels of public anxiety and uncertainty regarding the integrity of the drug supply chain in Canada, many participants reported difficult, sometimes frightening, interactions with members of the public and their desire for dedicated security to provide support and conflict management" (Canada).[31]<br><br>*"An additional burden Asian pharmacists face, on top of pharmacist harassment, is the rise of anti-Asian racism that has come about due to COVID-19. Verbal and even physical abuse has been reported to happen in various countries, such as the UK, France, and the USA, to those of Chinese descent…" (UK, France, USA)*[51]<br><br>*"The survey found that although more than a third (37%) of pharmacists said they felt unsafe at some point working during the pandemic, concerns over PPE have eased. This is due, in part, to the supply problems being eased and community pharmacists being finally allowed to order from the national online PPE portal in an emergency, after calls for access from the RPS." (Great Britain)*[77]
**CMOC 9 – Enhance collaboration across services, including IT**[18 26 47 56 57 69 78 80 85 109-114]	**When systems needed for COVID-19 vaccination, including IT and remuneration, support collaboration by various service providers (e.g. general practitioner surgeries and community pharmacies: (C), a coordinated response is more likely (O), because reduced effort is needed from everyone (M).**

For peer review only - http://bmjopen.bmj.com/site/about/guidelines.xhtml

	"Planning and delivery should be undertaken across a consistent, pre-agreed footprint. It may be more efficient and cost effective to provide immunisation across a number of providers, pooling resources and sites to deliver the best service possible, and working in coordination with other local stakeholders such as directors of public health and local government." (Great Britain)[47]<br><br>*"For many community pharmacists, a lack of connected IT is a huge problem. Kieran Eason, who runs an independent pharmacy in Tamworth, Staffordshire, says lack of intra-operability makes it more difficult to do relatively simple things, like sending prescription requests to GPs. "Pharmacy IT is just a complete disaster," he says, suggesting the COVID-19 crisis has highlighted flaws, such as the number of different systems pharmacists use." (Great Britain)*[69]

Although attention is focused on a COVID-19 vaccine, community pharmacies offer diverse essential services. In the UK and other high-income countries, community pharmacy services also provide advanced services such as vaccinations.[115] Pharmacists consider it a professional responsibility to provide essential services during the pandemic, despite clear financial risks to themselves.[63]

Despite pharmacists' professional and moral obligations to provide essential services, ongoing persistent 'under-recognition' can jeopardize their ability to contribute to COVID-19 vaccinations (CMOC5) as well as maintain a usual service. Under-recognition has been an issue in previous UK vaccination campaigns; respondents to a survey of Welsh community pharmacists after the 2016 influenza season described providing a "mop up" service for general practitioners (GPs).[59 68]

For peer review only

For peer review only - http://bmjopen.bmj.com/site/about/guidelines.xhtml

Multiple required community pharmacy service adaptations have been reported by the UK Pharmaceutical Journal, including changeover in retail space to medication preparation and dispensing, and call-in shopping services for other retail items (CMOC6). Service adaptations have been affected by hours of operation, available staff, and cancellations of contracted services, such as blood pressure testing and smoking cessation support—all with financial implications for pharmacists.[80]

Before COVID-19, community pharmacies globally were offering "valued-added services" (VAS) such as drive-thru services, online ordering and communications services (e.g., prescription reminders) to stay competitive. Many VAS services have helped pharmacies adapt more quickly to pandemic restrictions.[81]

During the pandemic, ensuring access to needed medications has been a critical pharmacy service, to allay public concerns (CMOC7).[16] Potential and actual disruptions in expected services and needed supplies (e.g., medications) have resulted in tensions, threats and verbal/physical abuse by the public to community pharmacists (CMOC 8).[51 80 116] Safety policies, protocols and safety-related supplies (e.g., PPEs) must in place to ensure community pharmacy teams' safety.[80]

The UK COVID-19 vaccination campaign borrows heavily from previous, successful collaborative influenza vaccination programmes using community pharmacy and GPs (CMOC9).[48] Pooling resources improves service delivery.[117] For example, the UK Pharmaceutical Journal reported how a greater collaborative approach has resulted in successful influenza vaccination of home care staff and domiciliary workers during the pandemic.[117]

The pandemic has clearly demonstrated the importance of interoperable, connected IT systems across services.[118] Getting access to reliable information is important for tracking supplies and deliveries related to the COVID-19 vaccination programme.[69] A pandemic silver lining is raised awareness of IT functions for enhanced delivery of essential services (e.g., medication planning,

For peer review only - http://bmjopen.bmj.com/site/about/guidelines.xhtml

prescribing and dispensing between pharmacies and GPs) and advanced services, including vaccinations.[69]

Pharmacy Users

Pharmacy users trust community pharmacies as a reliable source of information (CMOC10) about vaccines, and pharmacies' local accessibility and convenience increases the likelihood of users obtaining COVID-19 vaccines through them (CMOC11). Community pharmacy relationships with vulnerable populations in their local settings may enhance uptake of the vaccine by these groups (CMOC12). Provision for privacy is an important user consideration. Pharmacy users expect private consultations to preserve their confidentiality (CMOC13).

For peer review only

For peer review only - http://bmjopen.bmj.com/site/about/guidelines.xhtml

Table 4 - Pharmacy Users (CMOCs 10-13)

**COMC 10 – Trust the pharmacist as reliable information source**[32 53 119-122]	**When pharmacy users receive information about the COVID-19 vaccine from their community pharmacy (C), they are more likely to get the vaccine (O), because they trust community pharmacy as a source of reliable, accurate information (M).**
	"When patients were educated about influenza, herpes zoster, and pneumococcal vaccines as a result of a pharmacist-driven intervention in community pharmacies, they were influenced to receive the vaccination." (USA)[119]<br><br>*"Being able to address the public enquiries with accurate up-to-date information about the local situation and the overall infection progress is the key to build trustful relationship with them at troubled times." (Macau)*[32]
**CMOC 11 – Trust the pharmacist to deliver responsive services**[7 18 23 26 34 37 43 51 54 59 63 70 84 85 123-135]	**When community pharmacies are trusted to make necessary service adaptations to ensure services are flexible, convenient and accessible (C), COVID-19 vaccine uptake is likely to be higher among pharmacy users (O) because the service is responsive to local needs (M).**
	"Pharmacists have always been the most accessible health care provider; this is especially true in the era of COVID-19….While other professionals have closed their doors to patients, community pharmacies remained open to the public despite stricter lockdown restrictions. As highly trusted healthcare clinicians, community pharmacists play a vital role in closing the gaps that are exacerbated by the additional strain on the system and reduced access to healthcare providers." (Canada)[51]

For peer review only

	"Pharmacy flu vaccination services complement those provided by general practitioners to help improve overall coverage and vaccination rates for patients in at-risk groups. These services are highly accessed by patients from all socio demographic areas, and seem to be particularly attractive to carers, frontline healthcare workers, and those of working age." (Great Britain)[135]
**CMOC 12 – Access culturally-sensitive services** *27 78 80 92 136*	**When community pharmacies leverage their community location and community-staff relationships (C), vulnerable populations, such as Black, Asian and Minority Ethnic groups are more likely to use their services, including COVID-19 vaccination services (O), because they trust their local community pharmacies to provide culturally sensitive service. (M)**
	"Research continues to highlight that patients who are medically under-served have poorer inequitable access to health care due to them experiencing greater physical barriers to accessibility, encountering poorer patient-professional communication and are significantly disadvantaged where a service is not tailored to their unique needs or preferences." (Great Britain) [137]<br><br>*"I think over the years what's happened is nationally it's almost like everything has to be the same, which then doesn't work because it doesn't accommodate all the little variations…So we need to go back and in each individual pharmacy, gear it towards the population that it is meant to be meeting the needs of" (Pharmacist caring for BAME community-Great Britain)*[137]

For peer review only

**CMOC 13 – Receive private and confidential services**[7 50 65 129 138-140]	**When community pharmacies make provisions for privacy (C), pharmacy users are more likely to use their services (O), because they are reassured about confidentiality (M).**
	"If there was something not right…the first thing I would do is make an appointment with a doctor. I wouldn't do and talk to somebody over a pharmacy counter." (Great Britain)[65]<br><br>*"Privacy, confidentiality and dignity are all vital elements of a trusting relationship between healthcare professionals and their patients…. In terms of privacy, the quality was perceived by the participants to include a confidential room that enabled private consultations." (Great Britain)*[7]

Public trust in community pharmacists is high—similar to doctors and nurses.[38] Trust between pharmacists and users can be leveraged to overcome scepticism about the COVID-19 vaccine. Providing reliable information about the disease and the vaccine, as pharmacies have done with other infections, can enhance public uptake of the COVID-19 vaccine.[113] In the USA, 90% of the US population lives within 5 miles of a community pharmacy. Given their convenience and accessibility, consumers have visited their community pharmacists 12 times more frequently than their GPs.[21] A UK study found that consumers who were eligible for a free influenza vaccine through their GPs were willing to pay for pharmacy service because of convenience and ease of access.[127] Established, trusting relationships are especially important for providing culturally sensitive services to marginalized and vulnerable communities.[129] Community pharmacists, often members of local communities are specially positioned to understand the culturally contextual factors that impact their pharmacy users.[89] Trusting relationships are founded on privacy, confidentiality and dignity,

For peer review only

and COVID-19, public health protocols and limited space must be considered—in order to maintain vital trust among pharmacy users.[65]

Discussion

This realist review sought to understand how community pharmacy can contribute to the public health agenda during the COVID-19 pandemic, particularly continuation of essential services and engagement in vaccination services.[115] As the COVID-19 vaccination service continues to evolve, our recommendations for decision makers highlight opportunities for community pharmacy to promote safe, efficient and effective service delivery.

Summary of key Findings

To optimise community pharmacy service during the pandemic, decision makers must endorse and articulate a clear role for these healthcare professionals. The public already endorses advanced roles for community pharmacy (e.g., vaccinations, minor ailment scheme), but public awareness depends on what decision makers do and say. Practical decision maker measures include adequate reimbursement to help cover the cost for time, staff and PPE (particularly for a sustainable long-term service); legal (including indemnity), regulatory coverage for advanced roles; and clear and consistent guidance for vaccination preparation and for adaptation of essential services. When given the opportunity, the permissions and resources to do so, community pharmacies have been able to adapt quickly to continue essential services and whenever possible, to offer critical advanced services. Historically, community pharmacies have significantly increased vaccination uptake (e.g., influenza vaccinations) given their accessibility and convenience and capacity to adapt to local needs for the general population and marginalized groups (e.g., BAME).

For peer review only

Compare to other similar or related studies discussing important differences in results

At the time of this realist review, there were no similar reviews on community pharmacy roles with respect to COVID-19 vaccinations. COVID-19 represents an unprecedented situation with limited direct evidence to guide decision-making. However, realist approaches engage with a wider evidence base, including research on past pandemics (e.g., SARS)[60 141 142], mass vaccination campaigns (e.g., influenza)[143], and community pharmacies' capacity pre-COVID-19 to deliver essential and advanced services.[19 144 145] Key factors previously include a lack of leadership, a lack of guidance and an increasing reliance on professional judgement and experience.[60] Research from the UK and other economically developed countries supports the 13 CMOCs and decision maker recommendations in this review (see tables 2-4 and table 5). However, limitations continue to surround the direct policy relevance of much of the community pharmacy evidence base.[146]

Study strengths and limitations

The realist approach uses diverse data, including grey literature. This feature is especially important given a novel and rapidly evolving topic area, such as COVID-19. Multiple researchers with subject matter expertise participated in screening the literature and extracting and coding data, which maximised opportunities to discuss and debate the plausibility of the inferences made. The CMOCs were developed and refined through regular discussions within a team with varied academic and clinical backgrounds. Professional and public stakeholder consultation further refined the CMOCs.

All rapid reviews operationalise coverage versus expediency. Other sources of evidence could have informed the review; however, potential gaps were mitigated by stakeholder engagement and expertise within the team. The evidence supporting the CMOCs was based on available time for document review, and during the review period, research, directives and policy related to COVID-19

For peer review only - http://bmjopen.bmj.com/site/about/guidelines.xhtml

vaccination rapidly evolved. The programme theory and its CMOCs, however, are expressed in such a way that they can be further confirmed, refuted or refined in the future using additional data.

For this rapid review, given our short timeline of six months, we initially decided to focus on community pharmacy roles and pandemic response in middle to high-income countries. Whilst we identified data from higher-income countries such as Canada, USA, Australia and France, there was very little published from middle-income countries. As such, it is likely that our findings and recommendations are most applicable to high-income countries, although, the lessons learned from high-income countries can serve as an initial reference point for best practice for other countries. The recommendations are "how to's" for other countries to consider within their own cultural and healthcare settings.

Meaning of the study: possible explanations and implications for decision makers

Although there are multiple actors involved in pandemic response, for brevity, recommendations (Table 5) are directed towards decision makers who possess the formal authority to implement the recommendations.

Table 5 - Recommendations for Decision Makers to Increase Community Pharmacy Engagement in Pandemic Response (for further details check: https://www2.aston.ac.uk/lhs/research/periscope).

**Recommendations**	**Derived from CMOC**
Articulate a clear public health agenda role for community pharmacy (e.g., COVID-19 testing and vaccination)	1

Ensure pharmacy regulations for advanced roles, such as novel vaccine administration, are in place to legally protect community pharmacists and their teams	2
Involve local community pharmacies in policy and service specification development	3
Provide timely guidance with sufficient details for community pharmacies to quickly adapt to local needs	4
Provide adequate funding and reimbursement for community pharmacy services to deliver COVID-19 vaccines	5
Equip community pharmacies with the necessary permissions to manage and adapt essential services	6
Ensure community pharmacies have the means to adequately protect the health of themselves, their staff and pharmacy users	8
Facilitate collaboration and coordination of COVID-19 vaccination services across providers (e.g., GPs, community pharmacies) and systems (e.g., IT)	9

As the CMOCs were refined, 'tensions' were uncovered with implications for decision makers, particularly: community pharmacists as healthcare professionals versus retailers[43 106 145 147]; community pharmacies' capacity to remain financially viable while managing essential and advanced services.[19 148 149] and; pharmacists' capacity to provide off-site services while maintaining physical premises.[150] These tensions stem from lack of awareness of community pharmacists as healthcare professionals.

Along the continuum of healthcare from community to hospital, community pharmacy is often the first point of contact for the public. Community pharmacies contribute to primary care services through essential services (e.g., medication dispensing) and expanded roles (e.g., vaccinations),[115 147] often decreasing workload pressures on other providers, such as GPs.[151] Public surveys demonstrate

For peer review only

For peer review only - http://bmjopen.bmj.com/site/about/guidelines.xhtml

high levels of satisfaction with community pharmacy services, and vaccination uptake is increased when pharmacies deliver these services.[149] Nevertheless, lack of public and decision maker awareness of community pharmacy primary care roles has slowed uptake and integration of these services.[121] A recent UK public survey found that community pharmacies are seen as “a medicine supply shop by 48.3% of people, as a place to purchase medicines by 22%, and a place to purchase non-medicinal products by 17.7%.” [28]

In Ireland and Canada community pharmacies are an integral part of national vaccination campaigns.[149] In both instances, community pharmacists participate in vaccination planning, pharmacy regulators provide clear guidance on vaccination management, and vaccinations are equitably refunded through public health systems. The removal of barriers, such as economic pressures on pharmacies has resulted in impressive national vaccine uptake, even in large countries, such as Canada.[149] Globally, there is an emerging trend for governments, health insurance companies and consumers to remunerate community pharmacies for services that contribute to improved health outcomes.[150]

Community pharmacies tend to be defined as premises where medications are dispensed, which compounds the confusion (by the public and decision makers) of professional primary care services versus retailers.[150] Instead, pharmacies should be defined with respect to actions or services that require specialised health knowledge to optimise health outcomes. For example, a retail approach to over-the-counter (OTC) medications is to permit consumers to make their own choices, similar to supermarket choices. Pharmacy input for into OTC purchases could potential decrease adverse medication interactions or unnecessary allergicreactions.[147] The ‘value-add’ of community pharmacies, evidence-informed engagement with consumers, can decrease morbidity and mortality outcomes and increase medication regimen adherence.[150 152]

For peer review only - http://bmjopen.bmj.com/site/about/guidelines.xhtml

Although community pharmacists can provide professional services off premises (e.g., immunizations in community and religious centres), they need to maintain their physical premises and staff for financial reasons. In addition, pharmacy users seek out community pharmacy services due to their accessibility and convenience, and without a physical space to engage regularly with pharmacy users, trust-building between pharmacists and users is compromised.[153]

As evidenced by public media, many countries, including the UK are declaring service specifications for COVID-19 vaccinations. Community pharmacists can potentially provide vaccination services in two locations.[154] They can collaborate with GPs in Primary Care Networks (PCN) to support PCN vaccination sites. Alternatively, they can provide a COVID-19 vaccination service from their premises if they meet service specifications. The described tensions create difficult choices for pharmacists that can be ameliorated through the decision maker recommendations in Table 5.

Unanswered questions and future research

A future realist evaluation involving primary data collection from key actors will inform refinement of the programme theory, CMOCs and decision maker recommendations. This empirical research will address the tensions identified above. Future research will also address issues identified through the stakeholder groups, such as vaccination hesitancy, outside the scope of this review. While this work touched upon unique issues for marginalised populations including BAME groups, those of lower socioeconomic status and those with disabilities, further exploration is needed.

We had initially focused the review on community pharmacy roles and pandemic response in middle to high-income countries, but found limited data in middle-income countries. The findings are thus more likely to be applicable to high-income countries but may serve to inform practice elsewhere. Empirical research, using for example a realist evaluation approach, should be conducted to extend

For peer review only - http://bmjopen.bmj.com/site/about/guidelines.xhtml

our findings on the role of community pharmacy in COVID vaccination programmes to middle to low income countries.

Conclusion

The COVID-19 pandemic is a worldwide health emergency. Vaccination is key to combatting the pandemic. The role of community pharmacy may be both short and long term; with a potential need for regular annual vaccines. This rapid realist review offers recommendations for decision makers to enable community pharmacy to play a key role, both during these unprecedented times and into the future.

For peer review only

For peer review only - http://bmjopen.bmj.com/site/about/guidelines.xhtml

424 ## Acknowledgement:

425 We acknowledge the contribution from professional and member of the public key stakeholder.

426 ## Research Ethics Approval

427 This research did not involve human participants and therefore ethical approval was not required.

428 ## Funders

429 Jointly funded by UKRI and NIHR COV0176. The views expressed are those of the author(s) and not
430 necessarily those of the NIHR, UKRI or the Department of Health and Social Care.

431 ## Contributorship statement

432 IM conceived and led the project and had overall responsibility for PERISCOPE. All team members
433 developed develop the embryonic programme theory. AB conducted the searches. AB and GW
434 advised on realist methods. GW also advised on primary care implications and provided
435 methodological oversight. EY screened documents at title, abstract and full-text with support from
436 AB and MM. EY, MM and JB coded relevant data into NVivo. EY, MM and JB conducted the data
437 analysis/synthesis. HZ and AH advised on pharmacy aspects and policy implications. TK led PPI. TK
438 and HZ advised on implications in BAME communities. All team members contributed drafting the
439 final report for publication and approved the final draft for submission.

440 ## Data Sharing Statement

441 Data is available upon reasonable request to the lead author.

442 ## Competing Interests

For peer review only

Geoff Wong is Deputy Chair of the NIHR HTA Prioritisation Committee: Integrated Community Health and Social Care (A) and Member of the NIHR HTA Prioritisation Committee: Integrated Community Health and Social Care (A) Methods Group.

Andrew Booth is a member of the National Institute for Health Research Health Services and Delivery Research Funding Board, the National Institute for Health Research Evidence Synthesis Programme Advisory Group and the National Institute for Health Research School for Social Care Research Commissioning Panel.

For peer review only

References

1. Parkhurst C, Singh Purewal G, Donyai P. Community Pharmacy and COVID-19—The Unsung Heroes on Our High Streets. *Journal of Patient Experience* 2020;7(3):282-84. doi: 10.1177/2374373520927638

2. Maidment I, Lawson S, Wong G, et al. Towards an understanding of the burdens of medication management affecting older people: the MEMORABLE realist synthesis. *BMC geriatrics* 2020;20(1):183. doi: 10.1186/s12877-020-01568-x [published Online First: 2020/06/06]

3. Barry HE, Hughes CM. Managing medicines in the time of COVID-19: implications for community-dwelling people with dementia. *International Journal of Clinical Pharmacy* 2020. doi: 10.1007/s11096-020-01116-y

4. Wickware C. Pharmacies' dispensing increases by up to a third as a result of COVID-19, survey finds. . *Pharm J* 2020:PJ April 2020 online. doi: 10.1211/pj.2020.20207917

5. Pharmaceutical Services Negotiating Committee. Flu Vaccination Service 2020 [Available from: https://psnc.org.uk/services-commissioning/advanced-services/flu-vaccination-service. Accessed 12/11/20]

6. Pharmaceutical Services Negotiating Committee. Vaccination Services 2020 [Available from: https://psnc.org.uk/services-commissioning/locally-commissioned-services/vaccination-services. Accessed 08/12/2020]

7. Rendell T. Community pharmacist-led influenza vaccination: a service evaluation. *The Pharmaceutical Journal* 2020. doi: 10.1211/pj.2020.20208383

8. Public Health England. Beyond the data: Understanding the impact of COVID-19 on BAME groups. London: Public Health England, 2020. [Available from: https://assets.publishing.service.gov.uk/government/uploads/system/uploads/attachment_data/file/892376/COVID_stakeholder_engagement_synthesis_beyond_the_data.pdf. Accessed 08/12/20]

For peer review only - http://bmjopen.bmj.com/site/about/guidelines.xhtml

9. Patel M. We must tear down the barriers to equality for BAME communities highlighted by COVID-19. *Pharmaceutical Journal* 2020. doi: 10.1211/PJ.2020.20208060

10. Robinson J. Ten things pharmacists should know about COVID-19 vaccines. *The Pharmaceutical Journal* 2020. doi: 10.1211/pj.2020.20208429

11. Wong G, Brennan N, Mattick K, et al. Interventions to improve antimicrobial prescribing of doctors in training: the IMPACT (IMProving Antimicrobial presCribing of doctors in Training) realist review. *BMJ Open* 2015;5(10):e009059. doi: 10.1136/bmjopen-2015-009059

12. Maidment ID, Lawson S, Wong G, et al. Health Services and Delivery Research. Medication management in older people: the MEMORABLE realist synthesis. Southampton (UK): NIHR Journals Library 2020. doi: 10.3310/hsdr08260

13. Maidment I, Young E, Wong G, et al. A rapid realist review of community pharmacy support for the public health agenda during the COVID-19 pandemic and future health emergencies. PROSPERO 2020 CRD42020194078, 2020. doi: 10.1101/2021.02.01.21250765

14. Wong G, Greenhalgh T, Westhorp G, et al. Development of methodological guidance, publication standards and training materials for realist and meta-narrative reviews: the RAMESES (Realist And Meta-narrative Evidence Syntheses – Evolving Standards) project. *Health Services and Delivery Research* 2014;2(30):1-252. doi: 10.3310/hsdr02300

15. Pharmacies across the UK close 'intermittently' to cope with increasing pressures from COVID-19. *The Pharmaceutical Journal* 2020. doi: 10.1211/pj.2020.20207844

16. Cadogan CA, Hughes CM. On the frontline against COVID-19: Community pharmacists' contribution during a public health crisis. *Research in Social and Administrative Pharmacy* 2021;17(1):2032-35. doi: 10.1016/j.sapharm.2020.03.015

17. Royal Pharmaceutical Society. RPS guidance on ethical, professional decision making in the COVID-19 Pandemic. London: Royal Pharmaceutical Society, 2020. [Available from: https://www.rpharms.com/resources/pharmacy-guides/coronavirus-covid-19/coronavirus-information-for-pharmacists/ethical-decision-making. Accessed 12/12/20]

For peer review only

18. Pharmaceutical Services Negotiating Committee. PSNC Briefing 023/20: Early guidance on the 2020/21 Seasonal Influenza Vaccination Advanced Service 2020 [Available from: https://psnc.org.uk/services-commissioning/psnc-briefings-services-and-commissioning/psnc-briefing-023-20-early-guidance-on-the-2020-21-seasonal-influenza-vaccination-advanced-service. Accessed 08/12/20]

19. Hattingh L, Sim TF, Sunderland B, et al. Successful implementation and provision of enhanced and extended pharmacy services. *Research in Social and Administrative Pharmacy* 2020;16(4):464-74. doi: 10.1016/j.sapharm.2019.06.015 [published Online First: 2019/07/06]

20. Greenhalgh T, Wong G, Westhorp G, et al. Retroduction in realist evaluation. [Internet]. The RAMESES Project 2017. [Available from: http://www.ramesesproject.org/media/RAMESES_II_Retroduction.pdf. Accessed 12/12/20]

21. Wong G. Data Gathering in Realist Reviews: Looking for needles in haystacks. Doing Realist Research: SAGE Publications Ltd, 2018:131-46. doi: 10.4135/9781526451729.n9

22. Long N. Development sociology: actor perspectives. London: Routledge 2003.

23. National Pharmacy Association. Public see pharmacies as key to the future of healthcare in wake of pandemic 2020 [Available from: https://www.npa.co.uk/news-and-events/news-item/public-see-pharmacies-as-key-to-the-future-of-healthcare-in-wake-of-pandemic. Accessed 14/01/2021].

24. Royal Pharmaceutical Society. RPS Statement on the role of pharmacists in the COVID-19 Vaccination Programme London: Royal Pharmaceutical Society; 2020 [Available from: https://www.rpharms.com/Portals/0/FINAL_Statement%20on%20COVID-19%20Vaccination_Dec%202020_.pdf?ver=2020-12-02-143304-017. Accessed 14/01/2021].

25. Strand MA, Bratberg J, Eukel H, et al. Community Pharmacists' Contributions to Disease Management During the COVID-19 Pandemic. *Preventing Chronic Disease* 2020;17. doi: 10.5888/pcd17.200317

For peer review only - http://bmjopen.bmj.com/site/about/guidelines.xhtml

26. Elbeddini A, Prabaharan T, Almasalkhi S, et al. Pharmacists and COVID-19. *Journal of Pharmaceutical Policy and Practice* 2020;13(1). doi: 10.1186/s40545-020-00241-3

27. Kirkdale CL, Nebout G, Taitel M, et al. Implementation of flu vaccination in community pharmacies: Understanding the barriers and enablers. *Annales Pharmaceutiques Françaises* 2017;75(1):9-16. doi: 10.1016/j.pharma.2016.08.006

28. Hindi AMK, Jacobs S, Schafheutle EI. Solidarity or dissonance? A systematic review of pharmacist and GP views on community pharmacy services in the UK. *Health & Social Care in the Community* 2018;27(3):565-98. doi: 10.1111/hsc.12618

29. Deslandes R, Evans A, Baker S, et al. Community pharmacists at the heart of public health: A longitudinal evaluation of the community pharmacy influenza vaccination service. *Research in Social and Administrative Pharmacy* 2020;16(4):497-502. doi: 10.1016/j.sapharm.2019.06.016

30. Sousa Pinto G, Hung M, Okoya F, et al. FIP's response to the COVID-19 pandemic: Global pharmacy rises to the challenge. *Research in Social and Administrative Pharmacy* 2021;17(1):1929-33. doi: 10.1016/j.sapharm.2020.07.004

31. Gregory PAM, Austin Z. COVID-19: How did community pharmacies get through the first wave? *Canadian Pharmacists Journal / Revue des Pharmaciens du Canada* 2020;153(5):243-51. doi: 10.1177/1715163520945741

32. Ung COL. Community pharmacist in public health emergencies: Quick to action against the coronavirus 2019-nCoV outbreak. *Research in Social and Administrative Pharmacy* 2020;16(4):583-86. doi: 10.1016/j.sapharm.2020.02.003

33. Burson RC, Buttenheim AM, Armstrong A, et al. Community pharmacies as sites of adult vaccination: A systematic review. *Human vaccines & immunotherapeutics* 2016;12(12):3146-59. doi: 10.1080/21645515.2016.1215393 [published Online First: 08/15]

For peer review only

For peer review only - http://bmjopen.bmj.com/site/about/guidelines.xhtml

34. Nadeem MF, Samanta S, Mustafa F. Is the paradigm of community pharmacy practice expected to shift due to COVID-19? *Research in Social and Administrative Pharmacy* 2021;17(1):2046-48. doi: 10.1016/j.sapharm.2020.05.021

35. Francis M, Hinchliffe A, Wales N. Vaccination services through community pharmacy. *Public Health Wales NHS Trust, Wales: NHS Wales* 2010. [Available from: http://www2.nphs.wales.nhs.uk:8080/HealthServiceQDTDocs.nsf/public/CBDDC3C0BE9449398025793B00341A6F/$file/Vaccination%20services%20through%20community%20pharmacy%20v1a.pdf. Accessed 08/12/20]

36. Clews G. Pharmacists able to administer unlicensed COVID-19 vaccines following legislation changes. *The Pharmaceutical Journal* 2020;305. doi: 10.1211/PJ.2020.20208453

37. Welsh Government, Department of Health N. I., Scottish Government, et al. Supporting pharmacists, pharmacy technicians and pharmacy teams throughout the second COVID-19 wave, 2020. [Available from: https://www.pharmacyregulation.org/news/joint-letter-supporting-pharmacy-professionals-and-teams-through-second-covid-19-wave. Accessed 12/12/20]

38. Wickware C. NHS England to choose 'selected' number of pharmacies to deliver at least 1,000 COVID-19 vaccines per week. *The Pharmaceutical Journal* 2020. doi: 10.1211/PJ.2020.20208608

39. Dyer C. Covid-19: Government poised to amend regulations to allow use of unlicensed vaccine. *The BMJ* 2020. doi: 10.1136/bmj.m3757

40. Cooper RJ. Pestle and mortal: the demise of community pharmacy in the UK. *International Journal of Pharmacy Practice* 2020;28(3):205-06. doi: 10.1111/ijpp.12635

41. Robinson J. Health professionals delivering unlicensed vaccines need government protection, say health bodies. *The Pharmaceutical Journal* 2020. doi: 10.1211/PJ.2020.20208381

For peer review only

For peer review only - http://bmjopen.bmj.com/site/about/guidelines.xhtml

42. Warner JG, Portlock J, Smith J, et al. Increasing seasonal influenza vaccination uptake using community pharmacies: experience from the Isle of Wight, England. *International Journal of Pharmacy Practice* 2013;21(6):362-67. doi: 10.1111/ijpp.12037

43. Hindi AMK, Schafheutle EI, Jacobs S. Community pharmacy integration within the primary care pathway for people with long-term conditions: a focus group study of patients', pharmacists' and GPs' experiences and expectations. *BMC Fam Pract* 2019;20(1):26-26. doi: 10.1186/s12875-019-0912-0

44. Pharmacy Voice, The Pharmaceutical Services Negotiating Committee, The Royal Pharmaceutical Society, et al. Consultation response: UK Influenza Pandemic Preparedness Strategy 2011. 2011

45. Bauman JL. Hero clinical pharmacists and the COVID-19 pandemic: Overworked and overlooked. *JACCP JAm Coll Clin Pharm* 2020. doi: 10.1002/jac5.1246

46. Stone JK, Pate AN. The Impact of COVID-19 Through the Eyes of a Fourth-Year Pharmacy Student. *American Journal of Pharmaceutical Education* 2020;84(6):ajpe8146. doi: 10.5688/ajpe8146

47. Royal College of General Practitioners. Delivering Mass Vaccinations During COVID-19 A Logistical Guide for General Practice. London: Royal College of General Practitioners, 2020. [Available from: https://www.lmc.org.uk/visageimages/Covid-19/RCGPMassVaccinationattimeofcovidV3.1.pdf. Accessed 08.12.20]

48. NHS England and NHS Improvement coronavirus. Urgent preparing for General Practice to contribute to a potential COVID-19 Vaccination Programme. In: Service NH, ed. London, 2020. [Available from: https://www.england.nhs.uk/coronavirus/covid-19-vaccination-programme/primary-care-guidance/preparing-for-gp-to-contribute-to-a-potential-covid-19-vaccination-programme. Accessed 08/12/20]

49. Wickware, C. NHS England to choose 'selected' number of pharmacies to deliver at least 1,000 COVID-19 vaccines per week. *The Pharmaceutical Journal* 2020. doi: 10.1211/pj.2020.20208608

For peer review only

50. Koster ES, Philbert D, Bouvy ML. Impact of the COVID-19 epidemic on the provision of pharmaceutical care in community pharmacies. *Research in Social and Administrative Pharmacy* 2020. doi: 10.1016/j.sapharm.2020.07.001

51. Elbeddini A, Wen CX, Tayefehchamani Y, et al. Mental health issues impacting pharmacists during COVID-19. *Journal of Pharmaceutical Policy and Practice* 2020;13(1). doi: 10.1186/s40545-020-00252-0

52. Bukhari N, Rasheed H, Nayyer B, et al. Pharmacists at the frontline beating the COVID-19 pandemic. *Journal of Pharmaceutical Policy and Practice* 2020;13 (1). doi: 10.1186/s40545-020-00210-w

53. Eidt, M. Pharmacists on the frontline. *Canadian Pharmacists Journal* 2020;153(4):186-90. doi: 10.1177/1715163520930191

54. Farrell B, Tsuyuki RT. Pharmacists step up to the challenge of COVID-19. *Canadian Pharmacists Journal* 2020;153(4):185. doi: 10.1177/1715163520930705

55. Bragazzi NL, Mansour M, Bonsignore A, et al. The Role of Hospital and Community Pharmacists in the Management of COVID-19: Towards an Expanded Definition of the Roles, Responsibilities, and Duties of the Pharmacist. *Pharmacy (Basel, Switzerland)* 2020. doi: 10.3390/pharmacy8030140

56. Chan AHY, Rutter V, Ashiru-Oredope D, et al. Together we unite: The role of the Commonwealth in achieving universal health coverage through pharmaceutical care amidst the COVID-19 pandemic. *Journal of Pharmaceutical Policy and Practice* 2020;13(1). doi: 10.1186/s40545-020-00214-6

57. Moore A. Pharmacists in one county say working with GP practices during the COVID-19 pandemic has been 'difficult'. *The Pharmaceutical Journal* 2020. doi: 10.1211/PJ.2020.20208556

For peer review only - http://bmjopen.bmj.com/site/about/guidelines.xhtml

58. Hoti K, Jakupi A, Hetemi D, et al. Provision of community pharmacy services during COVID-19 pandemic: a cross sectional study of community pharmacists' experiences with preventative ...: Springer 2020. doi: 10.1007/s11096-020-01078-1

59. NHS England and NHS Improvement coronavirus. Urgent preparing for Community Pharmacy to contribute to a potential COVID-19 Vaccination Programme. In: Service NH, ed. London, 2020. [Available from: https://www.england.nhs.uk/coronavirus/covid-19-vaccination-programme/primary-care-guidance/preparing-for-community-pharmacy-to-contribute-to-a-potential-covid-19-vaccination-programme. Accessed 08/12/20]

60. Austin Z, Martin JC, Gregory PAM. Pharmacy practice in times of civil crisis: The experience of SARS and "the blackout" in Ontario, Canada. *Research in Social and Administrative Pharmacy* 2007;3(3):320-35. doi: 10.1016/j.sapharm.2006.09.001

61. Chahine EB. The Role of Pharmacists in the Fight Against COVID-19. *Sr Care Pharm* 2020;35(7):286-91. doi: 10.4140/TCP.n.2020.286

62. Jubraj B. Remote consultations: how pharmacy teams can practise them successfully: pharmaceutical-journal.com 2020. [Available from: https://pharmaceutical-journal.com/article/ld/remote-consultations-how-pharmacy-teams-can-practise-them-successfully. Accessed 12/12/20]

63. Royal Pharmaceutical Society. All-Party Pharmacy Group warns of pressures on pharmacies London: Royal Pharmaceutical Society 2020 [Available from: https://www.rpharms.com/about-us/news/details/All-Party-Pharmacy-Group-warns-of-pressures-on-pharmacies#:~:text=The%20All%2DParty%20Pharmacy%20Group,continued%20pressures%20facing%20community%20pharmacies. Accessed 14/01/2021]

64. Barnes E, Bullock A, Allan M, et al. Community pharmacists' opinions on skill-mix and delegation in England. *International Journal of Pharmacy Practice* 2017;26(5):398-406. doi: 10.1111/ijpp.12419

For peer review only - http://bmjopen.bmj.com/site/about/guidelines.xhtml

65. Saramunee K, Krska J, Mackridge A, et al. How to enhance public health service utilization in community pharmacy?: General public and health providers' perspectives. *Research in Social and Administrative Pharmacy* 2014;10(2):272-84. doi: 10.1016/j.sapharm.2012.05.006

66. Houle SKD, Grindrod KA, Chatterley T, et al. Paying pharmacists for patient care: A systematic review of remunerated pharmacy clinical care services. *Can Pharm J (Ott)* 2014;147(4):209-32. doi: 10.1177/1715163514536678

67. Burns C. Pharmacy staff in Scotland to receive £500 bonus in recognition of COVID-19 service. *the Pharmaceutical Journal* 2020 doi: 10.1211/PJ.2020.20208614 [published Online First: 12/2020]

68. Evans AM, Wood FC, Carter B. National community pharmacy NHS influenza vaccination service in Wales: a primary care mixed methods study. *British Journal of General Practice* 2016;66(645):e248-e57. doi: 10.3399/bjgp16x684349

69. Moore A. No going back: how the pandemic is changing community pharmacy. *The Pharmaceutical Journal* 2020;305. doi: 10.1211/PJ.2020.20208309

70. Merks P, Jakubowska M, Drelich E, et al. The legal extension of the role of pharmacists in light of the COVID-19 global pandemic. *Research in Social and Administrative Pharmacy* 2020 doi: 10.1016/j.sapharm.2020.05.033

71. Zaidi STR, Hasan SS. Personal protective practices and pharmacy services delivery by community pharmacists during COVID-19 pandemic: Results from a national survey. *Research in Social and Administrative Pharmacy* 2020. doi: 10.1016/j.sapharm.2020.07.006

72. Anon. Pharmacies must be at the forefront of the COVID-19 vaccination campaign. *The Pharmaceutical Journal* 2020;305. doi: 10.1211/PJ.2020.20208546

73. Robinson J. England has lowest number of community pharmacies in five years, figures show. *The Pharmaceutical Journal* 2020. doi: 10.1211/PJ.2020.20208569

74. Wickware C. Pharmacies to be paid 50p more per flu vaccination in 2020/2021. *The Pharmaceutical Journal* 2020. doi: 10.1211/PJ.2020.20208362

For peer review only

For peer review only - http://bmjopen.bmj.com/site/about/guidelines.xhtml

75. Doyle-Price J. Pharmacies have an important role in this pandemic – but they need help to continue saving lives. *The Telegraph* 2020. [Available from: https://www.telegraph.co.uk/global-health/science-and-disease/pharmacies-have-crucial-place-communities-need-help-continue. Accessed 12/12/20]

76. Robinson J. Up to 85% of community pharmacies could be in financial deficit by 2024, economic analysis concludes. *The Pharmaceutical Journal* 2020. doi: 10.1211/PJ.2020.20208332

77. Wilkinson E, Connelly D. Is pharmacy prepared for a resurgence of COVID-19? *The Pharmaceutical Journal* 2020;305. doi: 10.1211/PJ.2020.20208340

78. Hayden JC, Parkin R. The challenges of COVID-19 for community pharmacists and opportunities for the future. *Irish Journal of Psychological Medicine* 2020;37(3):198-203. doi: 10.1017/ipm.2020.52

79. Mercadante AR, Yokota M, Hwang A, et al. Choosing Evolution over Extinction: Integrating Direct Patient Care Services and Value-Based Payment Models into the Community-Based Pharmacy Setting. *Pharmacy* 2020;8(3):128. doi: 10.3390/pharmacy8030128

80. Stewart K, Barai R, Priarities N. How to keep your community pharmacy running during the COVID-19 pandemic 2020. [Available from: https://pharmaceutical-journal.com/article/ld/how-to-keep-your-community-pharmacy-running-during-the-covid-19-pandemic. Accessed 24/02/21]

81. Hussain R, Dawoud DM, Babar Z-U-D. Drive-thru pharmacy services: A way forward to combat COVID-19 pandemic. *Research in Social and Administrative Pharmacy* 2021;17(1):1920-24. doi: 10.1016/j.sapharm.2020.07.015

82. Broughel J, Yatsyshina Y. Relax Pharmacy Regulations to Help with COVID-19 Testing and Treatment. *SSRN Electronic Journal* 2020. doi: 10.2139/ssrn.3564463

83. Pritchard RI, Huff J, Scheinberg N. Impact of Regulatory Changes on Pharmacist Delivered Telehealth During the COVID-19 Pandemic. *Journal of the American Pharmacists Association* 2020;60(6):e76-e79. doi: 10.1016/j.japh.2020.06.004

For peer review only

84. Lynch M, Leary A. COVID-19 related regulatory change for pharmacists – The case for its retention post the pandemic. *Research in Social and Administrative Pharmacy* 2020. doi: 10.1016/j.sapharm.2020.07.037

85. Erku DA, Belachew SA, Abrha S, et al. When fear and misinformation go viral: Pharmacists' role in deterring medication misinformation during the 'infodemic' surrounding COVID-19. *Research in Social and Administrative Pharmacy* 2020. doi: 10.1016/j.sapharm.2020.04.032

86. Andrawis M, Carmichael J, Collins CD, et al. Improving patient care and demonstrating value during a global pandemic: Recommendations from leaders of the Pharmacy Accountability Measures Work Group. *American journal of health-system pharmacy : AJHP : official journal of the American Society of Health-System Pharmacists* 2020. doi: 10.1093/ajhp/zxaa291

87. Kawaguchi-Suzuki M, Nagai N, Akonoghrere RO. COVID-19 Pandemic Challenges and Lessons Learned by Pharmacy Educators Around the Globe. *American Journal of Pharmaceutical Education* 2020;84(8) [published Online First: 2020 Aug 1]. doi: 10.5688/ajpe8197

88. General Pharmaceutical Council. Rising to the challenge [blog] 2020 [Available from: https://www.pharmacyregulation.org/regulate/blog/rising-challenge. Accessed 14/01/2020 2020]

89. Robinson J. Government consults on allowing pharmacy technicians to administer COVID-19 and flu vaccines. *The Pharmaceutical Journal* 2020. doi: 10.1211/PJ.2020.20208311

90. Nessel, J. Pharmacy Technicians Rise to Meet Patient Needs During Coronavirus Pandemic. *Pharmacy Times* 2020. Available from: https://www.pharmacytimes.com/view/pharmacy-technicians-rise-to-meet-patient-needs-during-coronavirus-pandemic. Accessed 08.12.20]

91. Green TC, Bratberg J, Finnell DS. Opioid use disorder and the COVID 19 pandemic: A call to sustain regulatory easements and further expand access to treatment. *Subst Abus* 2020;41(2):147-49. doi: 10.1080/08897077.2020.1752351

92. Carico R. Pharmacists continue to protect communities during COVID-19. *Journal of the American Pharmacists Association* 2020;60(5):660-61. doi: 10.1016/j.japh.2020.08.009

For peer review only

93. Bond C, Blenkinsopp A, Inch J, et al. The effect of the new community pharmacy contract on the community pharmacy workforce. *London: Pharmacy Practice Research Trust* 2008;8:9. Available from: https://pharmacyresearchuk.org/wp-content/uploads/2012/11/The_effect_of_the_new_community_pharmacy_contract_on_the_community_pharmacy_workforce.pdf. Accessed 08/12/20]

94. Melton BL, Lai Z. Review of community pharmacy services: what is being performed, and where are the opportunities for improvement? *Integr Pharm Res Pract* 2017;6:79-89. doi: 10.2147/IPRP.S107612

95. Wilson CG, Ramage M, Fagan EB. A Primary Care Response to COVID-19 for Patients with an Opioid Use Disorder. *J Rural Health* 2020. doi: 10.1111/jrh.12438

96. Kostev K, Lauterbach S. Panic buying or good adherence? Increased pharmacy purchases of drugs from wholesalers in the last week prior to Covid-19 lockdown. *J Psychiatr Res* 2020;130:19-21. doi: 10.1016/j.jpsychires.2020.07.005

97. Bell JS, Reynolds L, Freeman C, et al. Strategies to promote access to medications during the COVID-19 pandemic. *Aust J Gen Pract* 2020;49(8):530-32. doi: 10.31128/AJGP-04-20-5390

98. Romano S, Galante H, Figueira D, et al. Time-trend analysis of medicine sales and shortages during COVID-19 outbreak: Data from community pharmacies. *Research in Social and Administrative Pharmacy* 2020. doi: 10.1016/j.sapharm.2020.05.024

99. Gross AE, MacDougall C. Roles of the clinical pharmacist during the COVID-19 pandemic. *JACCP JAm Coll Clin Pharm* 2020;3(3):564-66. doi: 10.1002/jac5.1231

100. Lapeyre-Mestre M, Boucher A, Daveluy A, et al. Addictovigilance contribution during COVID-19 epidemic and lockdown in France. *Therapies* 2020. doi: 10.1016/j.therap.2020.06.006

101. Alderman C. Pharmacy Services and the Novel Coronavirus. *The Senior Care Pharmacist* 2020;35 (4):146-47. doi: 10.4140/TCP.n.2020.146

For peer review only - http://bmjopen.bmj.com/site/about/guidelines.xhtml

102. Hasan SS, Kow CS, Zaidi STR. Social distancing and the use of PPE by community pharmacy personnel: Does evidence support these measures? *Research in Social and Administrative Pharmacy* 2020;17(2):456-9. doi: 10.1016/j.sapharm.2020.04.033

103. Thomas CEL, Phipps DL, Ashcroft DM. When procedures meet practice in community pharmacies: qualitative insights from pharmacists and pharmacy support staff. *BMJ open* 2016;6(6):e010851-e51. doi: 10.1136/bmjopen-2015-010851

104. NHS England and NHS Improvement coronavirus. Standard operating procedure: COVID-19 local vaccination services deployment in community settings. London: National Health Service 2020. Available from: https://www.england.nhs.uk/coronavirus/publication/standard-operating-procedure-covid-19-local-vaccination-services-deployment-in-community-settings. Accessed 08.12.20]

105. Cabas P, Di Bella S, Giuffrè M, et al. Community pharmacists' exposure to COVID-19. *Research in Social and Administrative Pharmacy* 2021;17(1):1882-87. doi: 10.1016/j.sapharm.2020.05.020

106. Sum ZZ, Ow CJW. Community pharmacy response to infection control during COVID-19. A cross-sectional survey. *Res Soc Adm Pharm* 2020;17(1):1845-52. doi: 10.1016/j.sapharm.2020.06.014

107. Al-Quteimat OM, Amer AM. SARS-CoV-2 outbreak: How can pharmacists help? *Research in Social and Administrative Pharmacy* 2021;17(2):480-82. doi: 10.1016/j.sapharm.2020.03.018 [published Online First: 2020 Mar 26]

108. Dawoud D. Emerging from the other end: Key measures for a successful COVID-19 lockdown exit strategy and the potential contribution of pharmacists. *Research in Social and Administrative Pharmacy* 2020. doi: 10.1016/j.sapharm.2020.05.011

109. Weir NM, Newham R, Bennie M. A literature review of human factors and ergonomics within the pharmacy dispensing process. *Research in Social and Administrative Pharmacy* 2020;16(5):637-45. doi: 10.1016/j.sapharm.2019.08.029

For peer review only

780 110. Maine LL. An Unparalleled Spirit of Collaboration Amid the COVID-19 Pandemic. *Am J Pharm Educ* 2020;84(6):ajpe8155-ajpe55. doi: 10.5688/ajpe8155

782 111. Dowden A. Will Serious Shortage Protocols help ease drug shortages? *Prescriber* 2020;31(5):29-31. doi: 10.1002/psb.1844

784 112. NHS England, NHS Improvement coronavirus. Novel coronavirus (COVID-19) standard operating procedure-Community Pharmacy. [Available from: https://www.england. nhs. uk/wp-content/uploads/2020/02/20200305-COVID-19-PRIMARY-CARE-SOP-COMM-PHARM-PUBLICATION-V1. 2020 Apr;1: skills4pharmacy.org.uk 2020. Accessed 08/12/20]

788 113. Butt M. Covid-19 vaccination: Logistical challenges for general practice. *Practice Management* 2021;31(1):20-21. doi: 10.12968/prma.2021.31.1.20

790 114. National Pharmacy Association (NPA). Pharmacy on the NHS frontline 2020 [Available from: https://nhsfrontline.com/coronavirus-stories-from-the-health-service-frontline. Accessed 17/08/2020 2020]

793 115. Pharmaceutical Services Negotiating Committee. Community Pharmacy Contractual Framework: Pharmaceutical Services Negotiating Committee; 2021 [Available from: https://psnc.org.uk/contract-it/the-pharmacy-contract. Accessed 08/02/21

796 116. Quinn B. UK pharmacists facing abuse and violence during lockdown. *The Guardian* 2020 Apr 2nd 2020. [Available from: https://www.theguardian.com/world/2020/apr/02/uk-pharmacists-facing-abuse-and-violence-during-coronavirus-lockdown. Accessed 12/12/20]

799 117. Wickware C. Health board pilots delivery of flu vaccines by pharmacy technicians to manage increased demand. *The Pharmaceutical Journal* 2020. doi: 10.1211/PJ.2020.20208565

801 118. Royal Pharmaceutical Society. The future of pharmacy in a sustainable NHS: Key principles for transformation and growth. London: Royal Pharmaceutical Society, 2020. [Available from: https://www.rpharms.com/recognition/all-our-campaigns/policy-a-z/future-of-pharmacy. Accessed 08/01/21]

For peer review only - http://bmjopen.bmj.com/site/about/guidelines.xhtml

119. Sparkman A, Brookhart AL, Goode J-VKR. The impact of an immunization check-up at a pharmacist-provided employee health screening. *Journal of the American Pharmacists Association* 2017;57(3):S274-S78. doi: 10.1016/j.japh.2017.02.010

120. Rodgers RM, Gammie SM, Loo RL, et al. Comparison of pharmacist and public views and experiences of community pharmacy medicines-related services in England. *Patient Prefer Adherence* 2016;10:1749-58. doi: 10.2147/PPA.S112931

121. Hindi AMK, Schafheutle EI, Jacobs S. Patient and public perspectives of community pharmacies in the United Kingdom: A systematic review. *Health Expectations* 2017;21(2):409-28. doi: 10.1111/hex.12639

122. Letley L, Ramsay M. Discussing the MMR vaccine with patients. *The Pharmaceutical Journal* 2019. doi: 10.1211/pj.2019.20205819

123. Kember J, Hodson K, James DH. The public's perception of the role of community pharmacists in Wales. *International Journal of Pharmacy Practice* 2017;26(2):120-28. doi: 10.1111/ijpp.12375

124. Gidman W, Ward P, McGregor L. Understanding public trust in services provided by community pharmacists relative to those provided by general practitioners: a qualitative study. *BMJ open* 2012;2(3):e000939. doi: 10.1136/bmjopen-2012-000939

125. McConeghy KW, Wing C. A national examination of pharmacy-based immunization statutes and their association with influenza vaccinations and preventive health. *Vaccine* 2016. doi: 10.1016/j.vaccine.2016.04.076

126. Cheong MWL, Brock T, Karwa R, et al. COVID-19 and Clinical Pharmacy Worldwide - A Wake Up Call and a Call to Action. *JACCP JAm Coll Clin Pharm* 2020;3(5):860-63. doi: 10.1002/jac5.1286

127. Adunlin G, Murphy PZ, Manis M. COVID-19: How Can Rural Community Pharmacies Respond to the Outbreak? *J Rural Health* 2020. doi: 10.1111/jrh.12439

For peer review only

For peer review only - http://bmjopen.bmj.com/site/about/guidelines.xhtml

128. Aruru M, Truong HA, Clark S. Pharmacy Emergency Preparedness and Response (PEPR): a proposed framework for expanding pharmacy professionals' roles and contributions to emergency preparedness and response during the Covid-19 pandemic and beyond. *Research in Social and Administrative Pharmacy* 2020;17(1):1967-77. doi: 10.1016/j.sapharm.2020.04.002

129. Fiore V. GP referrals under CPCS to go live November 1, NHSE confirms. *Chemist and Druggist* 2020. [Available from: https://www.chemistanddruggist.co.uk/news/-gp-referrals-under-cpcs-live-november-1-nhse-says. Accessed 11/11/20]

130. Robinson J. Public Health England to review guidance against the use of rapid COVID-19 tests in community pharmacies. *the Pharmaceutical Journal* 2020;305. doi: 10.1211/PJ.2020.20208550

131. Buchan SA, Rosella LC, Finkelstein M, et al. Impact of pharmacist administration of influenza vaccines on uptake in Canada. *Canadian Medical Association Journal* 2016;189(4):E146-E52. doi: 10.1503/cmaj.151027

132. Calo WA, Shah PD, Gilkey MB, et al. Implementing pharmacy-located HPV vaccination: findings from pilot projects in five U.S. states. *Human Vaccines & Immunotherapeutics* 2019;15(7-8):1831-38. doi: 10.1080/21645515.2019.1602433

133. Anderson C, Thornley T. "It's easier in pharmacy": why some patients prefer to pay for flu jabs rather than use the National Health Service. *BMC Health Services Research* 2014;14(1). doi: 10.1186/1472-6963-14-35

134. Baroy J, Chung D, Frisch R, et al. The impact of pharmacist immunization programs on adult immunization rates: a systematic review and meta-analysis. *Journal of the American Pharmacists Association* 2016;56(4):418-26. doi: 10.1016/j.japh.2016.03.006

135. Anderson C, Thornley T. Who uses pharmacy for flu vaccinations? Population profiling through a UK pharmacy chain. *International Journal of Clinical Pharmacy* 2016;38(2):218-22. doi: 10.1007/s11096-016-0255-z

For peer review only - http://bmjopen.bmj.com/site/about/guidelines.xhtml

136. Pharmacy Guild of Australia. 93 per cent of Aussies recognise benefits of flu shot in pharmacy– Pharmacy Guild of Australia [blog]: Pharmacy Guild of Australia 2017. [Available from: https://www.guild.org.au/news-events/news/2017/93-per-cent-of-aussies-recognise-benefits-of-flu-shot-in-pharmacy. Accessed 12/12/20]

137. Latif A, Tariq S, Abbasi N, et al. Giving Voice to the Medically Under-Served: A Qualitative Co-Production Approach to Explore Patient Medicine Experiences and Improve Services to Marginalized Communities. *Pharmacy (Basel, Switzerland)* 2018;6(1):13. doi: 10.3390/pharmacy6010013

138. Anderson C, Blenkinsopp A, Armstrong M. Feedback from community pharmacy users on the contribution of community pharmacy to improving the public's health: a systematic review of the peer reviewed and non-peer reviewed literature 1990-2002. *Health Expect* 2004;7(3):191-202. doi: 10.1111/j.1369-7625.2004.00274.x

139. Eades CE, Ferguson JS, O'Carroll RE. Public health in community pharmacy: a systematic review of pharmacist and consumer views. *BMC public health* 2011;11:582-82. doi: 10.1186/1471-2458-11-582

140. Hassell K, Rogers A, Noyce P. Community pharmacy as a primary health and self-care resource: a framework for understanding pharmacy utilization. *Health and Social Care in the Community* 2000;8(1):40-49. doi: 10.1046/j.1365-2524.2000.00222.x

141. Alkhalili M, Ma J, Grenier S. Defining Roles for Pharmacy Personnel in Disaster Response and Emergency Preparedness. *Disaster Medicine and Public Health Preparedness* 2017;11(4):496-504. doi: 10.1017/dmp.2016.172

142. Ford H, Dallas CE, Harris C. Examining Roles Pharmacists Assume in Disasters: A Content Analytic Approach. *Disaster Medicine and Public Health Preparedness* 2013;7(6):563-72. doi: 10.1017/dmp.2013.99

For peer review only - http://bmjopen.bmj.com/site/about/guidelines.xhtml

143. Schwerzmann J, Graitcer SB, Jester B, et al. Evaluating the Impact of Pharmacies on Pandemic Influenza Vaccine Administration. *Disaster Medicine and Public Health Preparedness* 2017;11(5):587-93. doi: 10.1017/dmp.2017.1

144. Donald M, King-Shier K, Tsuyuki RT, et al. Patient, family physician and community pharmacist perspectives on expanded pharmacy scope of practice: a qualitative study. *CMAJ Open* 2017;5(1):E205-E12. doi: 10.9778/cmajo.20160135

145. Mossialos E, Courtin E, Naci H, et al. From "retailers" to health care providers: Transforming the role of community pharmacists in chronic disease management. *Health Policy* 2015;119(5):628-39. doi: 10.1016/j.healthpol.2015.02.007

146. Mossialos E, Naci H, Courtin E. Expanding the role of community pharmacists: Policymaking in the absence of policy-relevant evidence? *Health Policy* 2013;111(2):135-48. doi: 10.1016/j.healthpol.2013.04.003

147. Ilardo ML, Speciale A. The Community Pharmacist: Perceived Barriers and Patient-Centered Care Communication. *International Journal of Environmental Research and Public Health* 2020;17(2):536. doi: 10.3390/ijerph17020536

148. Daly CJ, Quinn B, Mak A, et al. Community Pharmacists' Perceptions of Patient Care Services within an Enhanced Service Network. *Pharmacy* 2020;8(3):172. doi: 10.3390/pharmacy8030172

149. Ecarnot F, Crepaldi G, Juvin P, et al. Pharmacy-based interventions to increase vaccine uptake: report of a multidisciplinary stakeholders meeting. *BMC Public Health* 2019;19(1) doi: 10.1186/s12889-019-8044-y

150. Moullin JC, Sabater-Hernández D, Fernandez-Llimos F, et al. Defining professional pharmacy services in community pharmacy. *Research in Social and Administrative Pharmacy* 2013;9(6):989-95. doi: 10.1016/j.sapharm.2013.02.005

151. NHS England. General practice forward view: NHS England, 2016. [Available from: https://www.england.nhs.uk/gp/gpfv. Accessed 12/12/20]

For peer review only - http://bmjopen.bmj.com/site/about/guidelines.xhtml

906 152. Milosavljevic A, Aspden T, Harrison J. Community pharmacist-led interventions and their impact on patients' medication adherence and other health outcomes: a systematic review. *The International journal of pharmacy practice* 2018;26(5):387-97. doi: 10.1111/ijpp.12462 [published Online First: 2018/06/22]

910 153. Justwan F, Baumgaertner B, Carlisle JE, et al. The effect of trust and proximity on vaccine propensity. *PLOS One* 2019;14(8):e0220658. doi: 10.1371/journal.pone.0220658

912 154. Pharmaceutical Services Negotiating Committee. Preparations for a potential COVID-19 vaccination programme.: Pharmaceutical Services Negotiating Committee; 2020 [Available from: https://psnc.org.uk/our-news/preparations-for-a-potential-covid-19-vaccination-programme. Accessed 08/11/2020]

916

For peer review only

Appendix 1 - OVID MEDLINE – Search Strategy

1. exp Coronavirus/ or exp Coronavirus Infections/ or (coronavirus* or "2019-nCoV" or "2019 ncov" or nCov or "Covid 19" or Covid19 or "SARS CoV 2" or novel coronavirus or novel corona virus or covid* or "coronavirus 2" or coronavirus infection* or coronavirus disease or corona virus disease or new coronavirus or new corona virus or new coronaviruses or novel coronaviruses or wuhan).mp. [mp=title, abstract, original title, name of substance word, subject heading word, floating sub-heading word, keyword heading word, organism supplementary concept word, protocol supplementary concept word, rare disease supplementary concept word, unique identifier, synonyms]

2. (Community pharmacy or community pharmacies or community pharmacist or community pharmacists or retail pharmacy or retail pharmacist or retail pharmacists or dispensing chemist or dispensing chemists or (((Pharmacist or pharmacy) and (community or primary care)) or dispensary or apothecary or druggist)).mp. [mp=title, abstract, original title, name of substance word, subject heading word, floating sub-heading word, keyword heading word, organism supplementary concept word, protocol supplementary concept word, rare disease supplementary concept word, unique identifier, synonyms]

3. 1 and 2

NB. No Date or Language Limits were applied.

For peer review only

Appendix 2 - Literature inclusion criteria

Published after January 2003 AND
COVID-19 or other pandemic or other infectious diseases
OR vaccination programmes
OR expanded/extended roles
AND Community Pharmacy
AND High- or middle-income country

For peer review only

PRISMA 2009 Flow Diagram

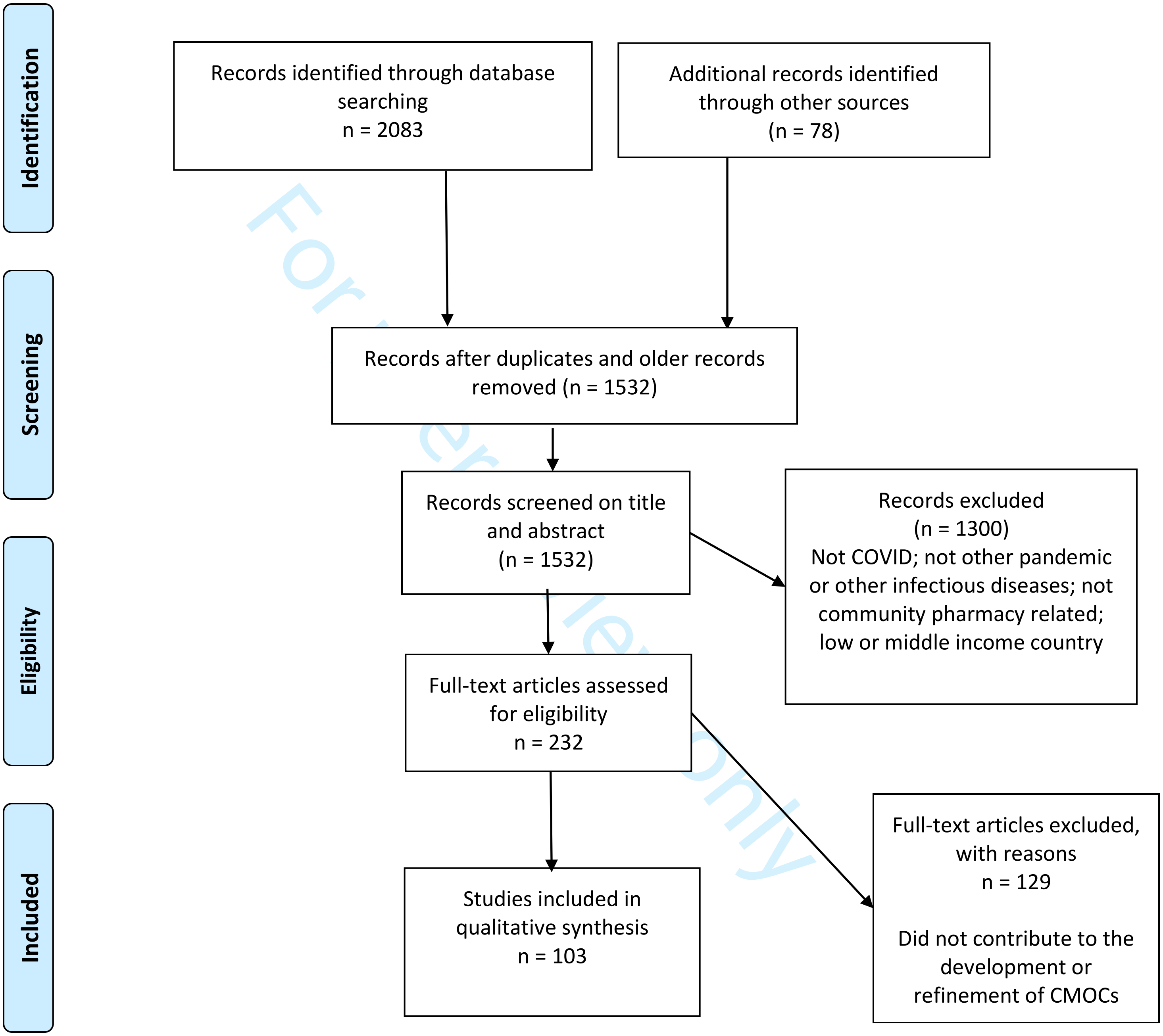

From: Moher D, Liberati A, Tetzlaff J, Altman DG, The PRISMA Group (2009). *P*referred *R*eporting *I*tems for *S*ystematic Reviews and *M*eta-*A*nalyses: The PRISMA Statement. PLoS Med 6(7): e1000097. doi:10.1371/journal.pmed1000097

For more information, visit www.prisma-statement.org.

For peer review only - http://bmjopen.bmj.com/site/about/guidelines.xhtml

Appendix 4 - A simplified diagram of the programme theory of a COVID-19 vaccination programme provided by community pharmacies

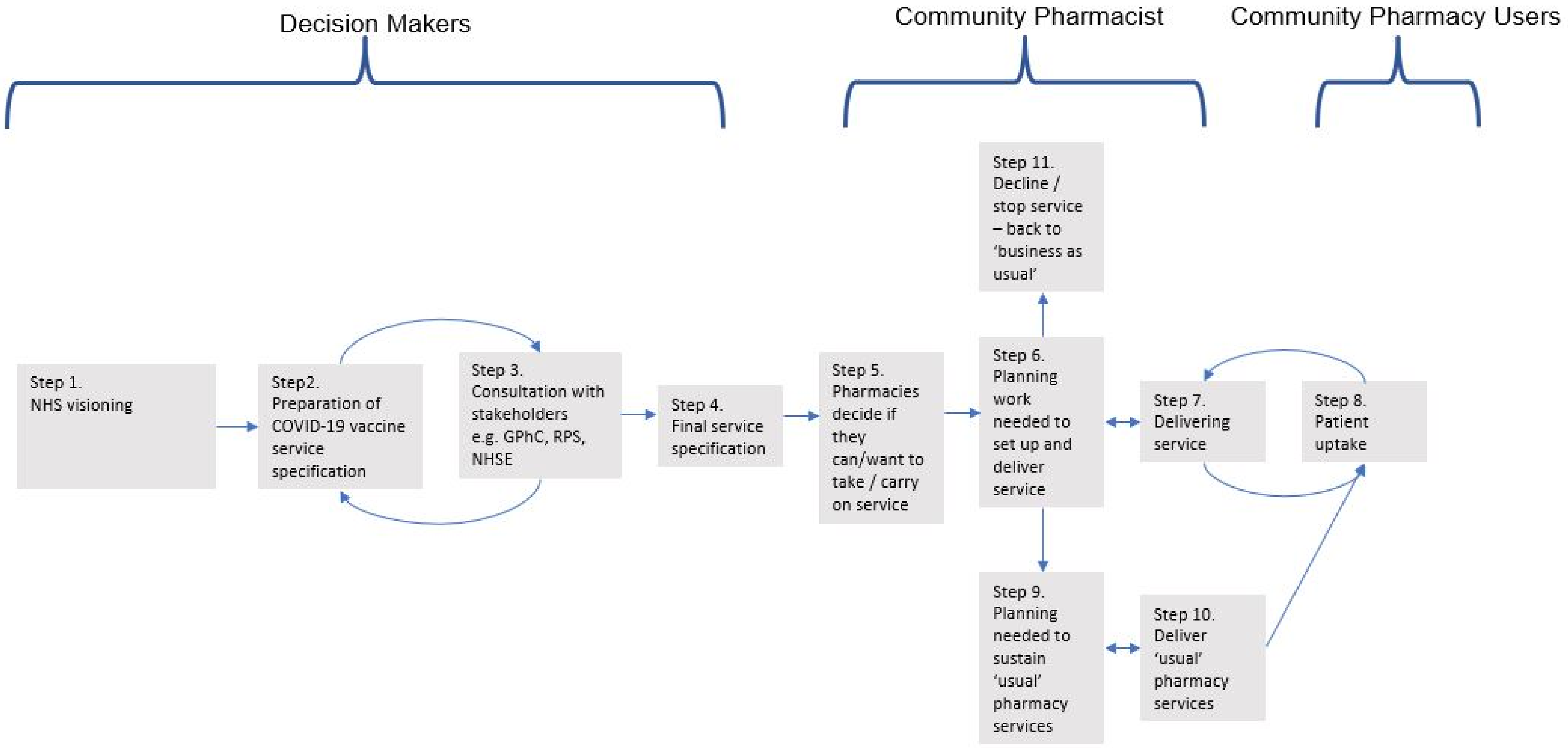

BMJ Open

A Rapid Realist Review of the Role of Community Pharmacy in the Public Health Response to COVID-19

Journal:	*BMJ Open*
Manuscript ID	bmjopen-2021-050043.R2
Article Type:	Original research
Date Submitted by the Author:	13-Apr-2021
Complete List of Authors:	Maidment, Ian; Aston University<br>Young, Emma; The University of Sheffield<br>MacPhee, Maura; The University of British Columbia<br>Booth, Andrew; The University of Sheffield, School of Health & Related Research (ScHARR)<br>Zaman, Hadar; University of Bradford<br>Breen, Juanita; University of Tasmania<br>Hilton, Andrea; University of Hull, FHSC<br>Kelly, Tony; Aston University, PPI Lead on Project<br>Wong, Geoff; University of Oxford, Primary Care Health Sciences
**Primary Subject Heading**:	Health services research
Secondary Subject Heading:	General practice / Family practice, Infectious diseases
Keywords:	COVID-19, QUALITATIVE RESEARCH, Organisation of health services < HEALTH SERVICES ADMINISTRATION & MANAGEMENT

SCHOLARONE™
Manuscripts

I, the Submitting Author has the right to grant and does grant on behalf of all authors of the Work (as defined in the below author licence), an exclusive licence and/or a non-exclusive licence for contributions from authors who are: i) UK Crown employees; ii) where BMJ has agreed a CC-BY licence shall apply, and/or iii) in accordance with the terms applicable for US Federal Government officers or employees acting as part of their official duties; on a worldwide, perpetual, irrevocable, royalty-free basis to BMJ Publishing Group Ltd ("BMJ") its licensees and where the relevant Journal is co-owned by BMJ to the co-owners of the Journal, to publish the Work in this journal and any other BMJ products and to exploit all rights, as set out in our licence.

The Submitting Author accepts and understands that any supply made under these terms is made by BMJ to the Submitting Author unless you are acting as an employee on behalf of your employer or a postgraduate student of an affiliated institution which is paying any applicable article publishing charge ("APC") for Open Access articles. Where the Submitting Author wishes to make the Work available on an Open Access basis (and intends to pay the relevant APC), the terms of reuse of such Open Access shall be governed by a Creative Commons licence – details of these licences and which Creative Commons licence will apply to this Work are set out in our licence referred to above.

Other than as permitted in any relevant BMJ Author's Self Archiving Policies, I confirm this Work has not been accepted for publication elsewhere, is not being considered for publication elsewhere and does not duplicate material already published. I confirm all authors consent to publication of this Work and authorise the granting of this licence.

For peer review only

A Rapid Realist Review of the Role of Community Pharmacy in the Public Health Response to COVID-19

Ian Maidment (corresponding author), Aston University, Birmingham, B4 7ET, UK, i.maidment@aston.ac.uk, Phone/fax: 0121-204-3000; Emma Young, University of Sheffield, Sheffield, UK; Maura MacPhee, University of British Columbia, Vancouver, Canada; Andrew Booth, University of Sheffield, Sheffield, UK; Hadar Zaman, University of Bradford, Bradford, UK; Juanita Breen, University of Tasmania, Hobart; Andrea Hilton, University of Hull, Hull, UK; Tony Kelly, PPI Lead (Aston University), Birmingham, UK; Geoff Wong, University of Oxford, Oxford, UK.

Keywords: COVID-19, SARS-CoV-2, Community Pharmacy Services, Vaccination

Word count: 3,744

For peer review only - http://bmjopen.bmj.com/site/about/guidelines.xhtml

Abstract

Introduction

Community pharmacists and their teams have remained accessible to the public providing essential services despite immense pressures during the COVID-19 pandemic. They have successfully expanded the influenza vaccination programme and are now supporting the delivery of the COVID-19 vaccination roll-out.

Aim

This rapid realist review aims to understand how community pharmacy can most effectively deliver essential and advanced services, with a focus on vaccination, during the pandemic and in the future.

Method

An embryonic programme theory was generated using four diverse and complementary documents along with the expertise of the project team. Academic databases, preprint services and grey literature were searched and screened for documents meeting our inclusion criteria. The data was extracted from 103 documents to develop and refine a programme theory using a realist logic of analysis. Our analysis generated 13 context-mechanism-outcome configurations explaining when, why and how community pharmacy can support public health vaccination campaigns, maintain essential services during pandemics, and capitalise on opportunities for expanded, sustainable public health service roles. The views of stakeholders including pharmacy users, pharmacists, pharmacy teams and other healthcare professionals were sought throughout to refine the 13 explanatory configurations.

Results

The 13 context-mechanism-outcome configurations are organised according to decision makers, community pharmacy teams and community pharmacy users as key actors. Review findings include:

For peer review only

supporting a clear role for community pharmacies in public health; clarifying pharmacists' legal and professional liabilities; involving pharmacy teams in service specification design; providing suitable guidance, adequate compensation and resources; and leveraging accessible, convenient locations of community pharmacy.

Discussion

Community pharmacy has been able to offer key services during the pandemic. Decision makers must endorse, articulate and support a clear public health role for community pharmacy. We provide key recommendations for decision makers to optimise such a role during these unprecedented times and in the future.

Strengths and limitations of this study

A diverse group of professional and public stakeholders validated our findings from the literature.

By using a realist approach, we were to use broad range of data, including grey literature.

To enable us to develop recommendations in a timely manner the focus of the review was deliberately narrow.

The topic could have been informed by other sources of evidence in particular empirical interviews with key stakeholder.

COVID-19 vaccination is a rapidly evolving area and this research was based on the best available evidence at the time.

For peer review only - http://bmjopen.bmj.com/site/about/guidelines.xhtml

65

For peer review only

For peer review only - http://bmjopen.bmj.com/site/about/guidelines.xhtml

Introduction

Community pharmacy teams have continued to provide essential services during the coronavirus disease 2019 (COVID-19) pandemic. They offer accessibility and medicines expertise to the public, even in challenging times.[1 2 3] However, COVID-19 creates extra workload demands, such as medication dispensing with increases of up to 33% in prescription numbers.[4] To cope with this demand community pharmacies have increased their opening hours and hired additional staff.[4] Alongside this additional workload, they have managed widening coverage of the influenza vaccines programme.[5]

Evidence suggests that community pharmacy can successfully provide diverse vaccination services including seasonal and pandemic influenza, travel vaccinations and hepatitis B for at-risk groups, within the provisions of the UK National Health Service (NHS) or privately.[6] They have successfully provided influenza vaccines as an NHS commissioned advanced service since 2015.[5] One service evaluation found that of 485 patients asked, 99% expressed confidence in their pharmacist to provide additional vaccinations.[7] Community pharmacy can also support influenza and other vaccinations to combat the significantly higher COVID-19 related mortality in ethnic minorities (excluding White minorities).[8 9]

The COVID-19 pandemic has stretched NHS capacity to safely and efficiently meet public health demands. A role for community pharmacy in the national vaccination service requires an understanding of what pharmacy teams require to successfully deliver essential and advanced services during the pandemic. Such knowledge is timely, given the roll-out of COVID-19 vaccines across the community.[10]

Delivering a vaccine is a complex process and successful delivery is context-dependent. A realist review helps make sense of complex situations[11 12], such as how community pharmacy can most

For peer review only

effectively address the challenges presented by COVID-19. A rapid review can generate guidance for decision makers to assist with roll-out of COVID-19 vaccinations to community pharmacy. This rapid realist review aimed to understand how community pharmacy can most effectively deliver essential and advanced services, with a focus on vaccination, during the pandemic and in the future.

Methods

A rapid realist review of academic and other literature, supplemented by input from key actors, was undertaken to understand how and when community pharmacy can effectively support the public health agenda during pandemics such as COVID-19. Rapid reviews aim to ensure findings are generated and disseminated in response to the urgent nature of the situation. To produce this knowledge at pace, we truncated the following review processes:

- Programme theory development was undertaken within 1 month with input from the project team.
- Searching was expedited using broad search terms and using a limited number of key data sources.
- Data analysis and context-mechanism-outcome configuration (CMOC) development focussed on where the programme theory was considered most important during COVID-19.

This realist review was undertaken within six-months (August 2020 - January 2021), the protocol was published on PROSPERO[13] and, where relevant, follows the RAMESES quality and publication standards.[14]

Stage 1: Programme Theory Development

The project team met virtually to develop an embryonic programme theory using four diverse documents from an initial search representing a professional journal[15], a research journal[16], a policy document (Royal Pharmaceutical Society)[17] and a practical influenza briefing.[18] The team identified the need for (i) enabling guidance for community pharmacy (to achieve legitimisation)[16]; (ii) practical

For peer review only - http://bmjopen.bmj.com/site/about/guidelines.xhtml

114 direction for community pharmacy practices (to ensure feasibility)[15 16]; and (iii) user assurance of appropriate, safe, feasible and timely intervention (relative advantage).[15] The resultant embryonic theory, patterned on a COM-B behavioural model of capability, opportunity, and motivation leading to behaviour[19], was used to inform searching and initial analyses.

Stage 2: Literature Searching

Searches were conducted (July-August 2020) using MEDLINE, EMBASE, CINAHL, Web of Science, and Scopus for search concepts relating to Pharmacy and COVID by AB (see Appendix 1 for search strategy). Reference checking and citation searching of all included references on Google Scholar (using the Publish or Perish tool) were also carried out. Given the novelty of the virus, we searched the contents of preprint services and the World Health Organisation (WHO) COVID Register. Grey literature searches included social media (e.g. blogs, facilitated Twitter® discussion [#Cpharmchat]), community pharmacy websites, and emails from relevant regulators and professional organisations (e.g., Royal Pharmaceutical Society [RPS], Pharmaceutical Services Negotiation Committee [PSNC], General Pharmaceutical Council [GPhC]).

Key inclusion criteria were high or middle-income countries, community pharmacy and infectious disease management (see Appendix 2). The search covered January 2003-July 2020 to include SARS, a comparable condition first identified in 2003. There were no restrictions on study designs eligible for inclusion.

Stage 3: Data Selection and Extraction (selection and coding)

Selection and appraisal of documents followed a two-step procedure:

1). EY screened the title, abstract and keywords of potentially relevant documents against inclusion criteria. A 10% random sample was checked by two research team members (AB and MM) for consistency.

For peer review only

2). EY obtained and screened full texts of all documents meeting the eligibility criteria.

Relevant data from the included full text documents was coded into NVivo by EY, MM and JB. Some codes came from the data (i.e. inductive coding); others were derived from the programme theory (i.e. deductive coding) and some were derived using retroduction (i.e. by interpretation of what might be functioning as mechanisms).[20] No assessment was made of the rigour of the data within included documents, however global judgements were made of the quality of the explanations provided by the CMOCs and programme theory using the criteria of consilience, simplicity and analogy.[21]

Stage 4: Data Synthesis

The data analysis/synthesis was conducted by EY, MM and JB with input from the rest of the project team to develop and refine the programme theory using a realist logic of analysis. Our analysis generated 13 realist CMOCs, explaining when, why and how community pharmacy can support public health vaccination campaigns, maintain essential services during pandemics, and capitalise on opportunities for expanded, sustainable public health service roles. Actor conversations generated further CMOCs related to care for diverse and vulnerable populations, including ethnic minorities.

Our realist logic of analysis centred on the following questions:

- Interpretation of meaning: Do the contents coded by the team provide data that may be interpreted as CMOCs?
- Interpretations and judgements about CMOCs: How do the CMOCs relate to the programme theory?
- Interpretations and judgements about programme theory: How do the programme theory and its CMOCs correspond with key actor perspectives of reality?

For peer review only

For peer review only - http://bmjopen.bmj.com/site/about/guidelines.xhtml

Data to answer our questions was iteratively sought across documents. Interpretive cross-case comparison was used to identify and to explain the "success" of pandemic community pharmacy interventions delivered in different settings or to different population groups.

Key Stakeholders

Key stakeholders, including community pharmacists and support staff (including representatives from large and smaller chains, sole independent pharmacies and primary care), other healthcare professionals and members of the public were consulted on four occasions. The meetings took place over Microsoft Teams and each lasted about one hour.

Patient and Public Involvement

Members of the public were drawn from The University of Sheffield's Patient and Public Involvement database and contacts of the core project team. Groups numbered between 11 and 13 members, with ages ranging from 22 to 74 years, from diverse locations and ethnicities including Black African, Black Caribbean, British Asian, British Chinese, White Irish and White British. Collectively, stakeholders provided feedback and advice on their real-world experience of working in or using community pharmacy.

Results

103 documents were included in this rapid review and were coded to develop and refine our CMOCs and programme theory (PRISMA diagram in Appendix 3). The final programme theory is summarised in Appendix 4—from abstract visioning to actual patient uptake of the COVID-19 vaccine. Although the programme theory is outlined in a linear fashion, steps within it are not necessarily linear and may occur simultaneously. The CMOCs are organized according to key actors, or individuals and groups with a vested interest in community pharmacy delivery of a COVID-19 vaccination

For peer review only - http://bmjopen.bmj.com/site/about/guidelines.xhtml

programme. "Actor" derives from sociology and is synonymous with "stakeholder"; we privileged this term to differentiate programme theory/CMOC organization from project stakeholder participants.[22] Table 1 briefly describes the three actor groups with their respective CMOCs and corresponding steps in Appendix 4. Tables 2-4 show the final 13 CMOCs.

Table 1: Programme Theory Actors with corresponding CMOCs and steps

Programme Theory Actors	Description	CMOCs*	Steps in Programme Theory**
Decision Makers	The UK government, regulatory and professional bodies, the public.	CMOC 1 - 4	Steps 1 - 4
Community Pharmacists and their Teams	Community pharmacists are health care professionals registered by the General Pharmaceutical Council and supported by teams made up of counter assistants, dispensers and registered technicians. They work in high street locations, in local communities and in supermarkets. Employers range from large chains to small individually owned community pharmacies.	CMOC 5 – 9	Steps 5 – 7 and 9 – 11
Pharmacy Users	Members of the public who use any community pharmacy services including prescription dispensing, minor ailment advice/treatment or vaccination services.	CMOC 10 - 13	Step 8

*see Table 2-4

**See Appendix 4

For peer review only - http://bmjopen.bmj.com/site/about/guidelines.xhtml

For peer review only

The following sections summarise the CMOCs related to each of the three actor groups. Illustrative examples of the supporting evidence from review documents are presented (Tables 2-4).

Decision makers

Decision maker and public endorsement (CMOC 1) are essential first steps in enlisting community pharmacy for COVID-19 vaccination programmes. Regulators must ensure pharmacists have the legal scope to do so (CMOC 2), with community pharmacy input during the development of policies and protocols (CMOC 3), so that final service specifications are flexible and do-able within local settings (CMOC 4).

Table 2 - Decision Makers (CMOCs 1-4)

**CMOC 1 – Support a public health role**[23 24 25 26-32]	**When the government, pharmacy regulators, professional bodies and the public endorse and support a clear role for community pharmacy in public health services (C), community pharmacists will be more likely to adopt vaccination services (O) because they see it as their professional role and duty (M).**
	"Distribution and administration of the COVID-19 vaccination programme will require concerted action across the NHS. With unique insight and expertise in medicines and the delivery of vaccination programmes, pharmacists have a clear role in contributing to the success of this programme." (Great Britain)[24]<br><br>*"55% of the public have visited a pharmacy during the COVID19 crisis…89% of people believe pharmacies are playing an essential role in the COVID19 crisis." (UK National Pharmacy Association)*[23]

For peer review only - http://bmjopen.bmj.com/site/about/guidelines.xhtml

	"Pharmacists…have been called on to coordinate the administration of COVID-19 tests...providing ongoing COVID-19 surveillance to communities by allowing walk-in testing at community pharmacies…[This] might be more sustainable and convenient than the large-scale public screening being done as of the summer of 2020." (USA)[25]<br><br>*"Given the past success of community pharmacists with increasing annual seasonal influenza uptake and their accessibility, pharmacists will need to be central in administering COVID-19 vaccines in order to achieve rapid population-wide coverage." (Canada)*[26]
**CMOC 2 –Clarify legal and professional liabilities**[24 31 33-40]	**When pharmacy regulators and the NHS clarify community pharmacists' legal and professional liabilities arising from the administration of a novel and potentially unlicensed COVID-19 vaccine (C), community pharmacists are more willing to give the vaccination (O) because they feel reassured regarding liability (M).**
	"The role of pharmacists in the COVID-19 vaccination programme must be made clear to the pharmacy profession itself. Professional and representative pharmacy bodies have an important role to play in providing the right level of information to the profession to support their roles in the vaccination programme." (Great Britain)[24]<br><br>*"Indemnity insurance for individual healthcare professionals needs to be amended to cover this activity and be state-funded. There also needs to be clear communication to healthcare professionals, so they clearly understand*

For peer review only - http://bmjopen.bmj.com/site/about/guidelines.xhtml

	that they are covered and under which circumstances this applies." (Great Britain)[41]
**CMOC 3 – Co-develop feasible service specifications**[1 38 42-48]	**When COVID-19 vaccination policy and service specifications have been developed with input from diverse community pharmacists and staff tasked with administering and supporting the administration of the vaccine (C), community pharmacies are more likely to deliver the service (O) because they believe the service specification is feasible (M).**
	"Pharmacists ideally want input into future policy changes before they are finalized, so that these can reflect capacity and preparedness on the ground and be publicized accurately." (Great Britain)[1]
**CMOC 4 – Issue clear, relevant and timely guidance**[25 27 31 34 48-62]	**When government, pharmacy regulators and professional bodies provide consistent, clear, relevant, and timely guidance for the delivery of the COVID-19 vaccines (C), community pharmacies are more likely to deliver the service (O), because the guidance is helpful and simplifies implementation (M).**
	"… it is good to see that NHSE/I have provided the information we have been waiting for to review the resources we have nationwide and decide how we can bring them to bear to help the NHS defeat this virus." (Great Britain)[49]

203

204 In England community pharmacies have government contracts and partnerships to deliver
205 vaccinations and other essential services during emergencies, including the COVID-19 pandemic.[25 31
206 32] Pharmacies in the USA and Canada have also been identified as having a substantive role in
207 vaccine administration (CMOC1).[25,26] The idea of harnessing UK community pharmacy capacity
208 enjoys widespread public support (CMOC2).[23] However, appropriate service delivery is hampered by
209 unfeasible operational conditions (CMOC3)[1]; for example, medication deliveries are funded by the
210 UK government for "vulnerable people", but this category of service users is defined narrowly by the

For peer review only - http://bmjopen.bmj.com/site/about/guidelines.xhtml

government and misunderstood by the public, creating unrealistic expectations of community pharmacy and generating additional work.[1]

In contrast, when clear options for community pharmacy involvement in COVID-19 vaccination programmes were issued through the NHS (CMOC4), the chief executive of the Association of Independent Multiple Pharmacies commented positively on members' engagement in delivering vaccines.[49]

Community Pharmacists and Team

Community pharmacies have had to manage ongoing essential services, in addition to supporting COVID vaccination delivery during the pandemic (CMOC6), including delivery of necessary medications (CMOC7). Given fears and anxieties related to COVID-19 and changes to service delivery, community pharmacies have had to deal with inappropriate behaviours from the public, including emotional abuse and threats of physical abuse (CMOC8). As an integral public health service, community pharmacy capacity to meet NHS needs will be enhanced through use of IT and collaboration with other service providers (CMOC9).

Table 3 - Community Pharmacists and Team (CMOCs 5-9)

**CMOC 5 – Receive adequate compensation and resources**[1 63-77]	**When community pharmacies receive adequate compensation and resource support for COVID-19 vaccines (C), they are more likely to deliver the service (O), because they feel recognised for their service contributions (M).**
	"Pharmacists…have been incredible in supporting patients throughout COVID-19 and rightly deserve recognition for the work they do. We know that teams were already under pressure and that colleagues in community pharmacy are feeling added financial strain." (Great Britain).[63]

For peer review only - http://bmjopen.bmj.com/site/about/guidelines.xhtml

	"In the community, mitigating the impact of COVID-19 has…focused largely on general practitioners (GPs). This is unsurprising to those in the pharmacy profession who have long considered policy makers to overlook them." (Great Britain)[1]
**CMOC 6 – Sustain capacity and facility to adapt essential services**[16 17 30 34 68 70 71 78-91 92]	**When a community pharmacy has the capacity and permission to manage and adapt existing essential services during COVID-19 (C), they are more likely to effectively deliver and sustain these services (O) because it's feasible for them to do so (M).**
	"Virtual and telephone consultations have become commonplace, particularly to vulnerable patients. Pharmacists have implemented systems to dispense medications in advance of need to minimise wait times and duplicate visits. In case-by-case examples…there has been anticipatory management of medication-related needs." (Ireland)[92]<br><br>*"Pharmacists who had comfort and confidence in managing electronic communication reported feeling greater control over workflow and the ability to triage and queue patients more effectively based on priority and need." (Canada)*[60]
**CMOC 7 – Inform users of essential service availability/continuity**	**When community pharmacies have processes in place to inform pharmacy users about the availability of essential services (C), pharmacy users are less likely to become anxious (O), because they feel reassured about access to what they need (M).**

For peer review only - http://bmjopen.bmj.com/site/about/guidelines.xhtml

92 16 28 31 32 51 60 65 71 80 81 85 93-101	*"Access to medications is a main concern expressed by our patients… The CDC and SAMHSA offer guidance on ways to reduce stress and anxiety in this time of uncertainty."*[95]<br><br>*"As a result of the power dynamics at play, it is ultimately up to pharmacists to be able to reassure patients and provide care, all while taking into account their mental health. Currently, guidelines regarding patient interaction during a pandemic are needed".*[51]<br><br>*"In addition, there is ongoing work in liaison with pharmacists, general practitioners, and state and territory authorities to enable therapeutic substitution by pharmacists in the event of a shortage. This will allow community pharmacists to substitute dose strength or form without prior approval from the prescriber, if a prescribed medicine is not available at the time of dispensing. These measures highlight the important role pharmacists can play in enabling and maintaining access to medicines for people in need throughout the COVID-19 outbreak." (Australia)*[85]<br><br>*"Access to medications is a main concern expressed by our patients… The CDC and SAMHSA offer guidance on ways to reduce stress and anxiety in this time of uncertainty." (USA)*[95]
**CMOC 8 – Protect the health and safety of staff** [24 31 51 71 77 81 102-108]	**When community pharmacies have the means to protect the health of pharmacists and staff (C), they are more likely to deliver essential services and COVID-19 vaccination services (O), because they feel safe to do so (M).**

For peer review only - http://bmjopen.bmj.com/site/about/guidelines.xhtml

	"…Given high levels of public anxiety and uncertainty regarding the integrity of the drug supply chain in Canada, many participants reported difficult, sometimes frightening, interactions with members of the public and their desire for dedicated security to provide support and conflict management" (Canada).[31]<br><br>*"An additional burden Asian pharmacists face, on top of pharmacist harassment, is the rise of anti-Asian racism that has come about due to COVID-19. Verbal and even physical abuse has been reported to happen in various countries, such as the UK, France, and the USA, to those of Chinese descent…" (UK, France, USA)*[51]<br><br>*"The survey found that although more than a third (37%) of pharmacists said they felt unsafe at some point working during the pandemic, concerns over PPE have eased. This is due, in part, to the supply problems being eased and community pharmacists being finally allowed to order from the national online PPE portal in an emergency, after calls for access from the RPS." (Great Britain)*[77]
**CMOC 9 – Enhance collaboration across services, including IT**[18 26 47 56 57 69 78 80 85 109-114]	**When systems needed for COVID-19 vaccination, including IT and remuneration, support collaboration by various service providers (e.g. general practitioner surgeries and community pharmacies: (C), a coordinated response is more likely (O), because reduced effort is needed from everyone (M).**

For peer review only - http://bmjopen.bmj.com/site/about/guidelines.xhtml

	"Planning and delivery should be undertaken across a consistent, pre-agreed footprint. It may be more efficient and cost effective to provide immunisation across a number of providers, pooling resources and sites to deliver the best service possible, and working in coordination with other local stakeholders such as directors of public health and local government." (Great Britain)[47]<br><br>*"For many community pharmacists, a lack of connected IT is a huge problem. Kieran Eason, who runs an independent pharmacy in Tamworth, Staffordshire, says lack of intra-operability makes it more difficult to do relatively simple things, like sending prescription requests to GPs. "Pharmacy IT is just a complete disaster," he says, suggesting the COVID-19 crisis has highlighted flaws, such as the number of different systems pharmacists use." (Great Britain)*[69]

Although attention is focused on a COVID-19 vaccine, community pharmacies offer diverse essential services. In the UK and other high-income countries, community pharmacy services also provide advanced services such as vaccinations.[115] Pharmacists consider it a professional responsibility to provide essential services during the pandemic, despite clear financial risks to themselves.[63]

Despite pharmacists' professional and moral obligations to provide essential services, ongoing persistent 'under-recognition' can jeopardize their ability to contribute to COVID-19 vaccinations (CMOC5) as well as maintain a usual service. Under-recognition has been an issue in previous UK vaccination campaigns; respondents to a survey of Welsh community pharmacists after the 2016 influenza season described providing a "mop up" service for general practitioners (GPs).[59 68]

For peer review only

For peer review only - http://bmjopen.bmj.com/site/about/guidelines.xhtml

Multiple required community pharmacy service adaptations have been reported by the UK Pharmaceutical Journal, including changeover in retail space to medication preparation and dispensing, and call-in shopping services for other retail items (CMOC6). Service adaptations have been affected by hours of operation, available staff, and cancellations of contracted services, such as blood pressure testing and smoking cessation support—all with financial implications for pharmacists.[80]

Before COVID-19, community pharmacies globally were offering "valued-added services" (VAS) such as drive-thru services, online ordering and communications services (e.g., prescription reminders) to stay competitive. Many VAS services have helped pharmacies adapt more quickly to pandemic restrictions.[81]

During the pandemic, ensuring access to needed medications has been a critical pharmacy service, to allay public concerns (CMOC7).[16] Potential and actual disruptions in expected services and needed supplies (e.g., medications) have resulted in tensions, threats and verbal/physical abuse by the public to community pharmacists (CMOC 8).[51 80 116] Safety policies, protocols and safety-related supplies (e.g., PPEs) must in place to ensure community pharmacy teams' safety.[80]

The UK COVID-19 vaccination campaign borrows heavily from previous, successful collaborative influenza vaccination programmes using community pharmacy and GPs (CMOC9).[48] Pooling resources improves service delivery.[117] For example, the UK Pharmaceutical Journal reported how a greater collaborative approach has resulted in successful influenza vaccination of home care staff and domiciliary workers during the pandemic.[117]

The pandemic has clearly demonstrated the importance of interoperable, connected IT systems across services.[118] Getting access to reliable information is important for tracking supplies and deliveries related to the COVID-19 vaccination programme.[69] A pandemic silver lining is raised awareness of IT functions for enhanced delivery of essential services (e.g., medication planning,

For peer review only - http://bmjopen.bmj.com/site/about/guidelines.xhtml

prescribing and dispensing between pharmacies and GPs) and advanced services, including vaccinations.[69]

Pharmacy Users

Pharmacy users trust community pharmacies as a reliable source of information (CMOC10) about vaccines, and pharmacies' local accessibility and convenience increases the likelihood of users obtaining COVID-19 vaccines through them (CMOC11). Community pharmacy relationships with vulnerable populations in their local settings may enhance uptake of the vaccine by these groups (CMOC12). Provision for privacy is an important user consideration. Pharmacy users expect private consultations to preserve their confidentiality (CMOC13).

For peer review only

Table 4 - Pharmacy Users (CMOCs 10-13)

**COMC 10 – Trust the pharmacist as reliable information source**[32 53 119-122]	**When pharmacy users receive information about the COVID-19 vaccine from their community pharmacy (C), they are more likely to get the vaccine (O), because they trust community pharmacy as a source of reliable, accurate information (M).**
	"When patients were educated about influenza, herpes zoster, and pneumococcal vaccines as a result of a pharmacist-driven intervention in community pharmacies, they were influenced to receive the vaccination." (USA)[119]<br><br>*"Being able to address the public enquiries with accurate up-to-date information about the local situation and the overall infection progress is the key to build trustful relationship with them at troubled times." (Macau)*[32]
**CMOC 11 – Trust the pharmacist to deliver responsive services**[7 18 23 26 34 37 43 51 54 59 63 70 84 85 123-135]	**When community pharmacies are trusted to make necessary service adaptations to ensure services are flexible, convenient and accessible (C), COVID-19 vaccine uptake is likely to be higher among pharmacy users (O) because the service is responsive to local needs (M).**
	"Pharmacists have always been the most accessible health care provider; this is especially true in the era of COVID-19….While other professionals have closed their doors to patients, community pharmacies remained open to the public despite stricter lockdown restrictions. As highly trusted healthcare clinicians, community pharmacists play a vital role in closing the gaps that are exacerbated by the additional strain on the system and reduced access to healthcare providers." (Canada)[51]

For peer review only

For peer review only - http://bmjopen.bmj.com/site/about/guidelines.xhtml

	"Pharmacy flu vaccination services complement those provided by general practitioners to help improve overall coverage and vaccination rates for patients in at-risk groups. These services are highly accessed by patients from all socio demographic areas, and seem to be particularly attractive to carers, frontline healthcare workers, and those of working age." (Great Britain)[135]
**CMOC 12 – Access culturally-sensitive services** *27 78 80 92 136*	**When community pharmacies leverage their community location and community-staff relationships (C), vulnerable populations, such as ethnic minorities, are more likely to use their services, including COVID-19 vaccination services (O), because they trust their local community pharmacies to provide culturally sensitive service. (M)**
	"Research continues to highlight that patients who are medically under-served have poorer inequitable access to health care due to them experiencing greater physical barriers to accessibility, encountering poorer patient-professional communication and are significantly disadvantaged where a service is not tailored to their unique needs or preferences." (Great Britain) [137]<br><br>*"I think over the years what's happened is nationally it's almost like everything has to be the same, which then doesn't work because it doesn't accommodate all the little variations…So we need to go back and in each individual pharmacy, gear it towards the population that it is meant to be meeting the needs of" (Pharmacist caring for ethnic minority community- Great Britain)*[137]

For peer review only - http://bmjopen.bmj.com/site/about/guidelines.xhtml

CMOC 13 – Receive private and confidential services[7 50 65 129 138-140]	When community pharmacies make provisions for privacy (C), pharmacy users are more likely to use their services (O), because they are reassured about confidentiality (M).
	"If there was something not right…the first thing I would do is make an appointment with a doctor. I wouldn't do and talk to somebody over a pharmacy counter." (Great Britain)[65]<br><br>*"Privacy, confidentiality and dignity are all vital elements of a trusting relationship between healthcare professionals and their patients…. In terms of privacy, the quality was perceived by the participants to include a confidential room that enabled private consultations." (Great Britain)*[7]

Public trust in community pharmacists is high—similar to doctors and nurses.[38] Trust between pharmacists and users can be leveraged to overcome scepticism about the COVID-19 vaccine. Providing reliable information about the disease and the vaccine, as pharmacies have done with other infections, can enhance public uptake of the COVID-19 vaccine.[113] In the USA, 90% of the US population lives within 5 miles of a community pharmacy. Given their convenience and accessibility, consumers have visited their community pharmacists 12 times more frequently than their GPs.[21] A UK study found that consumers who were eligible for a free influenza vaccine through their GPs were willing to pay for pharmacy service because of convenience and ease of access.[127] Established, trusting relationships are especially important for providing culturally sensitive services to marginalized and vulnerable communities.[129] Community pharmacists, often members of local communities are specially positioned to understand the culturally contextual factors that impact their pharmacy users.[89] Trusting relationships are founded on privacy, confidentiality and dignity,

For peer review only - http://bmjopen.bmj.com/site/about/guidelines.xhtml

and COVID-19, public health protocols and limited space must be considered—in order to maintain vital trust among pharmacy users.[65]

Discussion

This realist review sought to understand how community pharmacy can contribute to the public health agenda during the COVID-19 pandemic, particularly continuation of essential services and engagement in vaccination services.[115] As the COVID-19 vaccination service continues to evolve, our recommendations for decision makers highlight opportunities for community pharmacy to promote safe, efficient and effective service delivery.

Summary of key Findings

To optimise community pharmacy service during the pandemic, decision makers must endorse and articulate a clear role for these healthcare professionals. The public already endorses advanced roles for community pharmacy (e.g., vaccinations, minor ailment scheme), but public awareness depends on what decision makers do and say. Practical decision maker measures include adequate reimbursement to help cover the cost for time, staff and PPE (particularly for a sustainable long-term service); legal (including indemnity), regulatory coverage for advanced roles; and clear and consistent guidance for vaccination preparation and for adaptation of essential services. When given the opportunity, the permissions and resources to do so, community pharmacies have been able to adapt quickly to continue essential services and whenever possible, to offer critical advanced services. Historically, community pharmacies have significantly increased vaccination uptake (e.g., influenza vaccinations) given their accessibility and convenience and capacity to adapt to local needs for the general population and marginalized groups.

For peer review only - http://bmjopen.bmj.com/site/about/guidelines.xhtml

Compare to other similar or related studies discussing important differences in results

At the time of this realist review, there were no similar reviews on community pharmacy roles with respect to COVID-19 vaccinations. COVID-19 represents an unprecedented situation with limited direct evidence to guide decision-making. However, realist approaches engage with a wider evidence base, including research on past pandemics (e.g., SARS)[60 141 142], mass vaccination campaigns (e.g., influenza)[143], and community pharmacies' capacity pre-COVID-19 to deliver essential and advanced services.[19 144 145] Key factors previously include a lack of leadership, a lack of guidance and an increasing reliance on professional judgement and experience.[60] Research from the UK and other economically developed countries supports the 13 CMOCs and decision maker recommendations in this review (see tables 2-4 and table 5). However, limitations continue to surround the direct policy relevance of much of the community pharmacy evidence base.[146]

Study strengths and limitations

The realist approach uses diverse data, including grey literature. This feature is especially important given a novel and rapidly evolving topic area, such as COVID-19. Multiple researchers with subject matter expertise participated in screening the literature and extracting and coding data, which maximised opportunities to discuss and debate the plausibility of the inferences made. The CMOCs were developed and refined through regular discussions within a team with varied academic and clinical backgrounds. Professional and public stakeholder consultation further refined the CMOCs.

All rapid reviews operationalise coverage versus expediency. Other sources of evidence could have informed the review; however, potential gaps were mitigated by stakeholder engagement and expertise within the team. The evidence supporting the CMOCs was based on available time for document review, and during the review period, research, directives and policy related to COVID-19

For peer review only - http://bmjopen.bmj.com/site/about/guidelines.xhtml

vaccination rapidly evolved. The programme theory and its CMOCs, however, are expressed in such a way that they can be further confirmed, refuted or refined in the future using additional data.

For this rapid review, given our short timeline of six months, we initially decided to focus on community pharmacy roles and pandemic response in middle to high-income countries. Whilst we identified data from higher-income countries such as Canada, USA, Australia and France, there was very little published from middle-income countries. As such, it is likely that our findings and recommendations are most applicable to high-income countries, although, the lessons learned from high-income countries can serve as an initial reference point for best practice for other countries.

Our recommendations can be viewed as ''how tos" for other countries to consider within their own cultural and healthcare environments. A number of European and North American countries have introduced legislative changes to extend the role of community pharmacy and thus reduce pressure on other parts of their healthcare systems.[145 147] Our findings can be used by community pharmacy and decision makers, from countries other than the UK, when mapping out similar services particularly in the context of changes in the legislation.

Meaning of the study: possible explanations and implications for decision makers

Although there are multiple actors involved in pandemic response, for brevity, recommendations (Table 5) are directed towards decision makers who possess the formal authority to implement the recommendations.

Table 5 - Recommendations for Decision Makers to Increase Community Pharmacy Engagement in Pandemic Response (for further details check: https://publications.aston.ac.uk/id/eprint/42310/1/Guidance_for_Policy_Makers_on_the_role_of_Community_Pharmacy_in_COVID.pdf).

For peer review only - http://bmjopen.bmj.com/site/about/guidelines.xhtml

Recommendations	Derived from CMOC
Articulate a clear public health agenda role for community pharmacy (e.g., COVID-19 testing and vaccination)	1
Ensure pharmacy regulations for advanced roles, such as novel vaccine administration, are in place to legally protect community pharmacists and their teams	2
Involve local community pharmacies in policy and service specification development	3
Provide timely guidance with sufficient details for community pharmacies to quickly adapt to local needs	4
Provide adequate funding and reimbursement for community pharmacy services to deliver COVID-19 vaccines	5
Equip community pharmacies with the necessary permissions to manage and adapt essential services	6
Ensure community pharmacies have the means to adequately protect the health of themselves, their staff and pharmacy users	8
Facilitate collaboration and coordination of COVID-19 vaccination services across providers (e.g., GPs, community pharmacies) and systems (e.g., IT)	9

As the CMOCs were refined, 'tensions' were uncovered with implications for decision makers, particularly: community pharmacists as healthcare professionals versus retailers[43 106 145 148]; community pharmacies' capacity to remain financially viable while managing essential and advanced services.[19 149 150] and; pharmacists' capacity to provide off-site services while maintaining physical premises.[151] These tensions stem from lack of awareness of community pharmacists as healthcare professionals.

For peer review only - http://bmjopen.bmj.com/site/about/guidelines.xhtml

Along the continuum of healthcare from community to hospital, community pharmacy is often the first point of contact for the public. Community pharmacies contribute to primary care services through essential services (e.g., medication dispensing) and expanded roles (e.g., vaccinations),[115 148] often decreasing workload pressures on other providers, such as GPs.[152] Public surveys demonstrate high levels of satisfaction with community pharmacy services, and vaccination uptake is increased when pharmacies deliver these services.[150] Nevertheless, lack of public and decision maker awareness of community pharmacy primary care roles has slowed uptake and integration of these services.[121] A recent UK public survey found that community pharmacies are seen as “a medicine supply shop by 48.3% of people, as a place to purchase medicines by 22%, and a place to purchase non-medicinal products by 17.7%.” [28]

In Ireland and Canada community pharmacies are an integral part of national vaccination campaigns.[150] In both instances, community pharmacists participate in vaccination planning, pharmacy regulators provide clear guidance on vaccination management, and vaccinations are equitably refunded through public health systems. The removal of barriers, such as economic pressures on pharmacies has resulted in impressive national vaccine uptake, even in large countries, such as Canada.[150] Globally, there is an emerging trend for governments, health insurance companies and consumers to remunerate community pharmacies for services that contribute to improved health outcomes.[151]

Community pharmacies tend to be defined as premises where medications are dispensed, which compounds the confusion (by the public and decision makers) of professional primary care services versus retailers.[151] Instead, pharmacies should be defined with respect to actions or services that require specialised health knowledge to optimise health outcomes. For example, a retail approach to over-the-counter (OTC) medications is to permit consumers to make their own choices, similar to

For peer review only - http://bmjopen.bmj.com/site/about/guidelines.xhtml

supermarket choices. Pharmacy input for into OTC purchases could potential decrease adverse medication interactions or unnecessary allergic reactions.[148] The 'value-add' of community pharmacies, evidence-informed engagement with consumers, can decrease morbidity and mortality outcomes and increase medication regimen adherence.[151 153]

Although community pharmacists can provide professional services off premises (e.g., immunizations in community and religious centres), they need to maintain their physical premises and staff for financial reasons. In addition, pharmacy users seek out community pharmacy services due to their accessibility and convenience, and without a physical space to engage regularly with pharmacy users, trust-building between pharmacists and users is compromised.[154]

As evidenced by public media, many countries, including the UK are declaring service specifications for COVID-19 vaccinations. Community pharmacists can potentially provide vaccination services in two locations.[155] They can collaborate with GPs in Primary Care Networks (PCN) to support PCN vaccination sites. Alternatively, they can provide a COVID-19 vaccination service from their premises if they meet service specifications. The described tensions create difficult choices for pharmacists that can be ameliorated through the decision maker recommendations in Table 5.

Unanswered questions and future research

A future realist evaluation involving primary data collection from key actors will inform refinement of the programme theory, CMOCs and decision maker recommendations. This empirical research will address the tensions identified above. Future research will also address issues identified through the stakeholder groups, such as vaccination hesitancy, outside the scope of this review. While this work touched upon unique issues for marginalised populations including ethnic minorities, those of lower socioeconomic status and those with disabilities, further exploration is needed.

For peer review only - http://bmjopen.bmj.com/site/about/guidelines.xhtml

We had initially focused the review on community pharmacy roles and pandemic response in middle to high-income countries, but found limited data in middle-income countries. The findings are thus more likely to be applicable to high-income countries but may serve to inform practice elsewhere. Empirical research, using for example a realist evaluation approach, should be conducted to extend our findings on the role of community pharmacy in COVID vaccination programmes to middle to low income countries.

Conclusion

The COVID-19 pandemic is a worldwide health emergency. Vaccination is key to combatting the pandemic. The role of community pharmacy may be both short and long term; with a potential need for regular annual vaccines. This rapid realist review offers recommendations for decision makers to enable community pharmacy to play a key role, both during these unprecedented times and into the future.

For peer review only

Acknowledgement:

We acknowledge the contribution from professional and member of the public key stakeholder.

Research Ethics Approval

This research did not involve human participants and therefore ethical approval was not required.

Funders

Jointly funded by UKRI and NIHR COV0176. The views expressed are those of the author(s) and not necessarily those of the NIHR, UKRI or the Department of Health and Social Care.

For peer review only

Contributorship statement

IM conceived and led the project and had overall responsibility for PERISCOPE. All team members developed develop the embryonic programme theory. AB conducted the searches. AB and GW advised on realist methods. GW also advised on primary care implications and provided methodological oversight. EY screened documents at title, abstract and full-text with support from AB and MM. EY, MM and JB coded relevant data into NVivo. EY, MM and JB conducted the data analysis/synthesis. HZ and AH advised on pharmacy aspects and policy implications. TK led PPI. TK and HZ advised on implications in ethnic minorities. All team members contributed drafting the final report for publication and approved the final draft for submission.

Data Sharing Statement

Data is available upon reasonable request to the lead author.

Competing Interests

Geoff Wong is Deputy Chair of the NIHR HTA Prioritisation Committee: Integrated Community Health and Social Care (A) and Member of the NIHR HTA Prioritisation Committee: Integrated Community Health and Social Care (A) Methods Group.

Andrew Booth is a member of the National Institute for Health Research Health Services and Delivery Research Funding Board, the National Institute for Health Research Evidence Synthesis Programme Advisory Group and the National Institute for Health Research School for Social Care Research Commissioning Panel.

For peer review only

For peer review only - http://bmjopen.bmj.com/site/about/guidelines.xhtml

References

1. Parkhurst C, Singh Purewal G, Donyai P. Community Pharmacy and COVID-19—The Unsung Heroes on Our High Streets. *Journal of Patient Experience* 2020;7(3):282-84. doi: 10.1177/2374373520927638

2. Maidment I, Lawson S, Wong G, et al. Towards an understanding of the burdens of medication management affecting older people: the MEMORABLE realist synthesis. *BMC geriatrics* 2020;20(1):183. doi: 10.1186/s12877-020-01568-x [published Online First: 2020/06/06]

3. Barry HE, Hughes CM. Managing medicines in the time of COVID-19: implications for community-dwelling people with dementia. *International Journal of Clinical Pharmacy* 2020. doi: 10.1007/s11096-020-01116-y

4. Wickware C. Pharmacies' dispensing increases by up to a third as a result of COVID-19, survey finds. . *Pharm J* 2020:PJ April 2020 online. doi: 10.1211/pj.2020.20207917

5. Pharmaceutical Services Negotiating Committee. Flu Vaccination Service 2020 [Available from: https://psnc.org.uk/services-commissioning/advanced-services/flu-vaccination-service. Accessed 12/11/20]

6. Pharmaceutical Services Negotiating Committee. Vaccination Services 2020 [Available from: https://psnc.org.uk/services-commissioning/locally-commissioned-services/vaccination-services. Accessed 08/12/2020]

7. Rendell T. Community pharmacist-led influenza vaccination: a service evaluation. *The Pharmaceutical Journal* 2020. doi: 10.1211/pj.2020.20208383

8. Public Health England. Beyond the data: Understanding the impact of COVID-19 on BAME groups. London: Public Health England, 2020. [Available from: https://assets.publishing.service.gov.uk/government/uploads/system/uploads/attachment_data/file/892376/COVID_stakeholder_engagement_synthesis_beyond_the_data.pdf. Accessed 08/12/20]

For peer review only - http://bmjopen.bmj.com/site/about/guidelines.xhtml

9. Patel M. We must tear down the barriers to equality for BAME communities highlighted by COVID-19. *Pharmaceutical Journal* 2020. doi: 10.1211/PJ.2020.20208060

10. Robinson J. Ten things pharmacists should know about COVID-19 vaccines. *The Pharmaceutical Journal* 2020. doi: 10.1211/pj.2020.20208429

11. Wong G, Brennan N, Mattick K, et al. Interventions to improve antimicrobial prescribing of doctors in training: the IMPACT (IMProving Antimicrobial presCribing of doctors in Training) realist review. *BMJ Open* 2015;5(10):e009059. doi: 10.1136/bmjopen-2015-009059

12. Maidment ID, Lawson S, Wong G, et al. Health Services and Delivery Research. Medication management in older people: the MEMORABLE realist synthesis. Southampton (UK): NIHR Journals Library 2020. doi: 10.3310/hsdr08260

13. Maidment I, Young E, Wong G, et al. A rapid realist review of community pharmacy support for the public health agenda during the COVID-19 pandemic and future health emergencies. PROSPERO 2020 CRD42020194078, 2020. doi: 10.1101/2021.02.01.21250765

14. Wong G, Greenhalgh T, Westhorp G, et al. Development of methodological guidance, publication standards and training materials for realist and meta-narrative reviews: the RAMESES (Realist And Meta-narrative Evidence Syntheses – Evolving Standards) project. *Health Services and Delivery Research* 2014;2(30):1-252. doi: 10.3310/hsdr02300

15. Pharmacies across the UK close 'intermittently' to cope with increasing pressures from COVID-19. *The Pharmaceutical Journal* 2020. doi: 10.1211/pj.2020.20207844

16. Cadogan CA, Hughes CM. On the frontline against COVID-19: Community pharmacists' contribution during a public health crisis. *Research in Social and Administrative Pharmacy* 2021;17(1):2032-35. doi: 10.1016/j.sapharm.2020.03.015

17. Royal Pharmaceutical Society. RPS guidance on ethical, professional decision making in the COVID-19 Pandemic. London: Royal Pharmaceutical Society, 2020. [Available from: https://www.rpharms.com/resources/pharmacy-guides/coronavirus-covid-19/coronavirus-information-for-pharmacists/ethical-decision-making. Accessed 12/12/20]

For peer review only

For peer review only - http://bmjopen.bmj.com/site/about/guidelines.xhtml

18. Pharmaceutical Services Negotiating Committee. PSNC Briefing 023/20: Early guidance on the 2020/21 Seasonal Influenza Vaccination Advanced Service 2020 [Available from: https://psnc.org.uk/services-commissioning/psnc-briefings-services-and-commissioning/psnc-briefing-023-20-early-guidance-on-the-2020-21-seasonal-influenza-vaccination-advanced-service. Accessed 08/12/20]

19. Hattingh L, Sim TF, Sunderland B, et al. Successful implementation and provision of enhanced and extended pharmacy services. *Research in Social and Administrative Pharmacy* 2020;16(4):464-74. doi: 10.1016/j.sapharm.2019.06.015 [published Online First: 2019/07/06]

20. Greenhalgh T, Wong G, Westhorp G, et al. Retroduction in realist evaluation. [Internet]. The RAMESES Project 2017. [Available from: http://www.ramesesproject.org/media/RAMESES_II_Retroduction.pdf. Accessed 12/12/20]

21. Wong G. Data Gathering in Realist Reviews: Looking for needles in haystacks. Doing Realist Research: SAGE Publications Ltd, 2018:131-46. doi: 10.4135/9781526451729.n9

22. Long N. Development sociology: actor perspectives. London: Routledge 2003.

23. National Pharmacy Association. Public see pharmacies as key to the future of healthcare in wake of pandemic 2020 [Available from: https://www.npa.co.uk/news-and-events/news-item/public-see-pharmacies-as-key-to-the-future-of-healthcare-in-wake-of-pandemic. Accessed 14/01/2021].

24. Royal Pharmaceutical Society. RPS Statement on the role of pharmacists in the COVID-19 Vaccination Programme London: Royal Pharmaceutical Society; 2020 [Available from: https://www.rpharms.com/Portals/0/FINAL_Statement%20on%20COVID-19%20Vaccination_Dec%202020_.pdf?ver=2020-12-02-143304-017. Accessed 14/01/2021].

25. Strand MA, Bratberg J, Eukel H, et al. Community Pharmacists' Contributions to Disease Management During the COVID-19 Pandemic. *Preventing Chronic Disease* 2020;17. doi: 10.5888/pcd17.200317

For peer review only

26. Elbeddini A, Prabaharan T, Almasalkhi S, et al. Pharmacists and COVID-19. *Journal of Pharmaceutical Policy and Practice* 2020;13(1). doi: 10.1186/s40545-020-00241-3

27. Kirkdale CL, Nebout G, Taitel M, et al. Implementation of flu vaccination in community pharmacies: Understanding the barriers and enablers. *Annales Pharmaceutiques Françaises* 2017;75(1):9-16. doi: 10.1016/j.pharma.2016.08.006

28. Hindi AMK, Jacobs S, Schafheutle EI. Solidarity or dissonance? A systematic review of pharmacist and GP views on community pharmacy services in the UK. *Health & Social Care in the Community* 2018;27(3):565-98. doi: 10.1111/hsc.12618

29. Deslandes R, Evans A, Baker S, et al. Community pharmacists at the heart of public health: A longitudinal evaluation of the community pharmacy influenza vaccination service. *Research in Social and Administrative Pharmacy* 2020;16(4):497-502. doi: 10.1016/j.sapharm.2019.06.016

30. Sousa Pinto G, Hung M, Okoya F, et al. FIP's response to the COVID-19 pandemic: Global pharmacy rises to the challenge. *Research in Social and Administrative Pharmacy* 2021;17(1):1929-33. doi: 10.1016/j.sapharm.2020.07.004

31. Gregory PAM, Austin Z. COVID-19: How did community pharmacies get through the first wave? *Canadian Pharmacists Journal / Revue des Pharmaciens du Canada* 2020;153(5):243-51. doi: 10.1177/1715163520945741

32. Ung COL. Community pharmacist in public health emergencies: Quick to action against the coronavirus 2019-nCoV outbreak. *Research in Social and Administrative Pharmacy* 2020;16(4):583-86. doi: 10.1016/j.sapharm.2020.02.003

33. Burson RC, Buttenheim AM, Armstrong A, et al. Community pharmacies as sites of adult vaccination: A systematic review. *Human vaccines & immunotherapeutics* 2016;12(12):3146-59. doi: 10.1080/21645515.2016.1215393 [published Online First: 08/15]

For peer review only

34. Nadeem MF, Samanta S, Mustafa F. Is the paradigm of community pharmacy practice expected to shift due to COVID-19? *Research in Social and Administrative Pharmacy* 2021;17(1):2046-48. doi: 10.1016/j.sapharm.2020.05.021

35. Francis M, Hinchliffe A, Wales N. Vaccination services through community pharmacy. *Public Health Wales NHS Trust, Wales: NHS Wales* 2010. [Available from: http://www2.nphs.wales.nhs.uk:8080/HealthServiceQDTDocs.nsf/public/CBDDC3C0BE9449398025793B00341A6F/$file/Vaccination%20services%20through%20community%20pharmacy%20v1a.pdf. Accessed 08/12/20]

36. Clews G. Pharmacists able to administer unlicensed COVID-19 vaccines following legislation changes. *The Pharmaceutical Journal* 2020;305. doi: 10.1211/PJ.2020.20208453

37. Welsh Government, Department of Health N. I., Scottish Government, et al. Supporting pharmacists, pharmacy technicians and pharmacy teams throughout the second COVID-19 wave, 2020. [Available from: https://www.pharmacyregulation.org/news/joint-letter-supporting-pharmacy-professionals-and-teams-through-second-covid-19-wave. Accessed 12/12/20]

38. Wickware C. NHS England to choose 'selected' number of pharmacies to deliver at least 1,000 COVID-19 vaccines per week. *The Pharmaceutical Journal* 2020. doi: 10.1211/PJ.2020.20208608

39. Dyer C. Covid-19: Government poised to amend regulations to allow use of unlicensed vaccine. *The BMJ* 2020. doi: 10.1136/bmj.m3757

40. Cooper RJ. Pestle and mortal: the demise of community pharmacy in the UK. *International Journal of Pharmacy Practice* 2020;28(3):205-06. doi: 10.1111/ijpp.12635

41. Robinson J. Health professionals delivering unlicensed vaccines need government protection, say health bodies. *The Pharmaceutical Journal* 2020. doi: 10.1211/PJ.2020.20208381

For peer review only - http://bmjopen.bmj.com/site/about/guidelines.xhtml

42. Warner JG, Portlock J, Smith J, et al. Increasing seasonal influenza vaccination uptake using community pharmacies: experience from the Isle of Wight, England. *International Journal of Pharmacy Practice* 2013;21(6):362-67. doi: 10.1111/ijpp.12037

43. Hindi AMK, Schafheutle EI, Jacobs S. Community pharmacy integration within the primary care pathway for people with long-term conditions: a focus group study of patients', pharmacists' and GPs' experiences and expectations. *BMC Fam Pract* 2019;20(1):26-26. doi: 10.1186/s12875-019-0912-0

44. Pharmacy Voice, The Pharmaceutical Services Negotiating Committee, The Royal Pharmaceutical Society, et al. Consultation response: UK Influenza Pandemic Preparedness Strategy 2011. 2011

45. Bauman JL. Hero clinical pharmacists and the COVID-19 pandemic: Overworked and overlooked. *JACCP JAm Coll Clin Pharm* 2020. doi: 10.1002/jac5.1246

46. Stone JK, Pate AN. The Impact of COVID-19 Through the Eyes of a Fourth-Year Pharmacy Student. *American Journal of Pharmaceutical Education* 2020;84(6):ajpe8146. doi: 10.5688/ajpe8146

47. Royal College of General Practitioners. Delivering Mass Vaccinations During COVID-19 A Logistical Guide for General Practice. London: Royal College of General Practitioners, 2020. [Available from: https://www.lmc.org.uk/visageimages/Covid-19/RCGPMassVaccinationattimeofcovidV3.1.pdf. Accessed 08.12.20]

48. NHS England and NHS Improvement coronavirus. Urgent preparing for General Practice to contribute to a potential COVID-19 Vaccination Programme. In: Service NH, ed. London, 2020. [Available from: https://www.england.nhs.uk/coronavirus/covid-19-vaccination-programme/primary-care-guidance/preparing-for-gp-to-contribute-to-a-potential-covid-19-vaccination-programme. Accessed 08/12/20]

49. Wickware, C. NHS England to choose 'selected' number of pharmacies to deliver at least 1,000 COVID-19 vaccines per week. *The Pharmaceutical Journal* 2020. doi: 10.1211/pj.2020.20208608

For peer review only

50. Koster ES, Philbert D, Bouvy ML. Impact of the COVID-19 epidemic on the provision of pharmaceutical care in community pharmacies. *Research in Social and Administrative Pharmacy* 2020. doi: 10.1016/j.sapharm.2020.07.001

51. Elbeddini A, Wen CX, Tayefehchamani Y, et al. Mental health issues impacting pharmacists during COVID-19. *Journal of Pharmaceutical Policy and Practice* 2020;13(1). doi: 10.1186/s40545-020-00252-0

52. Bukhari N, Rasheed H, Nayyer B, et al. Pharmacists at the frontline beating the COVID-19 pandemic. *Journal of Pharmaceutical Policy and Practice* 2020;13 (1). doi: 10.1186/s40545-020-00210-w

53. Eidt, M. Pharmacists on the frontline. *Canadian Pharmacists Journal* 2020;153(4):186-90. doi: 10.1177/1715163520930191

54. Farrell B, Tsuyuki RT. Pharmacists step up to the challenge of COVID-19. *Canadian Pharmacists Journal* 2020;153(4):185. doi: 10.1177/1715163520930705

55. Bragazzi NL, Mansour M, Bonsignore A, et al. The Role of Hospital and Community Pharmacists in the Management of COVID-19: Towards an Expanded Definition of the Roles, Responsibilities, and Duties of the Pharmacist. *Pharmacy (Basel, Switzerland)* 2020. doi: 10.3390/pharmacy8030140

56. Chan AHY, Rutter V, Ashiru-Oredope D, et al. Together we unite: The role of the Commonwealth in achieving universal health coverage through pharmaceutical care amidst the COVID-19 pandemic. *Journal of Pharmaceutical Policy and Practice* 2020;13(1). doi: 10.1186/s40545-020-00214-6

57. Moore A. Pharmacists in one county say working with GP practices during the COVID-19 pandemic has been ‘difficult’. *The Pharmaceutical Journal* 2020. doi: 10.1211/PJ.2020.20208556

58. Hoti K, Jakupi A, Hetemi D, et al. Provision of community pharmacy services during COVID-19 pandemic: a cross sectional study of community pharmacists' experiences with preventative

For peer review only

measures and sources of information. Int J Clin Pharm 2020. doi: 10.1007/s11096-020-01078-1

59. NHS England and NHS Improvement coronavirus. Urgent preparing for Community Pharmacy to contribute to a potential COVID-19 Vaccination Programme. In: Service NH, ed. London, 2020. [Available from: https://www.england.nhs.uk/coronavirus/covid-19-vaccination-programme/primary-care-guidance/preparing-for-community-pharmacy-to-contribute-to-a-potential-covid-19-vaccination-programme. Accessed 08/12/20]

60. Austin Z, Martin JC, Gregory PAM. Pharmacy practice in times of civil crisis: The experience of SARS and "the blackout" in Ontario, Canada. *Research in Social and Administrative Pharmacy* 2007;3(3):320-35. doi: 10.1016/j.sapharm.2006.09.001

61. Chahine EB. The Role of Pharmacists in the Fight Against COVID-19. *Sr Care Pharm* 2020;35(7):286-91. doi: 10.4140/TCP.n.2020.286

62. Jubraj B. Remote consultations: how pharmacy teams can practise them successfully: pharmaceutical-journal.com 2020. [Available from: https://pharmaceutical-journal.com/article/ld/remote-consultations-how-pharmacy-teams-can-practise-them-successfully. Accessed 12/12/20]

63. Royal Pharmaceutical Society. All-Party Pharmacy Group warns of pressures on pharmacies London: Royal Pharmaceutical Society 2020 [Available from: https://www.rpharms.com/about-us/news/details/All-Party-Pharmacy-Group-warns-of-pressures-on-pharmacies#:~:text=The%20All%2DParty%20Pharmacy%20Group,continued%20pressures%20facing%20community%20pharmacies. Accessed 14/01/2021]

64. Barnes E, Bullock A, Allan M, et al. Community pharmacists' opinions on skill-mix and delegation in England. *International Journal of Pharmacy Practice* 2017;26(5):398-406. doi: 10.1111/ijpp.12419

For peer review only

65. Saramunee K, Krska J, Mackridge A, et al. How to enhance public health service utilization in community pharmacy?: General public and health providers' perspectives. *Research in Social and Administrative Pharmacy* 2014;10(2):272-84. doi: 10.1016/j.sapharm.2012.05.006

66. Houle SKD, Grindrod KA, Chatterley T, et al. Paying pharmacists for patient care: A systematic review of remunerated pharmacy clinical care services. *Can Pharm J (Ott)* 2014;147(4):209-32. doi: 10.1177/1715163514536678

67. Burns C. Pharmacy staff in Scotland to receive £500 bonus in recognition of COVID-19 service. *the Pharmaceutical Journal* 2020 doi: 10.1211/PJ.2020.20208614 [published Online First: 12/2020]

68. Evans AM, Wood FC, Carter B. National community pharmacy NHS influenza vaccination service in Wales: a primary care mixed methods study. *British Journal of General Practice* 2016;66(645):e248-e57. doi: 10.3399/bjgp16x684349

69. Moore A. No going back: how the pandemic is changing community pharmacy. *The Pharmaceutical Journal* 2020;305. doi: 10.1211/PJ.2020.20208309

70. Merks P, Jakubowska M, Drelich E, et al. The legal extension of the role of pharmacists in light of the COVID-19 global pandemic. *Research in Social and Administrative Pharmacy* 2020 doi: 10.1016/j.sapharm.2020.05.033

71. Zaidi STR, Hasan SS. Personal protective practices and pharmacy services delivery by community pharmacists during COVID-19 pandemic: Results from a national survey. *Research in Social and Administrative Pharmacy* 2020. doi: 10.1016/j.sapharm.2020.07.006

72. Anon. Pharmacies must be at the forefront of the COVID-19 vaccination campaign. *The Pharmaceutical Journal* 2020;305. doi: 10.1211/PJ.2020.20208546

73. Robinson J. England has lowest number of community pharmacies in five years, figures show. *The Pharmaceutical Journal* 2020. doi: 10.1211/PJ.2020.20208569

74. Wickware C. Pharmacies to be paid 50p more per flu vaccination in 2020/2021. *The Pharmaceutical Journal* 2020. doi: 10.1211/PJ.2020.20208362

For peer review only - http://bmjopen.bmj.com/site/about/guidelines.xhtml

75. Doyle-Price J. Pharmacies have an important role in this pandemic – but they need help to continue saving lives. *The Telegraph* 2020. [Available from: https://www.telegraph.co.uk/global-health/science-and-disease/pharmacies-have-crucial-place-communities-need-help-continue. Accessed 12/12/20]

76. Robinson J. Up to 85% of community pharmacies could be in financial deficit by 2024, economic analysis concludes. *The Pharmaceutical Journal* 2020. doi: 10.1211/PJ.2020.20208332

77. Wilkinson E, Connelly D. Is pharmacy prepared for a resurgence of COVID-19? *The Pharmaceutical Journal* 2020;305. doi: 10.1211/PJ.2020.20208340

78. Hayden JC, Parkin R. The challenges of COVID-19 for community pharmacists and opportunities for the future. *Irish Journal of Psychological Medicine* 2020;37(3):198-203. doi: 10.1017/ipm.2020.52

79. Mercadante AR, Yokota M, Hwang A, et al. Choosing Evolution over Extinction: Integrating Direct Patient Care Services and Value-Based Payment Models into the Community-Based Pharmacy Setting. *Pharmacy* 2020;8(3):128. doi: 10.3390/pharmacy8030128

80. Stewart K, Barai R, Priarities N. How to keep your community pharmacy running during the COVID-19 pandemic 2020. [Available from: https://pharmaceutical-journal.com/article/ld/how-to-keep-your-community-pharmacy-running-during-the-covid-19-pandemic. Accessed 24/02/21]

81. Hussain R, Dawoud DM, Babar Z-U-D. Drive-thru pharmacy services: A way forward to combat COVID-19 pandemic. *Research in Social and Administrative Pharmacy* 2021;17(1):1920-24. doi: 10.1016/j.sapharm.2020.07.015

82. Broughel J, Yatsyshina Y. Relax Pharmacy Regulations to Help with COVID-19 Testing and Treatment. *SSRN Electronic Journal* 2020. doi: 10.2139/ssrn.3564463

83. Pritchard RI, Huff J, Scheinberg N. Impact of Regulatory Changes on Pharmacist Delivered Telehealth During the COVID-19 Pandemic. *Journal of the American Pharmacists Association* 2020;60(6):e76-e79. doi: 10.1016/j.japh.2020.06.004

For peer review only - http://bmjopen.bmj.com/site/about/guidelines.xhtml

84. Lynch M, Leary A. COVID-19 related regulatory change for pharmacists – The case for its retention post the pandemic. *Research in Social and Administrative Pharmacy* 2020. doi: 10.1016/j.sapharm.2020.07.037

85. Erku DA, Belachew SA, Abrha S, et al. When fear and misinformation go viral: Pharmacists' role in deterring medication misinformation during the 'infodemic' surrounding COVID-19. *Research in Social and Administrative Pharmacy* 2020. doi: 10.1016/j.sapharm.2020.04.032

86. Andrawis M, Carmichael J, Collins CD, et al. Improving patient care and demonstrating value during a global pandemic: Recommendations from leaders of the Pharmacy Accountability Measures Work Group. *American journal of health-system pharmacy : AJHP : official journal of the American Society of Health-System Pharmacists* 2020. doi: 10.1093/ajhp/zxaa291

87. Kawaguchi-Suzuki M, Nagai N, Akonoghrere RO. COVID-19 Pandemic Challenges and Lessons Learned by Pharmacy Educators Around the Globe. *American Journal of Pharmaceutical Education* 2020;84(8) [published Online First: 2020 Aug 1]. doi: 10.5688/ajpe8197

88. General Pharmaceutical Council. Rising to the challenge [blog] 2020 [Available from: https://www.pharmacyregulation.org/regulate/blog/rising-challenge. Accessed 14/01/2020 2020]

89. Robinson J. Government consults on allowing pharmacy technicians to administer COVID-19 and flu vaccines. *The Pharmaceutical Journal* 2020. doi: 10.1211/PJ.2020.20208311

90. Nessel, J. Pharmacy Technicians Rise to Meet Patient Needs During Coronavirus Pandemic. *Pharmacy Times* 2020. Available from: https://www.pharmacytimes.com/view/pharmacy-technicians-rise-to-meet-patient-needs-during-coronavirus-pandemic. Accessed 08.12.20]

91. Green TC, Bratberg J, Finnell DS. Opioid use disorder and the COVID 19 pandemic: A call to sustain regulatory easements and further expand access to treatment. *Subst Abus* 2020;41(2):147-49. doi: 10.1080/08897077.2020.1752351

92. Carico R. Pharmacists continue to protect communities during COVID-19. *Journal of the American Pharmacists Association* 2020;60(5):660-61. doi: 10.1016/j.japh.2020.08.009

For peer review only

For peer review only - http://bmjopen.bmj.com/site/about/guidelines.xhtml

93. Bond C, Blenkinsopp A, Inch J, et al. The effect of the new community pharmacy contract on the community pharmacy workforce. *London: Pharmacy Practice Research Trust* 2008;8:9. Available from: https://pharmacyresearchuk.org/wp-content/uploads/2012/11/The_effect_of_the_new_community_pharmacy_contract_on_the_community_pharmacy_workforce.pdf. Accessed 08/12/20]

94. Melton BL, Lai Z. Review of community pharmacy services: what is being performed, and where are the opportunities for improvement? *Integr Pharm Res Pract* 2017;6:79-89. doi: 10.2147/IPRP.S107612

95. Wilson CG, Ramage M, Fagan EB. A Primary Care Response to COVID-19 for Patients with an Opioid Use Disorder. *J Rural Health* 2020. doi: 10.1111/jrh.12438

96. Kostev K, Lauterbach S. Panic buying or good adherence? Increased pharmacy purchases of drugs from wholesalers in the last week prior to Covid-19 lockdown. *J Psychiatr Res* 2020;130:19-21. doi: 10.1016/j.jpsychires.2020.07.005

97. Bell JS, Reynolds L, Freeman C, et al. Strategies to promote access to medications during the COVID-19 pandemic. *Aust J Gen Pract* 2020;49(8):530-32. doi: 10.31128/AJGP-04-20-5390

98. Romano S, Galante H, Figueira D, et al. Time-trend analysis of medicine sales and shortages during COVID-19 outbreak: Data from community pharmacies. *Research in Social and Administrative Pharmacy* 2020. doi: 10.1016/j.sapharm.2020.05.024

99. Gross AE, MacDougall C. Roles of the clinical pharmacist during the COVID-19 pandemic. *JACCP JAm Coll Clin Pharm* 2020;3(3):564-66. doi: 10.1002/jac5.1231

100. Lapeyre-Mestre M, Boucher A, Daveluy A, et al. Addictovigilance contribution during COVID-19 epidemic and lockdown in France. *Therapies* 2020. doi: 10.1016/j.therap.2020.06.006

101. Alderman C. Pharmacy Services and the Novel Coronavirus. *The Senior Care Pharmacist* 2020;35 (4):146-47. doi: 10.4140/TCP.n.2020.146

For peer review only - http://bmjopen.bmj.com/site/about/guidelines.xhtml

102. Hasan SS, Kow CS, Zaidi STR. Social distancing and the use of PPE by community pharmacy personnel: Does evidence support these measures? *Research in Social and Administrative Pharmacy* 2020;17(2):456-9. doi: 10.1016/j.sapharm.2020.04.033

103. Thomas CEL, Phipps DL, Ashcroft DM. When procedures meet practice in community pharmacies: qualitative insights from pharmacists and pharmacy support staff. *BMJ open* 2016;6(6):e010851-e51. doi: 10.1136/bmjopen-2015-010851

104. NHS England and NHS Improvement coronavirus. Standard operating procedure: COVID-19 local vaccination services deployment in community settings. London: National Health Service 2020. Available from: https://www.england.nhs.uk/coronavirus/publication/standard-operating-procedure-covid-19-local-vaccination-services-deployment-in-community-settings. Accessed 08.12.20]

105. Cabas P, Di Bella S, Giuffrè M, et al. Community pharmacists' exposure to COVID-19. *Research in Social and Administrative Pharmacy* 2021;17(1):1882-87. doi: 10.1016/j.sapharm.2020.05.020

106. Sum ZZ, Ow CJW. Community pharmacy response to infection control during COVID-19. A cross-sectional survey. *Res Soc Adm Pharm* 2020;17(1):1845-52. doi: 10.1016/j.sapharm.2020.06.014

107. Al-Quteimat OM, Amer AM. SARS-CoV-2 outbreak: How can pharmacists help? *Research in Social and Administrative Pharmacy* 2021;17(2):480-82. doi: 10.1016/j.sapharm.2020.03.018 [published Online First: 2020 Mar 26]

108. Dawoud D. Emerging from the other end: Key measures for a successful COVID-19 lockdown exit strategy and the potential contribution of pharmacists. *Research in Social and Administrative Pharmacy* 2020. doi: 10.1016/j.sapharm.2020.05.011

109. Weir NM, Newham R, Bennie M. A literature review of human factors and ergonomics within the pharmacy dispensing process. *Research in Social and Administrative Pharmacy* 2020;16(5):637-45. doi: 10.1016/j.sapharm.2019.08.029

For peer review only - http://bmjopen.bmj.com/site/about/guidelines.xhtml

787 110. Maine LL. An Unparalleled Spirit of Collaboration Amid the COVID-19 Pandemic. *Am J Pharm*
788 *Educ* 2020;84(6):ajpe8155-ajpe55. doi: 10.5688/ajpe8155
789 111. Dowden A. Will Serious Shortage Protocols help ease drug shortages? *Prescriber* 2020;31(5):29-
790 31. doi: 10.1002/psb.1844
791 112. NHS England, NHS Improvement coronavirus. Novel coronavirus (COVID-19) standard operating
792 procedure-Community Pharmacy. [Available from: https://www.england. nhs. uk/wp-
793 content/uploads/2020/02/20200305-COVID-19-PRIMARY-CARE-SOP-COMM-PHARM-
794 PUBLICATION-V1. 2020 Apr;1: skills4pharmacy.org.uk 2020. Accessed 08/12/20]
795 113. Butt M. Covid-19 vaccination: Logistical challenges for general practice. *Practice Management*
796 2021;31(1):20-21. doi: 10.12968/prma.2021.31.1.20
797 114. National Pharmacy Association (NPA). Pharmacy on the NHS frontline 2020 [Available from:
798 https://nhsfrontline.com/coronavirus-stories-from-the-health-service-frontline. Accessed
799 17/08/2020 2020]
800 115. Pharmaceutical Services Negotiating Committee. Community Pharmacy Contractual
801 Framework: Pharmaceutical Services Negotiating Committee; 2021 [Available from:
802 https://psnc.org.uk/contract-it/the-pharmacy-contract. Accessed 08/02/21
803 116. Quinn B. UK pharmacists facing abuse and violence during lockdown. *The Guardian* 2020 Apr
804 2nd 2020. [Available from: https://www.theguardian.com/world/2020/apr/02/uk-
805 pharmacists-facing-abuse-and-violence-during-coronavirus-lockdown. Accessed 12/12/20]
806 117. Wickware C. Health board pilots delivery of flu vaccines by pharmacy technicians to manage
807 increased demand. *The Pharmaceutical Journal* 2020. doi: 10.1211/PJ.2020.20208565
808 118. Royal Pharmaceutical Society. The future of pharmacy in a sustainable NHS: Key principles for
809 transformation and growth. London: Royal Pharmaceutical Society, 2020. [Available from:
810 https://www.rpharms.com/recognition/all-our-campaigns/policy-a-z/future-of-pharmacy.
811 Accessed 08/01/21]

For peer review only

119. Sparkman A, Brookhart AL, Goode J-VKR. The impact of an immunization check-up at a pharmacist-provided employee health screening. *Journal of the American Pharmacists Association* 2017;57(3):S274-S78. doi: 10.1016/j.japh.2017.02.010

120. Rodgers RM, Gammie SM, Loo RL, et al. Comparison of pharmacist and public views and experiences of community pharmacy medicines-related services in England. *Patient Prefer Adherence* 2016;10:1749-58. doi: 10.2147/PPA.S112931

121. Hindi AMK, Schafheutle EI, Jacobs S. Patient and public perspectives of community pharmacies in the United Kingdom: A systematic review. *Health Expectations* 2017;21(2):409-28. doi: 10.1111/hex.12639

122. Letley L, Ramsay M. Discussing the MMR vaccine with patients. *The Pharmaceutical Journal* 2019. doi: 10.1211/pj.2019.20205819

123. Kember J, Hodson K, James DH. The public's perception of the role of community pharmacists in Wales. *International Journal of Pharmacy Practice* 2017;26(2):120-28. doi: 10.1111/ijpp.12375

124. Gidman W, Ward P, McGregor L. Understanding public trust in services provided by community pharmacists relative to those provided by general practitioners: a qualitative study. *BMJ open* 2012;2(3):e000939. doi: 10.1136/bmjopen-2012-000939

125. McConeghy KW, Wing C. A national examination of pharmacy-based immunization statutes and their association with influenza vaccinations and preventive health. *Vaccine* 2016. doi: 10.1016/j.vaccine.2016.04.076

126. Cheong MWL, Brock T, Karwa R, et al. COVID-19 and Clinical Pharmacy Worldwide - A Wake Up Call and a Call to Action. *JACCP JAm Coll Clin Pharm* 2020;3(5):860-63. doi: 10.1002/jac5.1286

127. Adunlin G, Murphy PZ, Manis M. COVID-19: How Can Rural Community Pharmacies Respond to the Outbreak? *J Rural Health* 2020. doi: 10.1111/jrh.12439

For peer review only

For peer review only - http://bmjopen.bmj.com/site/about/guidelines.xhtml

837 128. Aruru M, Truong HA, Clark S. Pharmacy Emergency Preparedness and Response (PEPR): a
838 proposed framework for expanding pharmacy professionals' roles and contributions to
839 emergency preparedness and response during the Covid-19 pandemic and beyond. *Research*
840 *in Social and Administrative Pharmacy* 2020;17(1):1967-77. doi:
841 10.1016/j.sapharm.2020.04.002
842 129. Fiore V. GP referrals under CPCS to go live November 1, NHSE confirms. *Chemist and Druggist*
843 2020. [Available from: https://www.chemistanddruggist.co.uk/news/-gp-referrals-under-
844 cpcs-live-november-1-nhse-says. Accessed 11/11/20]
845 130. Robinson J. Public Health England to review guidance against the use of rapid COVID-19 tests in
846 community pharmacies. *the Pharmaceutical Journal* 2020;305. doi:
847 10.1211/PJ.2020.20208550
848 131. Buchan SA, Rosella LC, Finkelstein M, et al. Impact of pharmacist administration of influenza
849 vaccines on uptake in Canada. *Canadian Medical Association Journal* 2016;189(4):E146-E52.
850 doi: 10.1503/cmaj.151027
851 132. Calo WA, Shah PD, Gilkey MB, et al. Implementing pharmacy-located HPV vaccination: findings
852 from pilot projects in five U.S. states. *Human Vaccines & Immunotherapeutics* 2019;15(7-
853 8):1831-38. doi: 10.1080/21645515.2019.1602433
854 133. Anderson C, Thornley T. "It's easier in pharmacy": why some patients prefer to pay for flu jabs
855 rather than use the National Health Service. *BMC Health Services Research* 2014;14(1). doi:
856 10.1186/1472-6963-14-35
857 134. Baroy J, Chung D, Frisch R, et al. The impact of pharmacist immunization programs on adult
858 immunization rates: a systematic review and meta-analysis. *Journal of the American*
859 *Pharmacists Association* 2016;56(4):418-26. doi: 10.1016/j.japh.2016.03.006
860 135. Anderson C, Thornley T. Who uses pharmacy for flu vaccinations? Population profiling through a
861 UK pharmacy chain. *International Journal of Clinical Pharmacy* 2016;38(2):218-22. doi:
862 10.1007/s11096-016-0255-z

For peer review only

For peer review only - http://bmjopen.bmj.com/site/about/guidelines.xhtml

136. Pharmacy Guild of Australia. 93 per cent of Aussies recognise benefits of flu shot in pharmacy– Pharmacy Guild of Australia [blog]: Pharmacy Guild of Australia 2017. [Available from: https://www.guild.org.au/news-events/news/2017/93-per-cent-of-aussies-recognise-benefits-of-flu-shot-in-pharmacy. Accessed 12/12/20]

137. Latif A, Tariq S, Abbasi N, et al. Giving Voice to the Medically Under-Served: A Qualitative Co-Production Approach to Explore Patient Medicine Experiences and Improve Services to Marginalized Communities. *Pharmacy (Basel, Switzerland)* 2018;6(1):13. doi: 10.3390/pharmacy6010013

138. Anderson C, Blenkinsopp A, Armstrong M. Feedback from community pharmacy users on the contribution of community pharmacy to improving the public's health: a systematic review of the peer reviewed and non-peer reviewed literature 1990-2002. *Health Expect* 2004;7(3):191-202. doi: 10.1111/j.1369-7625.2004.00274.x

139. Eades CE, Ferguson JS, O'Carroll RE. Public health in community pharmacy: a systematic review of pharmacist and consumer views. *BMC public health* 2011;11:582-82. doi: 10.1186/1471-2458-11-582

140. Hassell K, Rogers A, Noyce P. Community pharmacy as a primary health and self-care resource: a framework for understanding pharmacy utilization. *Health and Social Care in the Community* 2000;8(1):40-49. doi: 10.1046/j.1365-2524.2000.00222.x

141. Alkhalili M, Ma J, Grenier S. Defining Roles for Pharmacy Personnel in Disaster Response and Emergency Preparedness. *Disaster Medicine and Public Health Preparedness* 2017;11(4):496-504. doi: 10.1017/dmp.2016.172

142. Ford H, Dallas CE, Harris C. Examining Roles Pharmacists Assume in Disasters: A Content Analytic Approach. *Disaster Medicine and Public Health Preparedness* 2013;7(6):563-72. doi: 10.1017/dmp.2013.99

For peer review only

For peer review only - http://bmjopen.bmj.com/site/about/guidelines.xhtml

143. Schwerzmann J, Graitcer SB, Jester B, et al. Evaluating the Impact of Pharmacies on Pandemic Influenza Vaccine Administration. *Disaster Medicine and Public Health Preparedness* 2017;11(5):587-93. doi: 10.1017/dmp.2017.1

144. Donald M, King-Shier K, Tsuyuki RT, et al. Patient, family physician and community pharmacist perspectives on expanded pharmacy scope of practice: a qualitative study. *CMAJ Open* 2017;5(1):E205-E12. doi: 10.9778/cmajo.20160135

145. Mossialos E, Courtin E, Naci H, et al. From “retailers” to health care providers: Transforming the role of community pharmacists in chronic disease management. *Health Policy* 2015;119(5):628-39. doi: 10.1016/j.healthpol.2015.02.007

146. Mossialos E, Naci H, Courtin E. Expanding the role of community pharmacists: Policymaking in the absence of policy-relevant evidence? *Health Policy* 2013;111(2):135-48. doi: 10.1016/j.healthpol.2013.04.003

147. OECD (2020), Beyond Containment: Health systems responses to COVID-19 in the OECD. Organisation for Economic Co-operation and Development, Paris. [Available from: https://read.oecd-ilibrary.org/view/?ref=119_119689-ud5comtf84&title=Beyond_Containment:Health_systems_responses_to_COVID-19_in_the_OECD. Accessed 13/4/21]

148. Ilardo ML, Speciale A. The Community Pharmacist: Perceived Barriers and Patient-Centered Care Communication. *International Journal of Environmental Research and Public Health* 2020;17(2):536. doi: 10.3390/ijerph17020536

149. Daly CJ, Quinn B, Mak A, et al. Community Pharmacists’ Perceptions of Patient Care Services within an Enhanced Service Network. *Pharmacy* 2020;8(3):172. doi: 10.3390/pharmacy8030172

150. Ecarnot F, Crepaldi G, Juvin P, et al. Pharmacy-based interventions to increase vaccine uptake: report of a multidisciplinary stakeholders meeting. *BMC Public Health* 2019;19(1) doi: 10.1186/s12889-019-8044-y

For peer review only - http://bmjopen.bmj.com/site/about/guidelines.xhtml

913 151. Moullin JC, Sabater-Hernández D, Fernandez-Llimos F, et al. Defining professional pharmacy services in community pharmacy. *Research in Social and Administrative Pharmacy* 2013;9(6):989-95. doi: 10.1016/j.sapharm.2013.02.005

916 152. NHS England. General practice forward view: NHS England, 2016. [Available from: https://www.england.nhs.uk/gp/gpfv. Accessed 12/12/20]

918 153. Milosavljevic A, Aspden T, Harrison J. Community pharmacist-led interventions and their impact on patients' medication adherence and other health outcomes: a systematic review. *The International journal of pharmacy practice* 2018;26(5):387-97. doi: 10.1111/ijpp.12462 [published Online First: 2018/06/22]

922 154. Justwan F, Baumgaertner B, Carlisle JE, et al. The effect of trust and proximity on vaccine propensity. *PLOS One* 2019;14(8):e0220658. doi: 10.1371/journal.pone.0220658

924 155. Pharmaceutical Services Negotiating Committee. Preparations for a potential COVID-19 vaccination programme.: Pharmaceutical Services Negotiating Committee; 2020 [Available from: https://psnc.org.uk/our-news/preparations-for-a-potential-covid-19-vaccination-programme. Accessed 08/11/2020]

For peer review only - http://bmjopen.bmj.com/site/about/guidelines.xhtml

Appendix 1 - OVID MEDLINE – Search Strategy

1. exp Coronavirus/ or exp Coronavirus Infections/ or (coronavirus* or "2019-nCoV" or "2019 ncov" or nCov or "Covid 19" or Covid19 or "SARS CoV 2" or novel coronavirus or novel corona virus or covid* or "coronavirus 2" or coronavirus infection* or coronavirus disease or corona virus disease or new coronavirus or new corona virus or new coronaviruses or novel coronaviruses or wuhan).mp. [mp=title, abstract, original title, name of substance word, subject heading word, floating sub-heading word, keyword heading word, organism supplementary concept word, protocol supplementary concept word, rare disease supplementary concept word, unique identifier, synonyms]

2. (Community pharmacy or community pharmacies or community pharmacist or community pharmacists or retail pharmacy or retail pharmacist or retail pharmacists or dispensing chemist or dispensing chemists or (((Pharmacist or pharmacy) and (community or primary care)) or dispensary or apothecary or druggist)).mp. [mp=title, abstract, original title, name of substance word, subject heading word, floating sub-heading word, keyword heading word, organism supplementary concept word, protocol supplementary concept word, rare disease supplementary concept word, unique identifier, synonyms]

3. 1 and 2

NB. No Date or Language Limits were applied.

For peer review only

Appendix 2 - Literature inclusion criteria

Published after January 2003 AND

COVID-19 or other pandemic or other infectious diseases

OR vaccination programmes

OR expanded/extended roles

AND Community Pharmacy

AND High- or middle-income country

For peer review only

PRISMA 2009 Flow Diagram

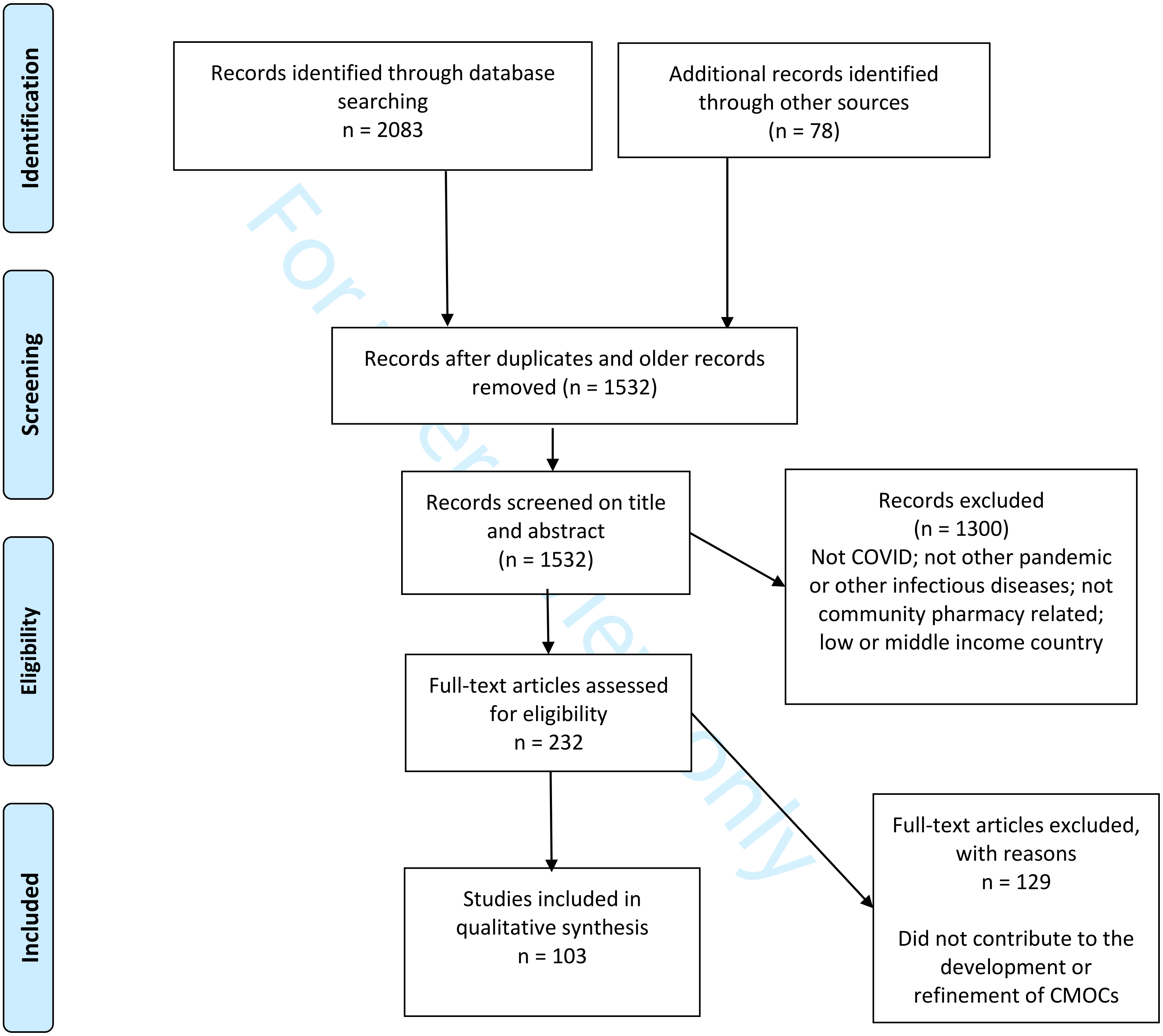

From: Moher D, Liberati A, Tetzlaff J, Altman DG, The PRISMA Group (2009). *P*referred *R*eporting *I*tems for *S*ystematic Reviews and *M*eta-*A*nalyses: The PRISMA Statement. PLoS Med 6(7): e1000097. doi:10.1371/journal.pmed1000097

For more information, visit www.prisma-statement.org.

For peer review only - http://bmjopen.bmj.com/site/about/guidelines.xhtml

Appendix 4 - A simplified diagram of the programme theory of a COVID-19 vaccination programme provided by community pharmacies

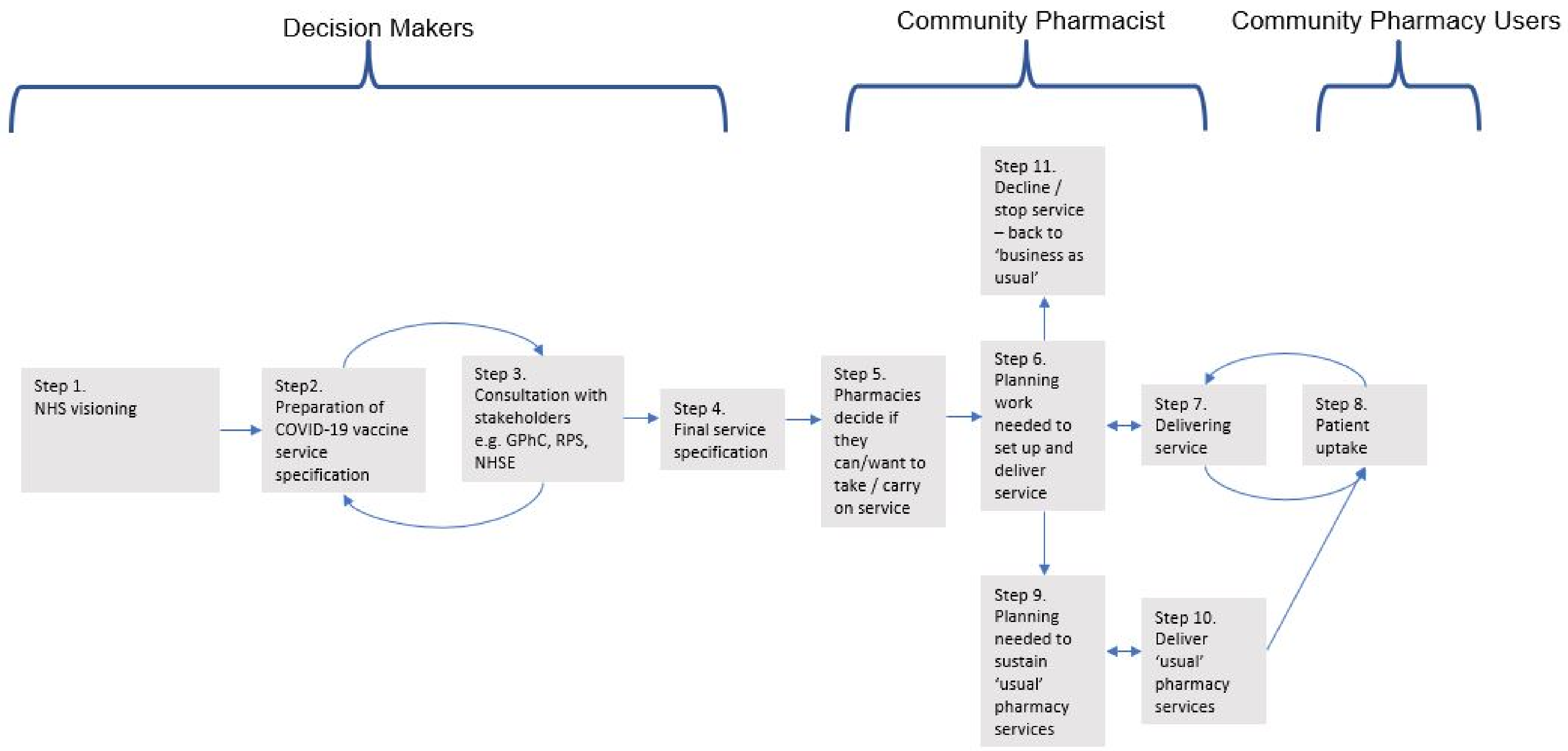

Supplement: Author's manuscript [file bmjopen-2021-050043.draft_revisions.pdf]
